# Terminal N_2_ Dissociation in [(PNN)Fe(N_2_)]_2_(μ‐N_2_) Leads to Local Spin‐State Changes and Augmented Bridging N_2_ Activation

**DOI:** 10.1002/chem.202202172

**Published:** 2022-08-18

**Authors:** Nicolas I. Regenauer, Hubert Wadepohl, Dragoş‐Adrian Roşca

**Affiliations:** ^1^ Anorganisch-Chemisches Institut Universität Heidelberg Im Neuenheimer Feld 276 Germany

**Keywords:** iron, nitrogen fixation, nitrides, redox-active ligands, spin change

## Abstract

Nitrogen fixation at iron centres is a fundamental catalytic step for N_2_ utilisation, relevant to biological (nitrogenase) and industrial (Haber‐Bosch) processes. This step is coupled with important electronic structure changes which are currently poorly understood. We show here for the first time that terminal dinitrogen dissociation from iron complexes that coordinate N_2_ in a terminal and bridging fashion leaves the Fe‐N_2_‐Fe unit intact but significantly enhances the degree of N_2_ activation (Δν≈180 cm^−1^, Raman spectroscopy) through charge redistribution. The transformation proceeds with local spin state change at the iron centre (S=1/2 →S=^3^/_2_). Further dissociation of the bridging N_2_ can be induced under thermolytic conditions, triggering a disproportionation reaction, from which the tetrahedral (PNN)_2_Fe could be isolated. This work shows that dinitrogen activation can be induced in the absence of external chemical stimuli such as reducing agents or Lewis acids.

## Graphical abstract

**Small stimulus, big change**. Vacuum‐induced reversible dissociation of terminal N_2_‐units lead to structural and electronic reorganisation resulting in a significant activation of a bridging N_2_ ligand.

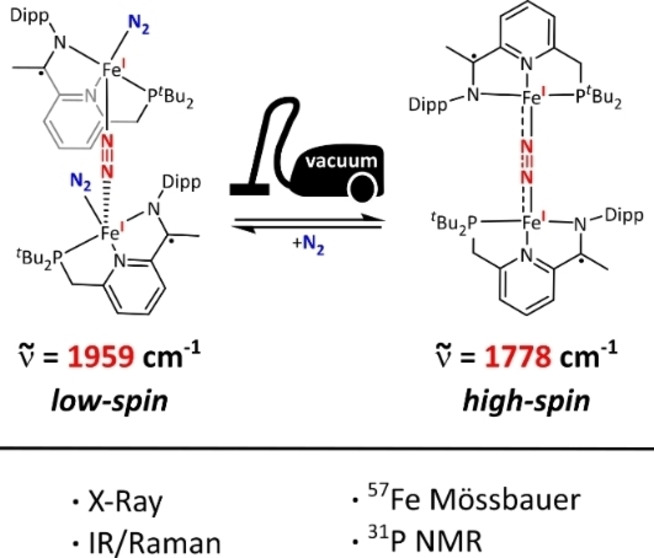

## Introduction

**Figure 1:**
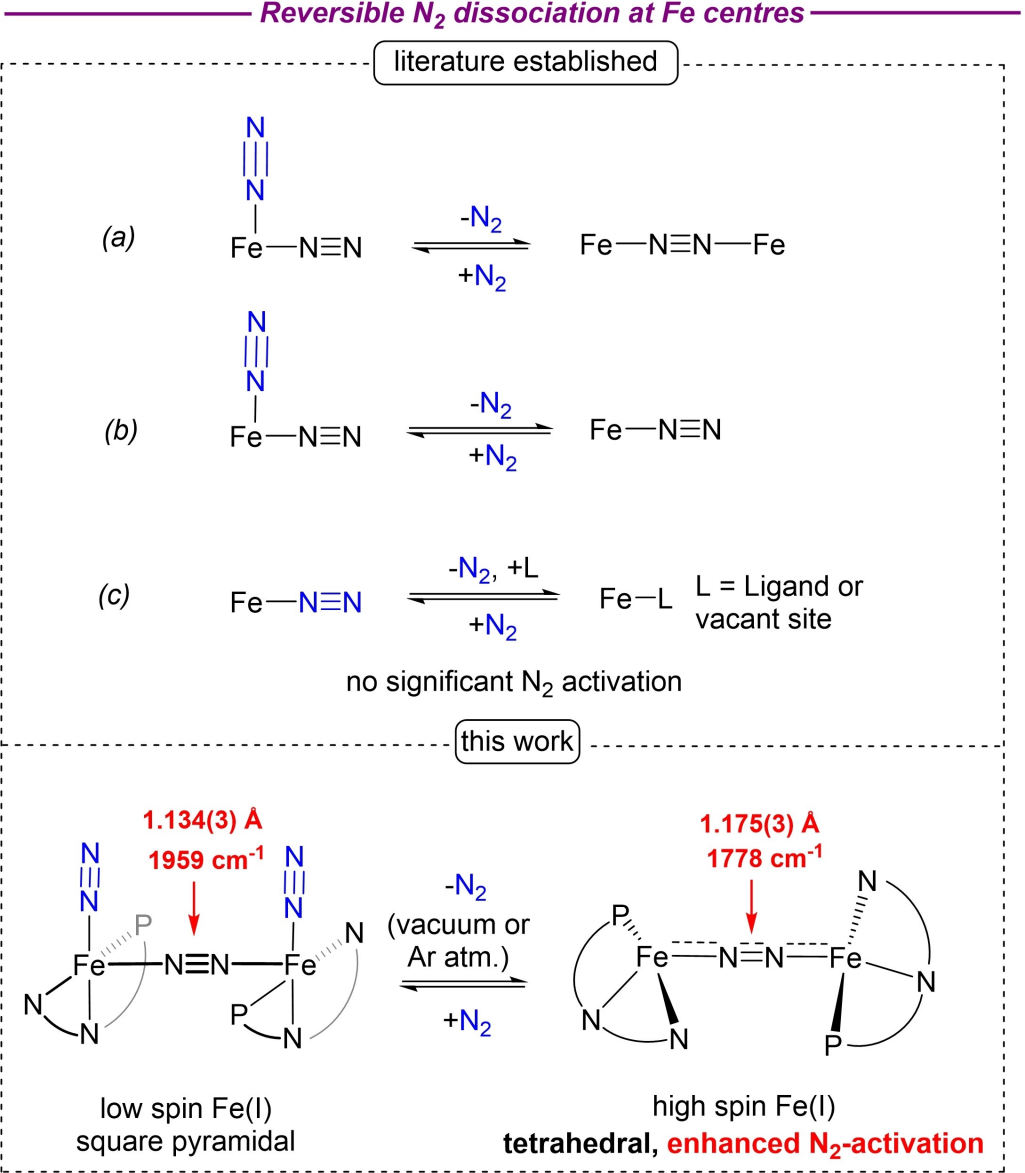
Reversible dissociation of dinitrogen in iron compounds.

While dinitrogen is the most abundant gas in our atmosphere, its direct utilisation for the synthesis of life‐sustaining nitrogen‐based building blocks is kinetically disfavoured. Nevertheless, heterogenous (Haber‐Bosch) or enzymatic (nitrogenase) processes are known to catalytically convert N_2_ gas into essential nitrogen‐containing compounds.^[1]^ Iron is at the heart of all these catalytic systems: it is the only metal present in all three of the known nitrogenases, while the Mittasch catalyst currently employed in industrial ammonia synthesis is typically based on a reduced iron/alkali metal surface. In all these processes, dinitrogen binding to iron centres represents a fundamental step which triggers important local geometry, charge distribution and spin state changes.[^2^, ^3^, ^4^] For example, recent calculations suggest that a high spin to low spin change in the E4 intermediate of nitrogenase is crucial for dinitrogen binding, where both terminal and bridging N_2_‐binding modes are possible.^[2d]^ Nevertheless, despite the considerable number of bridging and terminal iron dinitrogen complexes characterised, the electronic structure changes during these N_2_‐binding processes are not well understood. Depending on the binding mode of dinitrogen in reactants and products, reversible nitrogen binding reactions on iron centres can be roughly categorised into three groups (Figure 1): (a) Dissociation of one terminally bound dinitrogen molecule from *η* ^1^‐N_2_ bound Fe(N_2_)_x_ fragments, to give *μ* ^2^‐*η* ^1^:*η* ^1^‐bound N_2_ systems.^[5]^ (b) Dissociation of terminally bound dinitrogen molecule from Fe(N_2_)_2_ fragments to give Fe(N_2_) systems, where the N_2_ is terminally bound.^[6]^ These N_2_‐based equilibria usually proceed with minimal N_2_ activation. (c) Dissociation of terminally bound N_2_ from Fe(N_2_) fragments which leaves a formal vacant site at the metal centre. In some cases, this vacant site can then be occupied by intramolecular metal‐ligand interactions of β‐agostic or π‐nature.[^1d^, ^7^] This latter mode is also the most encountered.

Here we describe a new type of N_2_‐binding equilibrium at iron centres, which involves reversible dissociation of terminally bound N_2_ ligands from terminal/bridging end‐on FeN_2_ complexes to give bridging‐only end‐on FeN_2_ species. Remarkably, this type of N_2_ binding equilibrium is accompanied by a significant distortion/activation of the N_2_ bridge, which thus occurs in the absence of reducing agents or Lewis acids.^[8]^

## Results and Discussion

**Scheme 1:**
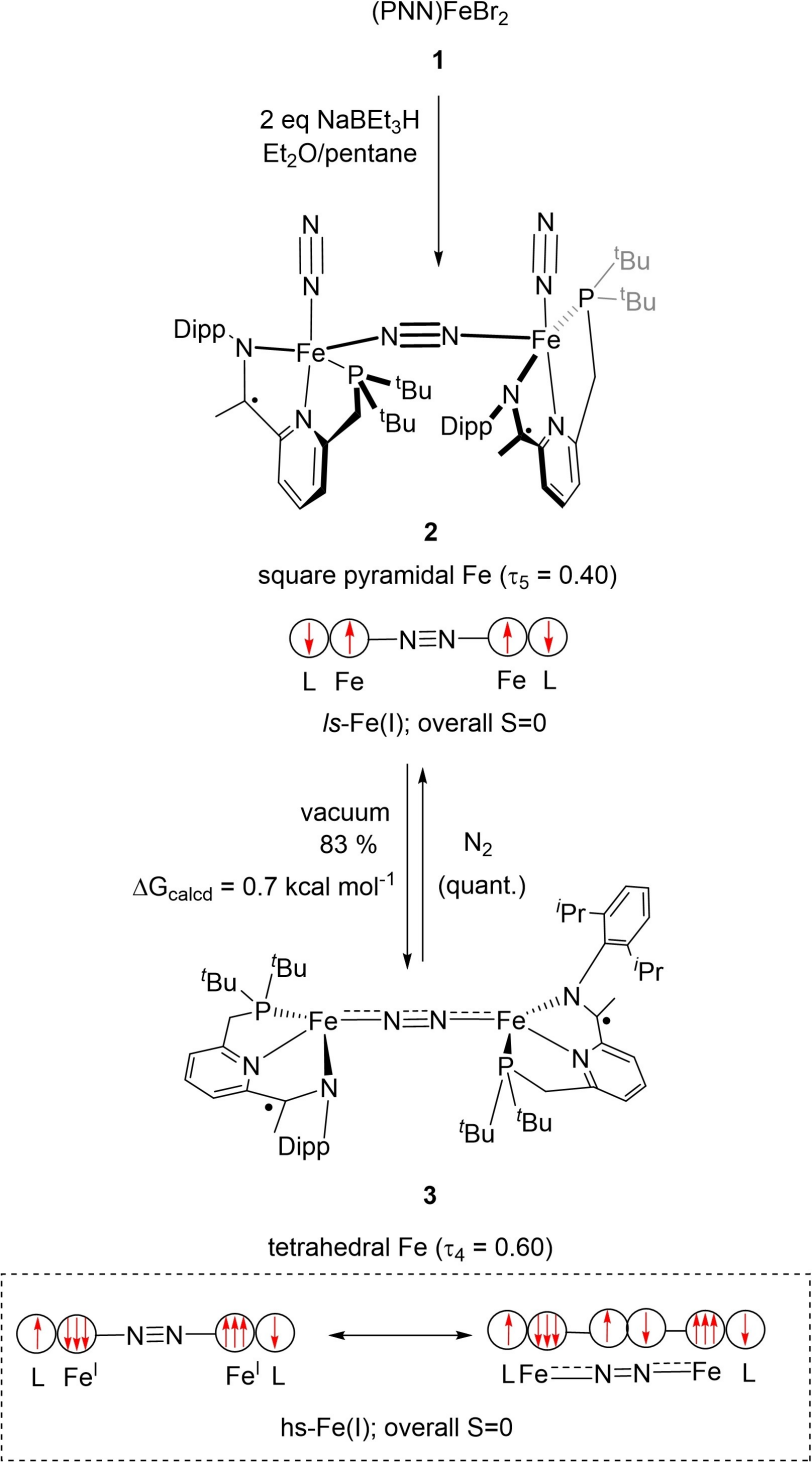
Preparation of tetrahedral dinitrogen‐bridged complex **3** via terminal dinitrogen dissociation from **2**. Spin paring schemes for **2** and **3** are depicted, illustrating the spin state change at the iron centres.

**Figure 2:**
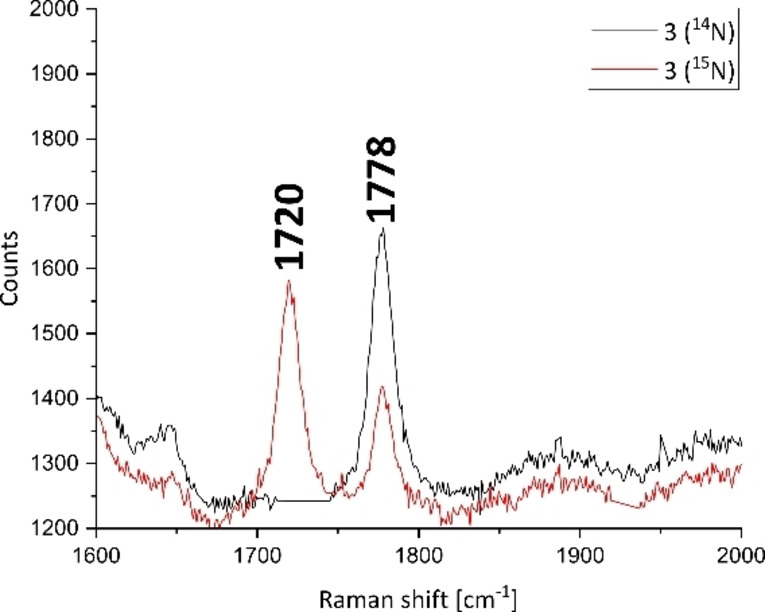
Raman spectra of **3** (black) and **3**‐^15^N_2_ (red) obtained with 532 nm excitation in solid state. For the full spectrum, see the Supporting Information. Absorption dips resulting from the materials used for sample preparation were removed to ensure clarity of the spectra. Full unedited copies of the spectra are given as Figures S24–S27 in the Supporting Information.

We have recently demonstrated that tridentate phosphine α‐iminopyridine (PNN) iron complexes readily coordinate dinitrogen in a bridging and a terminal fashion, leading to the isolation of [(PNN)Fe(N_2_)]_2_(μ‐N_2_) **2**.^[9]^ Albeit stable under an atmosphere of N_2_, storing **2** under an atmosphere of argon either as a solid or in solution triggers a colour change from green to red‐brown. Monitoring by ^31^P NMR spectroscopy indicated the formation of a second species **3** (ca. 16 %). The conversion of **2** to **3** could be increased to 83 % by repeatedly dissolving mixtures of **2** and **3** in hexane under an argon atmosphere, followed by solvent removal (Scheme 1). Notably, the measured ^31^P{^1^H} resonance for **3** (δ_P_ 28.7 ppm) is significantly shifted compared to the one measured for **2** (δ_P_ 113.7 ppm). This change is nevertheless reversible and placing samples of **3** under an atmosphere of N_2_ regenerates **2** within seconds. Interestingly, no intermediate was observed in which only one of the terminal dinitrogen ligands had dissociated.^[10]^ Single crystal X‐ray diffraction on crystalline samples of **3**, obtained from concentrated Et_2_O/hexane solutions at −40 °C under argon atmosphere allowed us to identify **3** as a centrosymmetric dinuclear iron complex, where the two tetracoordinated iron centres are bridged by a N_2_ molecule. While the IR spectrum of **3** is featureless in the regions expected for N−N stretching bands, information about the degree of N_2_ activation could be obtained by Raman spectroscopy where an absorption at 1778 cm^−1^ was assigned to the bridging N_2_ ligand. This band shifts to 1720 cm^−1^ when a ^15^N_2_‐enriched sample was used (Figure 2). This suggests that the N_2_ ligand is strongly activated and is identical to the one reported for neutral iron(I) dinitrogen complexes based on β‐diketiminate ligands reported by Holland[^11a^, ^11b^] or on tris(pyrazolyl)borate (Tp) ligands by Harman.^[11c]^ Moreover, the position of the $\tilde{\upsilon }$_*N2*_ is the lowest ever reported for an iminopyridine‐based iron system.

The bridging N_2_ stretching frequency in the starting material **2** is also Raman, as well as IR active and was located at 1959 cm^−1^ (see the Supporting Information). Therefore, the dissociation of the terminal N_2_ ligands in **2** induces an activation of the bridging N_2_ ligand, characterised by a remarkable bathochromic shift of ca. 180 cm^−1^. Such a shift of a dinitrogen band triggered by simple ligand dissociation in the absence of strongly reducing agents or Lewis acids has not been reported, to the best of our knowledge, for any other metal‐N_2_ complex.

**Figure 3:**
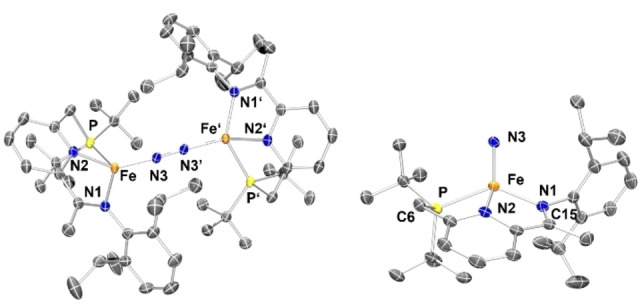
Molecular structure of **3** (left). Representation of the monomeric unit of **3**, highlighting the tetrahedral geometry at the iron centre (right).

**Figure 4:**
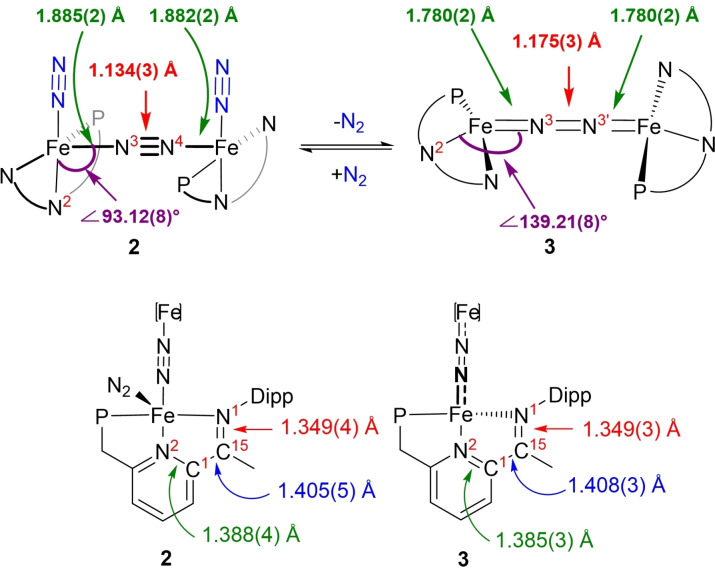
Comparison of metrical parameters in pentacoordinated (**2**) and tetracoordinated (**3**) bridging dinitrogen complexes.

Comparing the metric data of **2** and **3** (Figures 3 and 4) revealed important structural differences: *(i)* As a result of the dissociation of the terminal N_2_ ligands, the degree of activation of the remaining bridging N_2_ ligand increases, as reflected in the marked elongation of the bridging N=N separation (1.175(3) Å in **3** vs. 1.134(3) Å in **2**),^[12]^ which is also corroborated by the data obtained from Raman spectroscopy. Such an increase (Δd_NN_ ∼0.04 Å) is comparable to the one measured by Szymczak in B(C_6_F_5_)_3_ functionalization of Fe(depe)_2_(N_2_) complexes (depe=1,2‐bis(diethylphosphino)‐ethane).^[8]^ The N=N bond length in **3** is comparable to the one measured for neutral bridging Fe‐N_2_ complexes supported by β‐diketiminate (nacnac) or Tp ligands^11^ and significantly more elongated compared to the (PDI)Fe and (CNC)Fe (CNC=bis(arylimidazol‐2‐ylidene)pyridine) analogues.^[13]^ The increase in Fe−N covalency is also manifested in the significant contraction of the Fe‐μ‐η^1^:η^1^‐N_2_ distances from an average value of 1.883(2) Å in **2** to 1.780(2) Å in **3** (Figure 4). These phenomena imply a significant increase of backbonding contributions from the Fe centres to the bridging N_2_ ligand. Interestingly, the dissociation of the terminal N_2_ ligands does not exert significant changes on the key metric descriptors of the chelate (i. e. no significant changes in the N^1^=C^15^, C^1^‐C^15^ and C^1^=N^2^ bond lengths (Figure 4),^[14]^ suggesting no increase of Fe→PNN backbonding in **3** compared to **2**. *(ii)* The local geometry at the iron centre changes from square pyramidal in **2** (τ_5_’=0.40) to distorted tetrahedral in **3** (τ_4_’=0.60).[^15^, ^16^] This behaviour contrasts with the one previously reported for dinitrogen dissociation from square pyramidal iron centres, which yield square planar complexes.[^3a^, ^3b^, ^4^] The significant geometrical reorganisation of the PNN chelate and ancillary ligands is also reflected in the marked change of the ∠N^2^FeN^3^ from 92.74(12)° in **2** to 139.21(8)° in **3** (Figure 4). This change ensures that the bridging N_2_ ligand is effectively kinetically shielded (see the Supporting Information for a space filling plot).

The change in geometry at the iron centre as well as the marked difference in δ_P_ between **2** and **3** (Δδ_P_=85 ppm) suggests that the terminal N_2_ dissociation is accompanied by significant changes in electronic structure. This prompted us to investigate these differences in more detail, by relying primarily on crystallographic, Mössbauer and NMR data, which we have correlated with computational modelling.

**Figure 5:**
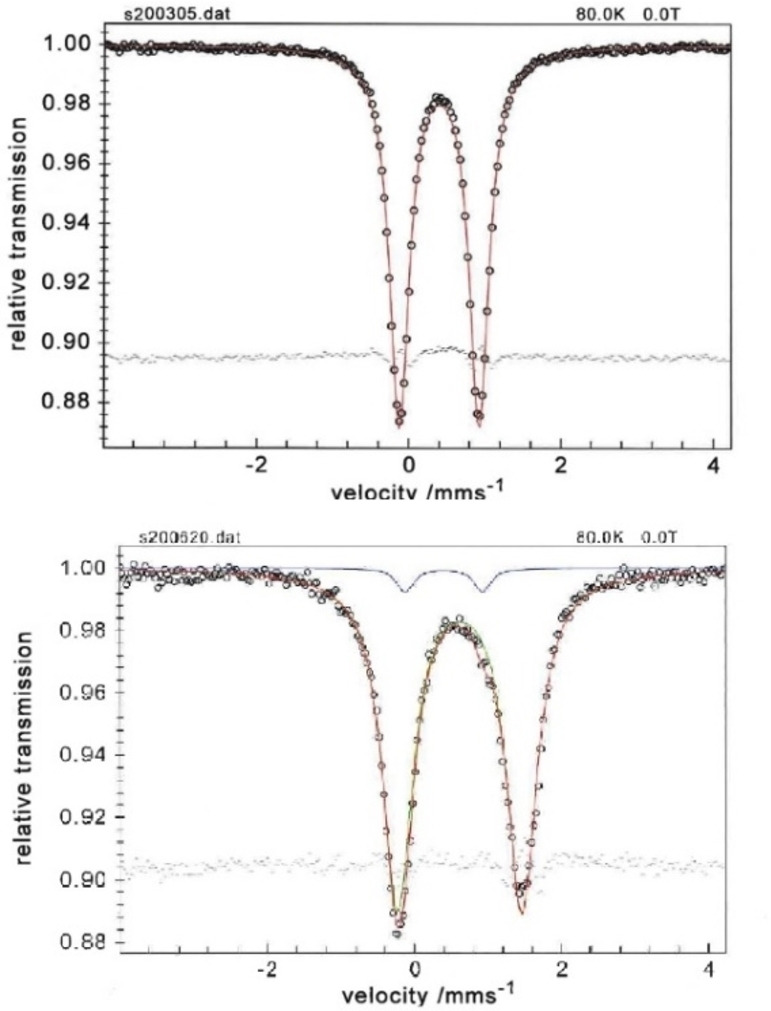
Zero‐field Mössbauer spectra of **2** (top) and **3** (bottom) recorded at 80 K. The red line represents a fit with a Lorentzian quadrupole doublet. Parameters for **2**: δ=0.40 mm s^−1^ |ΔE_Q_|=1.05 mm s^−1^. Parameters for **3**: δ=0.62 mm s^−1^ |ΔE_Q_|=1.69 mm s^−1^ (small amounts of **2** [4 %, blue line], correspond to the observed minor component).

Fitting the data obtained from zero‐field Mössbauer spectroscopy (80 K) for **2** yielded an isomer shift (δ) of 0.40 mm s^−1^ and a quadrupole splitting (|ΔE_Q_|) of 1.05 mm s^−1^ (Figure 5). These values are similar to the ones previously observed for square pyramidal (PDI)Fe(N_2_)_2_ (PDI=pyridinediimine) complexes.^[ 17]^ We have previously modelled the ground state of **2** by DFT calculations, where the broken‐symmetry (BS) methodology was used to account for ligand non‐innocence.^[9]^ A solution with the following pairing scheme L^↑^‐Fe^↓^‐N_2_‐Fe^↑^‐L^↓^, corresponding to BS(1,1)^[18]^ for each Fe‐PNN unit was found to be the lowest in energy. This solution is therefore consistent with a low‐spin Fe(I) centre (S=1/2 ), antiferromagnetically coupled with a ligand radical (S=1/2 ), in line with the observed square pyramidal local geometry at the iron centre. Using this solution for the calculation of the Mössbauer parameters yields δ_calcd_=0.43 mm s^−1^ and ΔE_Q(calcd.)_=1.25 mm s^−1^, in excellent agreement with the experimental data.^[19]^ In order to further assess the validity of this solution for describing the ground state of **2**, we have also calculated the NMR ^31^P chemical shift. The calculated value δ_31P(calcd.)_=122.4 ppm reproduces well the experimental value (δ_31P(exp.)_=113.7 ppm). An alternative description of **2** would be as a closed‐shell structure where no ligand‐based redox activity is assumed. However, this solution is 12.4 kcal mol^−1^ higher in energy compared to the BS(1,1) model.^[9]^ Furthermore, the calculated Mössbauer and NMR data based on the closed‐shell solution deviate significantly from the experimental values (Table 1). Subsequently, we proceeded to investigate the electronic structure of **3**. The isomer shift value obtained from Mössbauer spectroscopy (δ=0.62 mm s^−1^_,_ Figure 5) is significantly different from the one measured for **2** (Δδ=0.22). Note that the isomer shift increases, despite the increase in iron – ligand covalency. Nevertheless, the isomer shift value for **3** is comparable to values measured for high‐spin Fe(I) centres.[^16c^, ^20^]

**Table 1:** Comparison of experimentally determined Mössbauer, NMR and metric parameters with the ones obtained based on the computational modelling of various possible ground states.

Compound	Input Method	Resulting Spin‐Pairing Scheme	Mössbauer^[c]^		NMR^[d]^	τ (X‐ray)^[e]^
			δ^[a]^	|ΔE_Q_|^[a]^	δ_31P_^[b]^	
**2**	Experimental	–	0.40	1.05	113.7	0.40
	RKS ‐ Fe(0)^[f]^	–	0.31	1.26	139.1	0.41
	BS(1,1) ‐*ls*‐Fe(I)^[f]^	L^↑^−Fe^↓^−N_2_−Fe^↑^−L^↓^	0.43	1.25	122.4	0.38
**3**	Experimental	–	0.62	1.69	28.7	0.60
	RKS – Fe(0)^[f]^	–	0.33	1.82	162.6	0.49
	UKS‐*ls*‐Fe(I)^[f]^	L^↑^−Fe^↓^−N_2_−Fe^↓^−L^↑^	0.46	0.60	126.7	0.32
	BS(1,1) – *hs*‐Fe(I)^[f]^	L^↑^−Fe^↓↓↓^−N_2_−Fe^↑↑↑^−L^↓^	0.66	1.60	79.0	0.55

[a] in mm s^−1^ ; [b] in ppm; [c] Calculated Mössbauer parameters B3LYP/TZVP//CP(PPP) for Fe. [d] Calculated δ_31P_‐ TPSS0/pcSseg‐2. [e] averaged value over both iron centres. [f] Geometry optimisations: B3LYP/SVP//TZVP(‐f) for Fe, N and P.

With the spectroscopic data at hand, we proceeded in addressing possible ground states for **3** by computational modelling. Using a BS(1,1) approach, a solution corresponding to the following spin paring scheme L^↑^‐Fe^↓↓↓^‐N_2_‐Fe^↑↑↑^‐L^↓^ was found to be the lowest in energy and reproduces well the geometry around the iron centre (τ_4_’_(calcd)._=0.55, τ_4_’_(exp)_=0.60). This solution corresponds to a *high‐spin* Fe(I) complex (S=^3^/_2_). Importantly, the calculated Mössbauer parameters (δ, ΔE_Q_) using this solution as an input are in excellent agreement with the measured values. Attempting to model **3** as a low‐spin iron complex (open‐shell singlet) did not reproduce the distorted tetrahedral geometry, yielding instead a square planar geometry around the metal centre, as expected for low spin complexes. The calculated Mössbauer parameters arising from this latter solution yielded significant deviations from the experimental values (Table 1). The same trend was observed when using various modelled ground states to calculate ^31^P chemical shifts for **3**. While NMR calculations based on the *hs*‐Fe(I) solution reproduced only modestly the experimental value (δ_31P(exp)_=28.7 ppm, δ_31P(calcd)_=79.0 ppm), these data are in significantly better agreement with experiment compared to the low‐spin ground states considered (Table 1).

**Figure 6:**
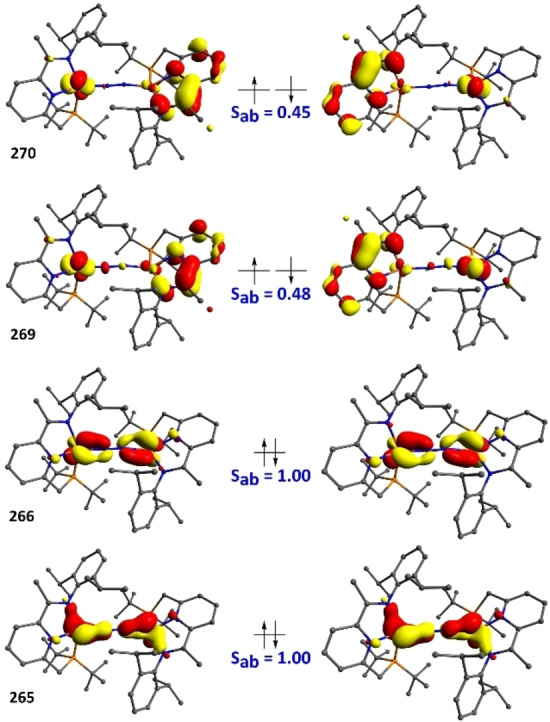
Qualitative representation of selected magnetically coupled orbitals (UCOs)^[23]^ in **2**. For a full picture, see the Supporting Information.

**Figure 7:**
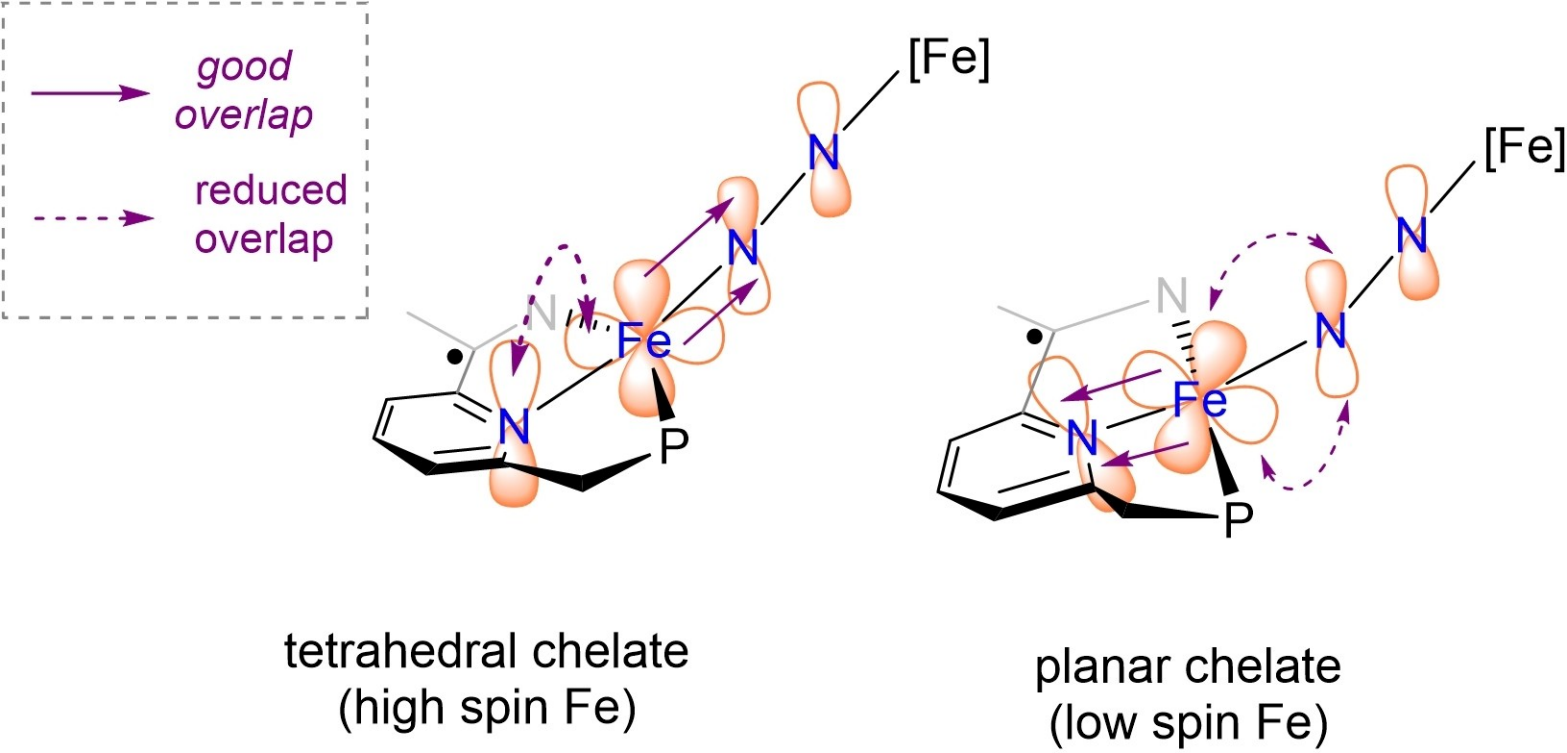
Simplified schematic representation of the π*(PNN)‐d_π_(Fe)‐π*(N_2_) interactions in **3**: tetrahedral (left, experimental) and planar (right, hypothetic) (PNN)Fe arrangement.

**Figure 8:**
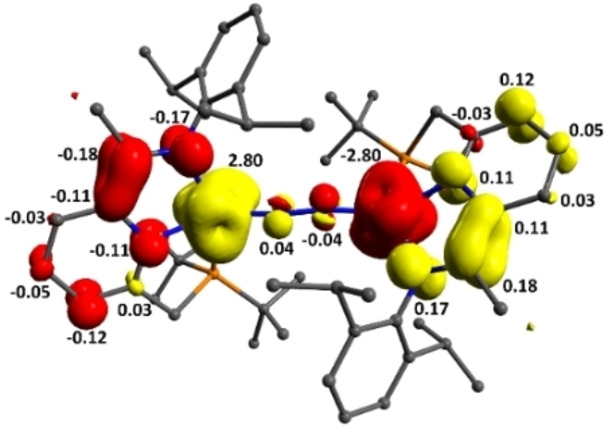
Spin density plot (LPA) for **3** showing spin delocalisation over the PNN system and the bridging N_2_ ligand.

**Figure 9:**
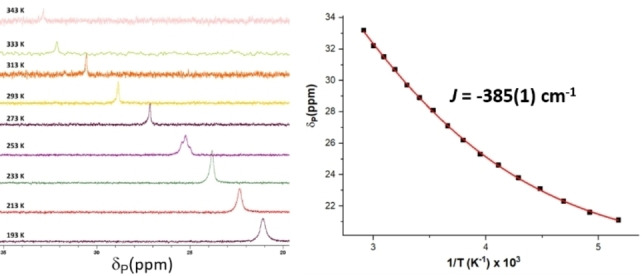
(left) ^31^P{^1^H} NMR spectrum of **3** (toluene‐d_8_) measured between −80 °C and +70 °C showing the temperature dependence of the phosphorus shift. (right) Plot of δ_P_ (ppm) vs. 1000/T (K^−1^) indicating non‐Curie behaviour. A fit using the Boltzmann function for a singlet‐triplet (based on the notation ${\hat{H}}_{HDvV}=-2J\hat{S_{1}}\;•\hat{S_{2}}$ obtained from the Heisenberg‐Dirac‐van Vleck Hamiltonian). A similar value was obtained by fitting ^1^H NMR resonances (see the Supporting Information).

In line with the spin paring scheme L^↑^‐Fe^↓↓↓^‐N_2_‐Fe^↑↑↑^‐L^↓^, one of the unpaired electrons on each iron centre (d_x2‐y2_) is antiferromagnetically coupled with a ligand‐based unpaired electron (S_αβ_=0.45 and 0.48) (Figure 6). In addition, each iron centre possesses two unpaired electrons located in d_z2_ and d_π_ orbitals which cannot interact on symmetry grounds but are non‐orthogonal with respect to the p_x_ and p_y_ orbitals of the bridging nitrogen ligands. The remaining d_π_ iron‐based orbitals are stabilised through overlap with the π*(N_2_) and are therefore doubly occupied. The linear arrangement of the Fe‐N−N‐Fe fragment (∠FeNN 176.52(2)°) maximises d_π_(Fe)‐π*(N_2_) orbital overlap which increases backbonding contributions, rendering the Fe‐N_2_‐Fe fragment highly covalent. Moreover, a deviation from planarity in the PNN scaffold reduces the extent of the d_π_(Fe)‐π*(PNN) overlap, effectively enhancing the d_π_(Fe)‐π*(N_2_) backbonding (Figure 7). While it is likely that this bonding picture is an oversimplification, it is consistent with the substantial elongation of the N−N (1.175(3) Å**)** bond and significant contraction of the Fe‐N_2_ bonds (1.780(2) Å). A Löwdin spin population analysis reveals extended delocalisation of the spin density over the entire π‐system, with alternating antiparallel distribution of spin densities on the PNN, Fe and bridging N_2_ ligands. Partial spin delocalisation over the N_2_ ligand might also indicate weak magnetic exchange through the bridge, accounting for the observed diamagnetic ground state (Figure 8). Antiferromagnetic coupling in Fe−N≡N−Fe fragments, either direct or through a N_2_ bridge has been previously reported.[^3a^, ^20^, ^21^] This formulation of **3** as a S=0 complex, exhibiting antiferromagnetic coupling is also in line with the magnetic properties determined by NMR spectroscopy. Characteristic δ_H_ chemical shifts in the typical diamagnetic region were recorded by ^1^H NMR spectroscopy; however, significant line broadening at high (above 40 °C) and low (below −40 °C) temperatures precluded the extraction of more detailed information. On the other hand, the ^31^P NMR chemical shifts are sharp and display significant change with respect to temperature in the range of −80 °C to +70 °C (ca. 12 ppm, Figure 9). This temperature dependence of chemical shifts hints at mixing of thermally accessible triplet states into the diamagnetic ground state. From the variable temperature NMR data, a singlet‐triplet gap of 2.21(2) kcal mol^−1^ can be calculated by fitting a magnetisation function to the experimental data (see the Supporting Information). In agreement with the experiment, a small energy difference (1.1 kcal mol^−1^) between the singlet and the triplet state was calculated by DFT methods.^[22]^ In contrast to **3**, the ^31^P NMR chemical shifts of **2** change only marginally in the temperature range −80 °C–+40 °C (by ca. 1 ppm).^[9]^ Above 40 °C, the resonances corresponding to **2** become increasingly broad (Δν_1/2_=150 Hz at 40 °C) and partial conversion of **2** to **3** can be observed by ^31^P NMR spectroscopy.

The calculated electronic structure of **2** and **3** suggests that dinitrogen dissociation induces a spin change at the individual iron centres (from 1/2 to ^3^/_2_) but the overall spin state of the molecule (S=0) is conserved. The terminal nitrogen dissociation is thermoneutral/ slightly endergonic (ΔG=0.7 kcal mol^−1^).

**Scheme 2:**
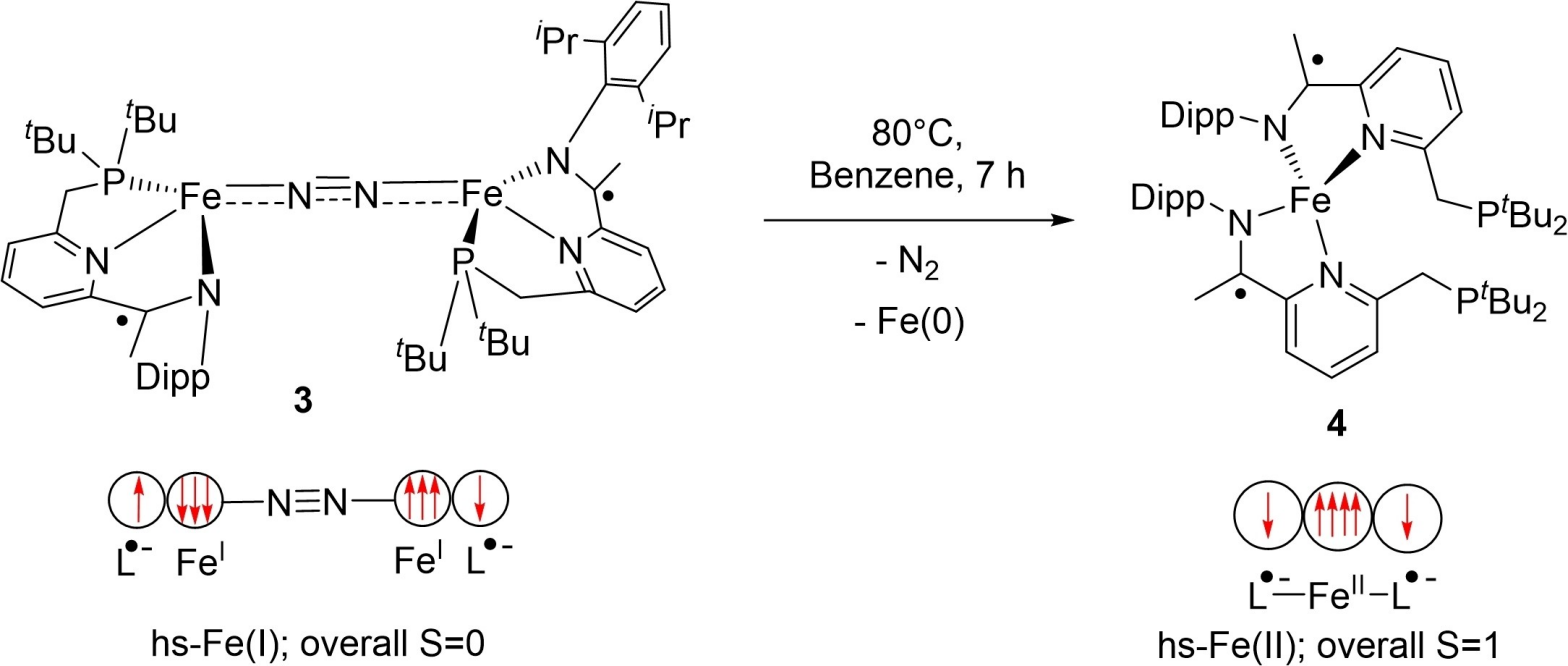
Thermally induced dinitrogen extrusion and disproportionation from **3**.

**Figure 10:**
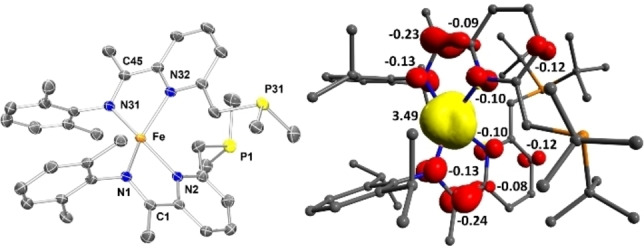
(left) Molecular structure of **4**. The ^*i*^Pr and ^*t*^Bu groups are truncated for clarity. For the complete representation, see the Supporting Information. (right) Spin density plot (LPA) for **4**, arising from a BS(4,2)‐DFT solution, showing spin delocalisation over the PNN system.

While compound **3** is stable at room temperature in solution for at least two weeks, prolonged heating at temperatures over 80 °C (for 7 h) in benzene‐d_6_ yields a new well‐defined paramagnetic species (**4**) (Scheme 2) which displays resonances between 207 and −324 ppm in the ^1^H NMR spectrum. The same product is formed, albeit more slowly, by irradiating samples of **3** (390 nm) at room temperature for 16 h. Single crystal X‐ray diffraction confirmed **4** as a tetrahedral (τ_4_=0.66) iron centre chelated by two κ^2^‐bis(imino)pyridine ligands (Figure 10). The product, formed by N_2_ extrusion from **3**, followed by disproportionation, is reminiscent of Chirik's neutral (^Et^PDI)_2_Fe (^Et^PDI=2,6‐diethyl‐substituted pyridinediimine) complex, which was obtained directly from reducing the corresponding iron dihalide in the presence of Na/Hg.^24^ Solution magnetic moment measurements on **4** (Evans’ method, μ_eff_=3.4(2)μ_B_) are consistent with an overall S=1 structure, implying an antiferromagnetic coupling between a *hs*‐Fe(II) (S=2) centre and each of the PNN chelates, which display monoradical character.

The radical character of the ligand in complex **4** could also be verified through computational studies. **4** was best modelled through a broken symmetry approach, where a BS(4,2) solution was the lowest in energy. This solution is consistent with a high‐spin ferrous centre, antiferromagnetically coupled with each of the ligand radicals. This coupling is also evident from the spin population analysis, which displays antiparallel spin alignment between the iron‐ and PNN‐based unpaired electrons (Figure 10).

The formation of **4** under thermolytic and photolytic conditions suggests that the N_2_ extrusion reaction followed by disproportionation is favoured, while a six‐electron transfer reaction, which would yield an open‐shell iron nitride complex is energetically prohibitive. In contrast to iron chemistry, benzylphosphine‐pyridine scaffolds are successfully employed in dinitrogen splitting on molybdenum and rhenium.[^25^, ^26^] These reactions usually afford *closed‐shell* stable metal nitrido complexes. Often, these species are thermodynamic sinks in the N_2_ splitting reaction which renders difficult the catalytic incorporation of the N‐fragments obtained from N_2_ into organic substrates. In contrast, dinitrogen splitting to generate terminal metal nitride complexes remains an unrealised goal for first row transition metals (iron included),[^1a^, ^27^] despite the relevance of this elementary step for the Haber‐Bosch process.^[28]^ In the case of iron, the interception of such species is further complicated by their high reactivity, which makes them incompatible with the reaction conditions commonly employed for the investigation of dinitrogen splitting reactions (photolysis, thermolysis).

We have shown above that the PNN scaffold herein described can accommodate tetrahedral geometries. Such geometries could, in principle stabilise better monometallic iron nitride intermediates compared to square planar environments.^[29]^ Moreover, as the redox‐active PNN scaffold facilitate electron shuttling processes, the possibility of an open‐shell iron nitride formation on a redox‐active ligand platform appeared intriguing.^[30]^ We envisaged that such species could be accessed by N‐atom transfer from lithium salts of 2,3 : 5,6‐Dibenzo‐7azabicyclo[2.2.1]hepta‐2,5‐diene (dbabh). These salts, first introduced by Mindiola and Cummins, have emerged as an attractive route for the synthesis of metal nitrides and provide a milder alternative to the well‐established photolytic pathways involving metal azides.^[31]^ In iron chemistry, Li(dbabh) reagents have been successfully employed by Peters in tripodal complexes with Fe−P linkages, where a diamagnetic iron(IV) nitride could be spectroscopically characterised.^[16a]^

**Scheme 3:**
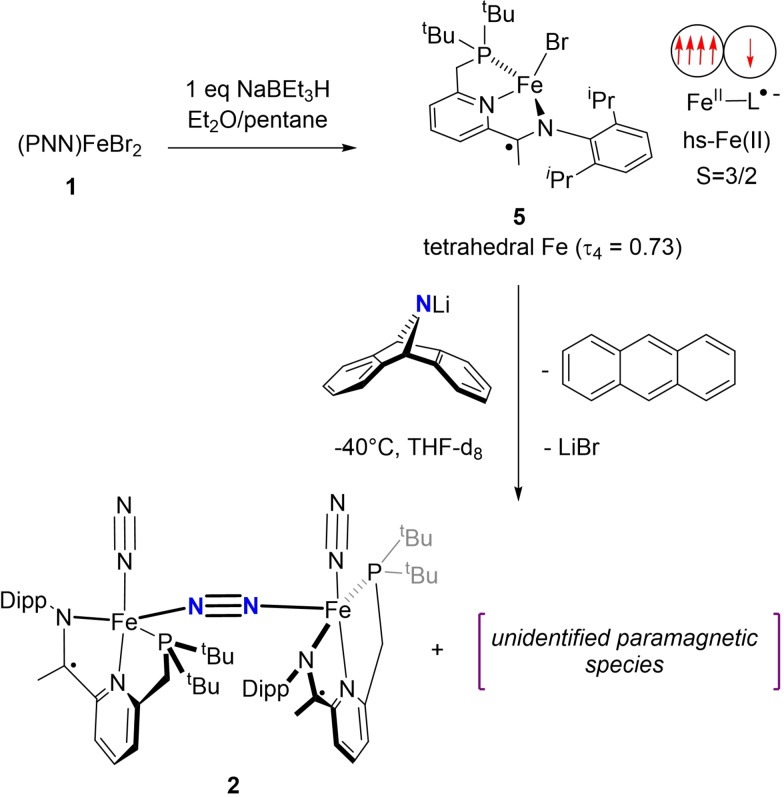
Reactivity of (PNN)FeBr (**5**) with Li(dbabh).

To set the stage for a salt metathesis reaction, the iron monohalide precursor (PNN)FeBr **5** was prepared by reducing **1** with one equivalent NaBEt_3_H (Scheme 3). The resulting paramagnetic species (S=^3^/_2_, μ_eff_=5.3(2)μ_B_, Evans’ method)^[32]^ exhibits a tetrahedral geometry at the iron centre (see the Supporting Information), differentiating it from pyridine‐ and pyrimidinediimine iron monohalide analogues, where the tridentate chelate is coplanar with the metal centre and the halide.^[33]^ Computational studies are consistent with a *hs*‐Fe(II) centre, antiferromagnetically coupled with a PNN ligand radical, therefore suggesting that the reduction reaction is ligand based.

Reaction of **5** with 1 equiv. Li(dbabh) at −60 °C in thf‐d_8_ under an N_2_ atmosphere, followed by direct subsequent inspection by NMR spectroscopy at −40 °C suggests rapid consumption of **5** within minutes alongside the formation of a new paramagnetic species.^[34]^ Maintaining the sample at −40 °C for longer yielded anthracene, alongside free PNN, [(PNN)Fe(N_2_)]_2_(μ‐N_2_) **2** and other paramagnetic species, as observed by ^1^H and ^31^P NMR spectroscopy. While the identity of these paramagnetic species is yet unclear, it is reasonable to propose the formation of a fleeting paramagnetic (PNN)Fe≡N.^[35]^ This can either dimerise to give **2** or can insert into the Fe−P bond which would account for the unidentified species. Similar decomposition pathways for iron nitrides have been previously reported.[^16a^, ^36^, ^37^] In contrast to the PNN system herewith described, the geometrically rigid (square planar) (PDI)FeCl reacts with Li(dbabh) to give stable amides, where no anthracene formation was reported under ambient or thermolytic conditions.[^38^, ^39^]

## Conclusion

We have shown that stepwise N_2_ extrusion of bridging/terminal (PNN)Fe complexes is accompanied by a spin change from low spin to high spin at the metal centre. Under vacuum, only the terminally bound N_2_ ligands in [(PNN)Fe(N_2_)]_2_(μ‐N_2_) dissociate, triggering a distortion from planarity in the (PNN)Fe fragment accompanied by a spin change at the iron centre from *ls*‐Fe(I) to *hs*‐Fe(I). These changes are facilitated by the conformational lability of the benzylic phosphine arm and the decreased overall π‐acidity of the PNN system. Nevertheless, because of metal‐metal and metal‐ligand antiferromagnetic coupling, the overall spin (S=0) is conserved during the dinitrogen dissociation reaction. The terminal dinitrogen dissociation enhances backbonding contributions from Fe to the bridging dinitrogen ligand, resulting in significant N=N activation. This remarkable activation triggers a bathochromic shift of the N=N stretching frequency by ca. 180 cm^−1^ and an increase of N=N separation by 0.04 Å. Moreover, this activation takes places despite the local spin change from *ls*‐Fe(I) to *hs*‐Fe(I). Prolonged heating or photolysis further triggers the dissociation of the bridging N_2_ ligand, resulting in a tetrahedral *hs*‐Fe(II) centre chelated by two iminopyridine ligands. While this clearly demonstrates that the formation of an iron nitride arising from N_2_ splitting is disfavoured, evidence for the formation of a transient nitride was independently obtained from salt metathesis of (PNN)FeBr and Li(dbabh) with the release of anthracene. Nevertheless, this species rapidly degrades even at −40 °C under N_2_ atmosphere to yield, among other products, the bridging dinitrogen species [(PNN)Fe(N_2_)]_2_(μ‐N_2_). Since the isolation of this putative iron nitride at this PNN platform may prove challenging, further studies are planned to increase the steric bulk on the ligand which may prevent other deleterious kinetically accessible pathways.

The present study reconfirms that spin state changes which govern iron‐mediated processes can be triggered by simple and sometimes overlooked reversible dinitrogen dissociation reactions. The dynamic nature of terminal N_2_ coordination can induce important geometrical reorganisation effects, which ultimately exert a significant effect on the degree of activation of coordinated bridging N_2_ even in the absence of strongly reducing agents.

## Experimental Section

[(^*tBu*^PNN)Fe(N_2_)]_2_(μ‐N_2_) (**2**) was prepared according to previously reported procedures.^[9]^

**Preparation of [(** ^***tBu***^ **PNN)Fe]_2_(μ‐N_2_) (3)**: In an argon filled glovebox [(^*tBu*^PNN)Fe(N_2_)]_2_(μ‐N_2_) (2) (150 mg, 140 μmol) was weighed into a vial and the solid was triturated repeatedly with hexane (6 times, 10 mL each) followed by removing the solvent *in vacuo* for 30–60 min. The title compound was obtained as a red‐brown solid (137 mg) with a maximum conversion of 83 % (^31^P NMR) with 17 % of starting material (**2**) as the only impurity. *Please note that even additional trituration cycles or longer times under high vacuum did not lead to a higher conversion but additional paramagnetic decomposition products started to form*. Crystals suitable for single crystal X‐ray diffraction were obtained from a concentrated solution in Et_2_O/Hexane (1 : 3) at −40 °C.

^**15**^ **N enriched sample preparation for Raman**: In an argon filled glovebox [(^*tBu*^PNN)Fe(N_2_)]_2_(μ‐N_2_) (**2**) (100 mg) was dissolved in Et_2_O (5.00 mL). A schlenk flask fitted with a septum pierced with the cannula of a ^15^N_2_ gas container was evacuated and backfilled with ^15^N_2_ (1 atm). The solution of **2** was filtered into the Schlenk flask, stirred for 10 min and the solvent removed *in vacuo*. The compound was further triturated with hexane (4x 5.00 mL) and the solvent removed *in vacuo* for 30 min to give the ^15^N enriched title compound. ^1^H NMR (600 MHz, C_6_D_6_, 295 K) δ[ppm]=8.04 (t, *J=*6.7 Hz, ^*4*^ *J_PH_* =6.2 Hz, 2H, H5), 7.32 (m, 2H, H14), 7.23 (d, *J=*7.6 Hz, 4H, H13), 6.97 (t, *J*=7.5 Hz, 2H, H6), 6.77 (d, *J*=7.9 Hz, 2H, H7), 4.17 (d, ^*2*^ *J_PH_* =3.1 Hz, 4H, H3), 3.36 (sept, *J*=6.8 Hz, 4H, H15), 1.44 (d, *J*=6.8 Hz, 12H, H16/17), 1.25 (d, ^*3*^ *J_PH_* =10.8 Hz, 36H, H1), 0.98 (s, 6H, H10), 0.87 (d, *J*=6.8 Hz, 12H, H16/17). ^13^C{^1^H} NMR (151 MHz, C_6_D_6_, 295 K) δ[ppm]=166.1 (d, ^*2*^ *J_PC_* =15.5 Hz, C_q_, C4), 153.9 (s, C_q_, C11), 147.0 (s, C_q_, C8), 144.6 (s, C_q_, C9), 141.3 (s, C_q_, C12), 126.5 (s, CH, C13/14), 123.5 (s, CH, C13/14), 122.3 (d, ^*4*^ *J_PC_* =1.6 Hz, CH, C7), 121.7 (d, ^*4*^ *J_PC_* =1.6 Hz, CH, C6), 116.3 (d, ^*3*^ *J_PC_* =32.6 Hz, CH, C5), 34.7 (d, ^*1*^ *J_PC_* =20.1 Hz, CH_2_, C3), 32.2 (d, ^*1*^ *J_PC_* =23.9 Hz, C_q_, C2), 30.3 (d, ^*2*^ *J_PC_* =14.0 Hz, CH_3_, C1), 27.9 (s, CH, C15), 25.0 (s, CH_3_, C16/17), 24.2 (s, CH_3_, C16/17), 19.2 (s, CH_3_, C10). ^31^P{^1^H} NMR (243 MHz, C_6_D_6_, 295 K) δ[ppm]=28.7. Raman (^14^N_2_‐2) ν[cm^−1^]=1778 (ν bridging N_2_). (^15^N_2_‐2) ν [cm^−1^]=1720 (ν bridging N_2_). Mössbauer (80 K): δ=0.62 mm s^−1^, |ΔE_Q_|=1.69 mm s^−1^ (96 %), residual starting material (2, 4 %): δ=0.40 mm s^−1^, |ΔE_Q_|=1.05 mm s^−1^.

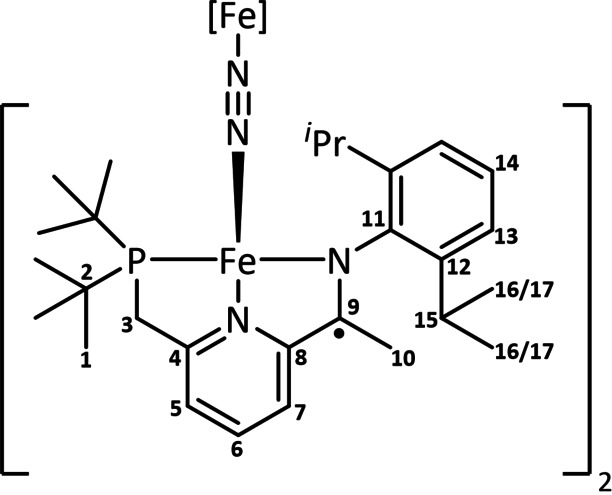




Spectroscopic and analytical measurements: ^1^H, ^13^C{^1^H}, ^31^P{^1^H} spectra were recorded using a Bruker Avance VIII‐400 or Bruker Avance III HD 600 MHz spectrometer. ^1^H NMR spectra (400.1 MHz or 600.1 MHz) were referenced to the residual protons of the deuterated solvent used. ^13^C{^1^H} NMR spectra were referenced internally to the D‐coupled ^13^C resonances of the NMR solvent. ATR‐IR (solid state) measurements were performed in a nitrogen filled glovebox (SylaTech Y05G) using an Agilent Cary 630 FTIR spectrometer equipped with a diamond ATR unit. Raman spectra of powdered samples (for a detailed preparation procedure see Supporting Information) were collected with a Renishaw inVia Reflex confocal Raman microscope in backscattering configuration equipped with a 50× long working distance objective (N.A. 0.5). Excitation was performed with a 532 nm laser diode at an excitation density of 0.692 mW cm^−2^ using an integration time of 10 s and 10 accumulations. To minimize the influence of spot‐to‐spot variation spectra were measured at least at 20 different spots and averaged for each sample. Elemental Analyses were carried out on an Elementar vario MICRO cube in the Microanalysis Laboratory of the Heidelberg Chemistry Department. ^57^Fe Mössbauer data were recorded on spectrometers with alternating constant acceleration. The minimum experimental line width was 0.24 mm/s (full width at half‐height) and the source was ^57^Co/Rh. The sample temperature was maintained constant either in an Oxford Instruments Variox cryostat or in a Wissel MBBC‐HE0106 bath cryostat. Isomer shifts are quoted relative to iron metal at room temperature. Simulations were performed with the JulX Software developed by Dr. Eckhard Bill at the Max‐Planck‐Institut für Chemische Energiekonversion.

Crystallographic Data: Deposition Number(s) 2167510 (for **3**), 2167511 (for **4**), 2167512 (for **5**) contain(s) the supplementary crystallographic data for this paper. These data are provided free of charge by the joint Cambridge Crystallographic Data Centre and Fachinformationszentrum Karlsruhe Access Structures service.

### Computational Methods

All density functional theory (DFT) calculations were performed using the ORCA quantum chemical program package (Version 4.2.1).^[40]^ Geometry optimizations of the complexes **2**–**5** were performed using the corresponding crystal structures, without any truncation of their structures, as starting geometries. Geometry optimizations of all complexes were undertaken by employing the hybrid‐GGA (GGA=generalized gradient approximation) density functional B3LYP,^[41]^ in conjunction with Ahlrichs triple‐zeta def2‐TZVP basis set^[42]^ and the appropriate auxiliary basis set (def2/J).^[43]^ For **2**–**4** a basis set combination was used: def2‐TZVP(‐f) on Fe and the coordinating atoms (**2** and **3**: on N and P atoms; **4**: N atoms) and def2‐SVP on all other atoms. To speed up the overall calculations, the RIJCOSX^[44]^ approximation was applied for the expensive integral calculations. Noncovalent interactions were accounted for by using atom‐pairwise dispersion corrections with Becke‐Johnson damping (D3BJ).^[45]^ Solvent effects were accounted for using the Conductor‐like Polarizable Continuum Model (C‐PCM)^[46]^ with the dielectric constant of benzene. Subsequent numerical frequency calculations were undertaken for the optimized geometries to confirm they correspond to stationary points featuring no imaginary frequencies. To account for the basis set superposition error (BSSE) the geometrical Counterpoise correction (gCP)^[47]^ as implemented in ORCA was used. To ensure the match of basis sets, single point calculations for **2**, **3** and N_2_ employing the def2‐TZVP basis set on all atoms as well as the gCP(DFT/TZ) keyword were used.

**Broken‐Symmetry Calculations**: The broken symmetry (BS) formalism^[48]^ was employed in unrestricted calculations to check for antiferromagnetic coupling of two spins. BS calculations were performed for all complexes using the B3LYP functional and the same basis set (def2‐TZVP or def2‐SVP//def2‐TZVP) as mentioned earlier. In each case, multiple fragments were defined: PNN, Fe, N_2_ and Br^−^. Because several BS solutions of the spin‐unrestricted Kohn ‐ Sham equations may be obtained, the general notation BS(*m,n*) was used, where *m (n)* denotes the number of spin‐up (spin‐down) electrons at the iron centre (*m*) or the PNN ligand (*n*). For the dimeric complexes **2** and **3** the notation BS(n_1_, m_1_, m_2_, n_2_) was used, where the indices stand for the iron‐PNN subunits, which are connected through a bridging N_2_ ligand. The spin multiplicity for the broken symmetry calculations were chosen according to the high spin state ‐ for example triplet for BS(1,1), quintet for BS(2,2), etc.

^**31**^ **P NMR Calculations**: The NMR shifts were calculated from the averaged isotropic chemical shielding σ of the P atoms using the pcSseg‐2^[49]^ basis set in combination with the AutoAux^[50]^ procedure employing the TPSS0 functional. As a reference the experimental ^31^P NMR shift of PMe_3_ [δ_exp_(PMe_3_)] was measured in C_6_D_6_ and the isotropic chemical shielding calculated. The theoretical chemical shift of the molecule was determined as: δ_calc_=δ_exp_(PMe_3_)+(σ_PMe3_−σ_molecule_).

**Mössbauer Calculations**: To compute Mössbauer parameters, single‐point DFT calculations were performed for the geometry optimized structures using the B3LYP density functional in conjunction with the core properties basis set CP(PPP)^[51]^ on Fe, def2‐TZVP basis set, for all other atoms. The RIJCOSX approximation was *not* applied. The isomer shifts (δ) were computed from the electron densities ρ_0_ at the Fe nuclei using the linear equation: δ=α ⋅ (ρ_0_‐C)+β. C is a constant, and α and β are the fitting parameters. Their values were obtained from previously reported DFT calibration work (B3LYP, α=−0.366, β=2.852, C=11810).^[52]^ The quadrupole splitting parameter ΔE_Q_ was obtained from the electric field gradients V_*ij*_.

Input file examples for all types of calculations can be found in the Supporting Information.

## Conflict of interest

The authors declare no conflict of interest.

1

## Supporting information

As a service to our authors and readers, this journal provides supporting information supplied by the authors. Such materials are peer reviewed and may be re‐organized for online delivery, but are not copy‐edited or typeset. Technical support issues arising from supporting information (other than missing files) should be addressed to the authors.

Supporting Information

## Data Availability

The data that support the findings of this study are available from the corresponding author upon reasonable request.
