# Supplementary material for: Terminal N2 Dissociation in [(PNN)Fe(N2)]2(μ‐N2) Leads to Local Spin‐State Changes and Augmented Bridging N2 Activation
Source: Chemistry. 2022 Aug 18;28(58):e202202172. doi: 10.1002/chem.202202172 (PMC9804668; doi:10.1002/chem.202202172)
Supplement: Supplementary file 1 — Supporting Information [file CHEM-28-0-s001.pdf]

Chemistry–A European Journal

Supporting Information

Terminal N_2 Dissociation in $[(PNN)Fe(N_2)]_2(\mu\text{-}N_2)$ Leads to Local Spin-State Changes and Augmented Bridging N_2 Activation

Nicolas I. Regenauer, Hubert Wadepohl, and Dragoş-Adrian Roşca*

Table of Contents

General **S2**

Preparation of Metal Complexes **S4**

Supporting Crystallographic Information **S6**

NMR Spectra **S10**

Reaction of (PNN)FeBr (5) with Li(dbabh) **S13**

Calculation of Singlet-Triplet Gaps and Exchange Constants by NMR Spectroscopy **S16**

IR Spectra **S20**

Raman Spectra **S21**

UV-Vis Spectra **S25**

Computational Details **S26**

XYZ Coordinates of Optimized Structures **S35**

General. Unless otherwise stated, all manipulations were performed using standard Schlenk techniques under dry nitrogen or argon atmosphere in flame-dried glassware or in an nitrogen or argon filled MBraun glovebox. All sovlents described in the procedures were dried before use, unless stated otherwise. Anhydrous solvents were freshly distilled from appropriate drying agents (CH_2Cl_2 over CaH_2, THF over Na/benzophenone, EtOH over Na) or dried over activated alumina columns (M. Braun SPS 800 - Et_2O, hexane, toluene, methanol) and were transferred under Argon. $[(^{tBu}PNN)Fe(N_2)]_2(\mu\text{-}N_2)$ (**2**) was prepared according to the previously reported procedure.[1]

NMR spectroscopy. ^1H, $^{13}C\{^1H\}$, $^{31}P\{^1H\}$ spectra were recorded using a Bruker Avance VIII-400 or Bruker Avance III HD 600 MHz spectrometer. Deuterated solvents were distilled from the appropriate drying agents, degassed by three freeze-pump-thaw cycles and stored over 4 Å molecular sieves prior to use. ^1H NMR spectra (400.1 MHz or 600.1 MHz) were referenced to the residual protons of the deuterated solvent used. $^{13}C\{^1H\}$ NMR spectra were referenced internally to the D-coupled ^{13}C resonances of the NMR solvent. Where appropriate, resonances were assigned using 2D NMR homo- and heterocorrelation (COSY, HMBC, HSQC) techniques. Chemical shifts (δ) are given in ppm, relative to TMS, coupling constants (J) in Hz. For NMR signals of paramagnetic species the Hertz values in parentheses correspond to the FWHM values as determined using Topspin Version *3.5 pl7*.

IR: ATR (solid state) measurements were performed in a nitrogen filled glovebox (SylaTech Y05G) using an Agilent Cary 630 FTIR spectrometer equipped with a diamond ATR unit.

Raman: Powdered samples on aluminium foil were encapsulated between two microscope slides and sealed with superglue (more details on **page S22**, example see **Figure S24**). Raman spectra of encapsulated samples were collected with a Renishaw inVia Reflex confocal Raman microscope in backscattering configuration equipped with a 50× long working distance objective (N.A. 0.5). Excitation was performed with a 532 nm laser diode at an excitation density of 0.692 mW cm^{-2} using an integration time of 10 s and 10 accumulations. To minimize the influence of spot-to-spot variation spectra were measured at least at 20 different spots and averaged for each sample.

UV-Vis absorption spectra were recorded under an argon atmosphere on a Cary 5000UV/Vis-spectrometer and were base line and solvent corrected.

Elemental Analyses were carried out on an Elementar vario MICRO cube in the Microanalysis Laboratory of the Heidelberg Chemistry Department.

[1] N. I. Regenauer, H. Wadepohl, D.-A. Roșca, *Inorg. Chem.* **2022**, *61*, 7426–7435.

^{57}Fe Mößbauer data were recorded on spectrometers with alternating constant acceleration. The minimum experimental line width was 0.24 mm/s (full width at half-height) and the source was ^{57}Co/Rh. The sample temperature was maintained constant either in an Oxford Instruments Variox cryostat or in a Wissel MBBC-HE0106 bath cryostat. Isomer shifts are quoted relative to iron metal at room temperature. Simulations were performed with the JulX Software developed by Dr. Eckhard Bill at the Max-Planck-Institut für Chemische Energiekonversion.

Preparation of Metal Complexes

Additional characterization data for [(^{tBu}PNN)Fe(N_2)]$_2$(μ-N_2) (2): IR (ATR) $\tilde{\nu}$ [cm^{-1}] = 2060 (ν_{sym} terminal N_2), 2039 (ν_{asym} terminal N_2), 1959 (ν bridging N_2). **Raman** $\tilde{\nu}$ [cm^{-1}] = 1959 (ν bridging N_2). **Mößbauer (80 K):** δ = 0.40 mm s^{-1}, |ΔE_Q| = 1.05 mm s^{-1} (100 %).

[(^{tBu}PNN)Fe]$_2$(μ-N_2) (3). In an argon filled glovebox [(^{tBu}PNN)Fe(N_2)]$_2$(μ-N_2) (**2**) (150 mg, 140 µmol) was weighed into a vial and the solid was triturated repeatedly with hexane (6 times, 10 mL each) followed by removing the solvent *in vacuo* for 30–60 min. The title compound was obtained as a red-brown solid (137 mg) with a maximum conversion of 83 % (^{31}P NMR) with 17 % of starting material (**2**) as the only impurity. *Please note that even additional trituration cycles or longer times under high vacuum did not lead to a higher conversion but additional paramagnetic decomposition products started to form.* Crystals suitable for single crystal X-ray diffraction were obtained from a concentrated solution in Et_2O/Hexane (1:3) at -40 °C. *^{15}N enriched sample preparation for Raman:* In an argon filled glovebox [(^{tBu}PNN)Fe(N_2)]$_2$(μ-N_2) (**2**) (100 mg) was dissolved in Et_2O (5.00 mL). A schlenk flask fitted with a septum pierced with the cannula of a $^{15}N_2$ gas container was evacuated and backfilled with $^{15}N_2$ (1 atm). The solution of **2** was filtered into the Schlenk flask, stirred for 10 min and the solvent removed *in vacuo*. The compound was further triturated with hexane (4x 5.00 mL) and the solvent removed *in vacuo* for 30 min to give the ^{15}N enriched title compound.

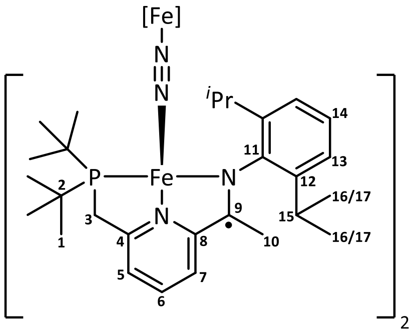

^{1}H NMR (600 MHz, C_6D_6, 295 K) δ[ppm] = 8.04 (t, *J* = 6.7 Hz, $^4J_{PH}$ = 6.2 Hz, 2H, H5), 7.32 (m, 2H, H14), 7.23 (d, *J* = 7.6 Hz, 4H, H13), 6.97 (t, *J* =7.5 Hz, 2H, H6), 6.77 (d, *J* =7.9 Hz, 2H, H7), 4.17 (d, $^2J_{PH}$ = 3.1 Hz, 4H, H3), 3.36 (sept, *J* = 6.8 Hz, 4H, H15), 1.44 (d, *J* = 6.8 Hz, 12H, H16/17), 1.25 (d, $^3J_{PH}$ = 10.8 Hz, 36H, H1), 0.98 (s, 6H, H10), 0.87 (d, *J* = 6.8 Hz, 12H, H16/17). **^{13}C{^{1}H} NMR** (151 MHz, C_6D_6, 295 K) δ[ppm] = 166.1 (d, $^2J_{PC}$ = 15.5 Hz, C_q, C4), 153.9 (s, C_q, C11), 147.0 (s, C_q, C8), 144.6 (s, C_q, C9), 141.3 (s, C_q, C12), 126.5 (s, CH, C13/14), 123.5 (s, CH, C13/14), 122.3 (d, $^4J_{PC}$ = 1.6 Hz, CH, C7), 121.7 (d, $^4J_{PC}$ = 1.6 Hz, CH, C6), 116.3 (d, $^3J_{PC}$ = 32.6 Hz, CH, C5), 34.7 (d, $^1J_{PC}$ = 20.1 Hz, CH_2, C3), 32.2 (d, $^1J_{PC}$ = 23.9 Hz, C_q, C2), 30.3 (d, $^2J_{PC}$ = 14.0 Hz, CH_3, C1), 27.9 (s, CH, C15), 25.0 (s, CH_3, C16/17), 24.2 (s, CH_3, C16/17), 19.2 (s, CH_3, C10). **^{31}P{^{1}H} NMR** (243 MHz, C_6D_6, 295 K) δ[ppm] = 28.7. **Raman** (**$^{14}N_2$-2**) $\tilde{\nu}$ [cm^{-1}] = 1778 (ν bridging N_2). (**$^{15}N_2$-2**) $\tilde{\nu}$ [cm^{-1}] = 1720 (ν bridging N_2). **UV-Vis:** λ_{max} [nm] = 337 (hexane); 339 (toluene). **Mößbauer (80 K):** δ = 0.62 mm s^{-1}, |ΔE_Q| = 1.69 mm s^{-1} (96 %), residual starting material (**2**, 4 %): δ = 0.40 mm s^{-1}, |ΔE_Q| = 1.05 mm s^{-1}.

(^{tBu}PNN)$_2$Fe (4). <u>*NMR scale:*</u> In an argon filled glovebox [(^{tBu}PNN)Fe]$_2$(μ-N$_2$) (**3**) (20 mg, 19.7 μmol) was dissolved in C_6D_6 (600 μL) and filled into a J. Young NMR tube. The mixture was heated at 80 °C for 7 h. After this time the ^{31}P{^{1}H} NMR of the reaction mixture showed full conversion of the starting material. <u>*Preparative scale:*</u> In a nitrogen filled glovebox [(^{tBu}PNN)Fe(N$_2$)]$_2$(μ-N$_2$) (**2**) (50 mg, 46.6 μmol) was dissolved in C_6D_6 (1.00 mL) and filled into a J. Young NMR tube. The mixture was heated in an oil bath at 85 °C for 11 h. During the reaction the color changed from dark green to chocolate brown. After filtration over Celite the solvent was removed *in vacuo*. The solid residue was dissolved in pentane (4.00 mL), filtered into a vial and placed at -40 °C. The mother liquor was decanted and the crystalline solid was dried *in vacuo.* The title compound was obtained as a brown solid (3.8 mg, 17 %). Recrystallization from Et$_2$O at -40 °C afforded crystals suitable for single crystal X-ray diffraction. *Please note that this reaction can be carried out at different temperatures: at 80 °C in benzene the reaction takes around 7 h, at 100 °C in toluene it takes only 30 min.*

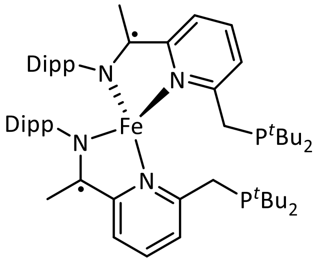

^{1}H NMR (600 MHz, C_6D_6, 295 K) δ[ppm] = 207.16 (319 Hz), 114.69 (98 Hz), 108.20 (166 Hz), 17.07 (63 Hz), 1.11 (m), -2.15 (36 Hz), -3.24 (22 Hz), -5.68 (25 Hz), -8.93 (28 Hz), -12.13 (46 Hz), -12.75 (26 Hz), -19.59 (423 Hz), -29.23 (160 Hz), -53.22 (732 Hz), -56.06 (467 Hz), -324.14 (427 Hz). **^{31}P{^{1}H} NMR** (243 MHz, C_6D_6, 295 K) δ[ppm] = -28.2 (104 Hz). **Magnetic susceptibility (Evans, C_6D_6, 295 K)** μ_{eff} [μ_B] = 3.4(2). **EA:** Anal. Calcd. for $C_{56}H_{86}FeN_4P_2$ [%]: C 72.08, H 9.29, N 6.00; found: C 71.96, H 9.46, N 6.09.

(^{tBu}PNN)FeBr (5). In a nitrogen filled glovebox (^{tBu}PNN)FeBr$_2$ (**1**) (100 mg, 153 μmol, 1.0 eq.) was weighed into a vial and suspended in benzene (4.00 mL). NaBEt$_3$H (1 M in toluene, 150 μL, 150 μmol, 0.98 eq.) was added dropwise and the mixture was stirred for 30 min at r.t. The initial blue suspension turns forest green. The mixture was filtered through a PTFE-syringe filter and the solvent removed *in vacuo*. The title compound was obtained as a forest green solid (83.9 mg, 95 %). Slow evaporation of a concentrated solution of **5** in Et$_2$O at r.t. afforded crystals suitable for single crystal X-ray diffraction.

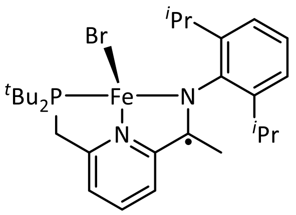

^{1}H NMR (600 MHz, C_6D_6, 295 K) δ[ppm] = 291.37 (622 Hz), 192.86 (218 Hz), 139.68 (302 Hz), 10.36 (377 Hz), -2.24 (60 Hz), -14.30 (41 Hz), -24.61 (39 Hz), -35.63 (207 Hz), -55.45 (1097 Hz), -408.81 (600 Hz). **Magnetic susceptibility (Evans, C_6D_6, 295 K)** μ_{eff} [μ_B] = 5.3(2). **EA:** Anal. Calcd. for $C_{28}H_{43}BrFeN_2P$ [%]: C 58.55, H 7.55, N 4.88; found: C 60.01, H 7.88, N 4.81.

Supporting Crystallographic Information

X-ray Crystal Structure Determinations

Crystal data and details of the structure determinations are compiled in Table S1. Full shells of intensity data were collected at low temperature with an Agilent Technologies Supernova-E CCD diffractometer (Mo- or Cu-K_α radiation, microfocus X-ray tubes, multilayer mirror optics). Detector frames (typically ω-, occasionally φ-scans, scan width 0.4...1°) were integrated by profile fitting.[2,3] Data were corrected for air and detector absorption, Lorentz and polarization effects[3] and scaled essentially by application of appropriate spherical harmonic functions.[3,4,5] Absorption by the crystal was treated numerically (Gaussian grid).[4,6] An illumination correction was performed as part of the numerical absorption correction.[4]

The structures were solved by ab initio dual space methods involving difference Fourier syntheses (VLD procedure, compound **4**)[7] or by the charge flip procedure (compounds **3** and **5**)[8] and refined by full-matrix least squares methods based on F^2 against all unique reflections.[9] All non-hydrogen atoms were given anisotropic displacement parameters. When found necessary, suitable adp restraints were applied.[10,11] Hydrogen atoms were input at calculated positions and refined with a riding model.[11]

---

[2] K. Kabsch, in: M. G. Rossmann, E. Arnold (eds.) *"International Tables for Crystallography" Vol. F*, Ch. 11.3, Kluwer Academic Publishers, Dordrecht, The Netherlands, **2001**.
[3] *CrysAlisPro*, Agilent Technologies UK Ltd., Oxford, England, UK, **2011-2014** and Rigaku Oxford Diffraction, Rigaku Polska Sp.z o.o., Wrocław, Poland, **2015-2021**.
[4] *SCALE3 ABSPACK, CrysAlisPro*, Agilent Technologies UK Ltd., Oxford, England, UK, **2011-2014** and Rigaku Oxford Diffraction, Rigaku Polska Sp.z o.o., Wrocław, Poland, **2015-2021**.
[5] R. H. Blessing, *Acta Cryst.* **1995**, *A51*, 33.
[6] W. R. Busing, H. A. Levy, *Acta Cryst.* **1957**, *10*, 180.
[7] (a) M. C. Burla, R. Caliandro, B. Carrozzini, G. L. Cascarano, C. Cuocci, C. Giacovazzo, M. Mallamo, A. Mazzone, G. Polidori, D. Siliqi *SIR2019*, CNR IC, Bari, Italy, **2019**; (b) M. C. Burla, R. Caliandro, B. Carrozzini, G. L. Cascarano, C. Cuocci, C. Giacovazzo, M. Mallamo, A. Mazzone, G. Polidori, *J. Appl. Cryst.* **2015**, *48*, 306.
[8] (a) L. Palatinus, *SUPERFLIP*, EPF Lausanne, Switzerland and Fyzikální ústav AV ČR, v. v. i., Prague, Czech Republic, **2007-2014**; (b) L. Palatinus, G. Chapuis, *J. Appl. Cryst.* **2007**, 40, 786; (c) L. Palatinus, *Acta. Cryst.* **2013**, *B69*, 1.
[9] (a) G. M. Sheldrick, *SHELXL-20xx*, University of Göttingen and Bruker AXS GmbH, Karlsruhe, Germany, **2012-2018**; (b) W. Robinson, G. M. Sheldrick in: N. W. Isaaks, M. R. Taylor (eds.) „*Crystallographic Computing 4*", Ch. 22, IUCr and Oxford University Press, Oxford, England, UK, **1988**; (c) G. M. Sheldrick, *Acta Cryst.* **2008**, *A64*, 112; (d) G. M. Sheldrick, *Acta Cryst.* **2015**, *C71*, 3.
[10] A. Thorn, B. Dittrich, G. M. Sheldrick, *Acta Cryst.* **2012**, *A68*, 448.
[11] (a) J. S. Rollett in: F. R. Ahmed, S. R. Hall, C. P. Huber (eds.) „*Crystallographic Computing*" p. 167, Munksgaard, Copenhagen, Denmark, **1970**; (b) D. Watkin in: N. W. Isaaks, M. R. Taylor (eds.) „*Crystallographic Computing 4*", Ch. 8, IUCr and Oxford University Press, Oxford, England, UK, **1988**; (c) P. Müller, R. Herbst-Irmer, A. L. Spek, T. R. Schneider, M. R. Sawaya in: P. Müller (ed.) *"Crystal Structure Refinement"*, Ch. 5, Oxford University Press, Oxford, England, UK, **2006**; (d) D. Watkin, *J. Appl. Cryst.* **2008**, *41*, 491.

CCDC 2167510 - 2167512 contains the supplementary crystallographic data for this paper. These data can be obtained free of charge from the Cambridge Crystallographic Data Centre's and FIZ Karlsruhe's joint Access Service via https://www.ccdc.cam.ac.uk/structures/?.

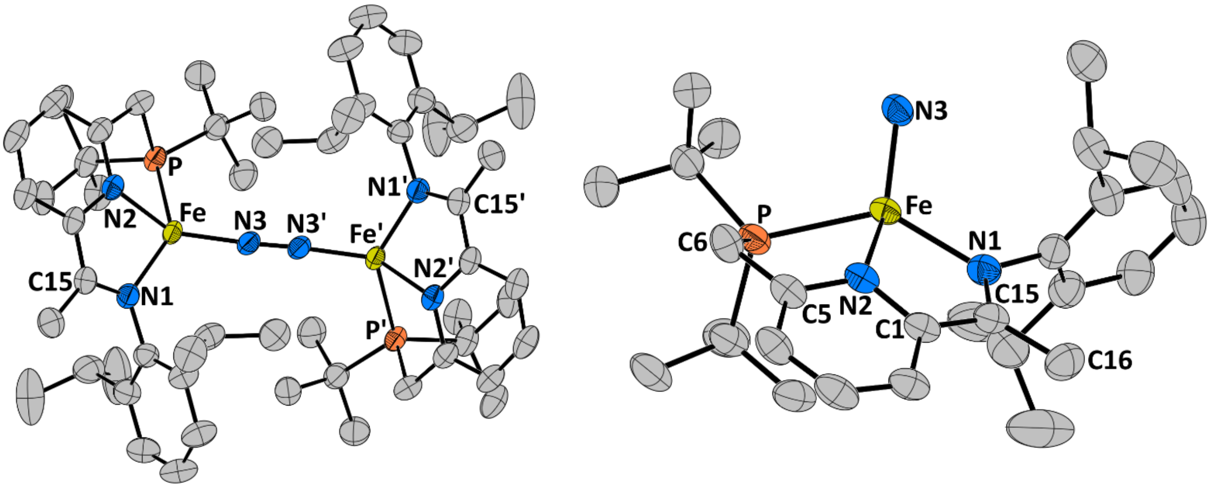

Figure S 1. Molecular structure (left) and asymmetric unit (right) of **3** with displacement ellipsoids drawn at 50% probability. Hydrogen atoms are omitted for clarity. Selected bond distances [Å]: Fe—P 2.3309(6), Fe—N1 1.9707(18), Fe—N2 1.9901(17), Fe—N3 1.7799(17), N3—N3' 1.175(3), N1—C15 1.349(3), P—C6 1.872(2), C5—C6 1.500(3), N2—C5 1.367(3), N2—C1 1.385(3), C1—C15 1.408(3), C15—C16 1.499(3). Selected angles [°]: N3'—N3—Fe 176.5(2), N3—Fe—P 105.87(6), N3—Fe—N2 139.21(8), N3—Fe—N1 114.25(7), P–Fe-N1 136.89(5).

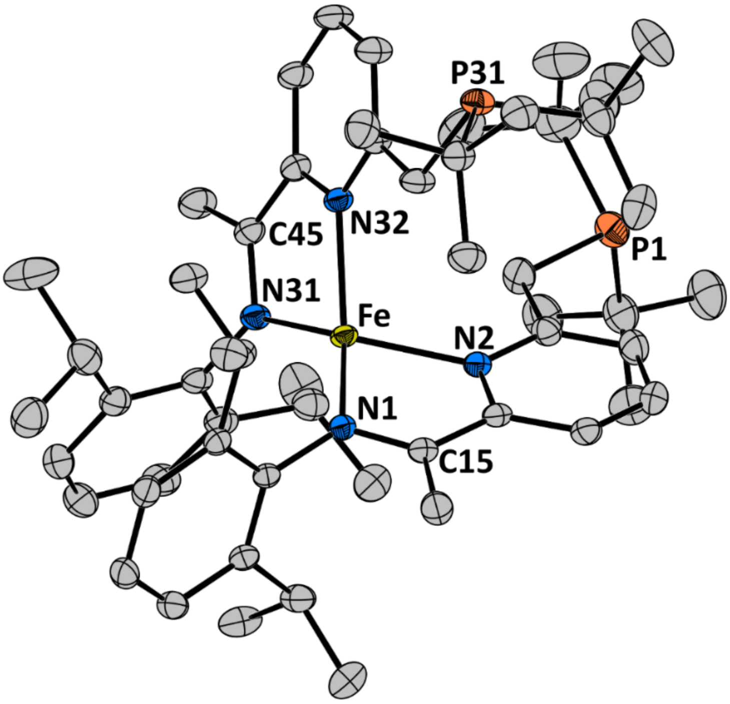

Figure S 2. Molecular structure of **4** with displacement ellipsoids drawn at 50% probability. Hydrogen atoms are omitted for clarity. Selected bond distances [Å]: Fe–N1 1.9959(13), Fe–N2 2.0515(13), Fe–N31 1.9994(14), Fe–N32 2.0522(13), N1–C15 1.345(2), N31–C45 1.349(2). Selected angles [°]: N1–Fe–N2 80.99(5), N31–Fe–N32 81.29(5), N32–Fe–N1 131.75(6), N32–Fe–N2 104.29(5), N31–Fe–N1 128.28(6), N31–Fe–N2 135.32(6).

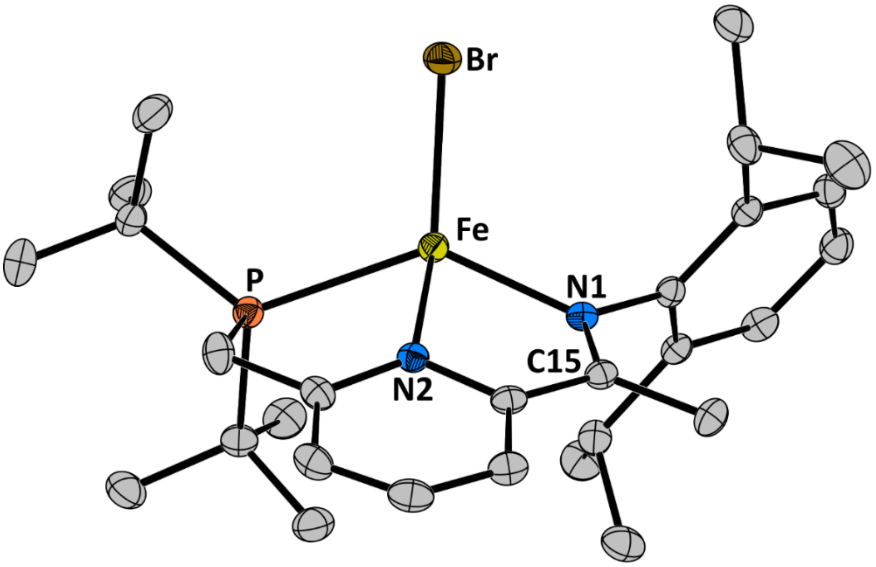

Figure S 3. Molecular structure of **5** with displacement ellipsoids drawn at 50% probability. Hydrogen atoms are omitted for clarity. Selected bond distances [Å]: Fe–P 2.4723(7), Fe–N1 2.0262(19), Fe–N2 2.0198(19), Fe–Br 2.3731(4), N1–C15 1.345(3). Selected angles [°]: Br–Fe–P 113.40(2), Br–Fe–N2 122.76(6), Br–Fe–N1 111.62(6), P–Fe–N1 134.91(6).

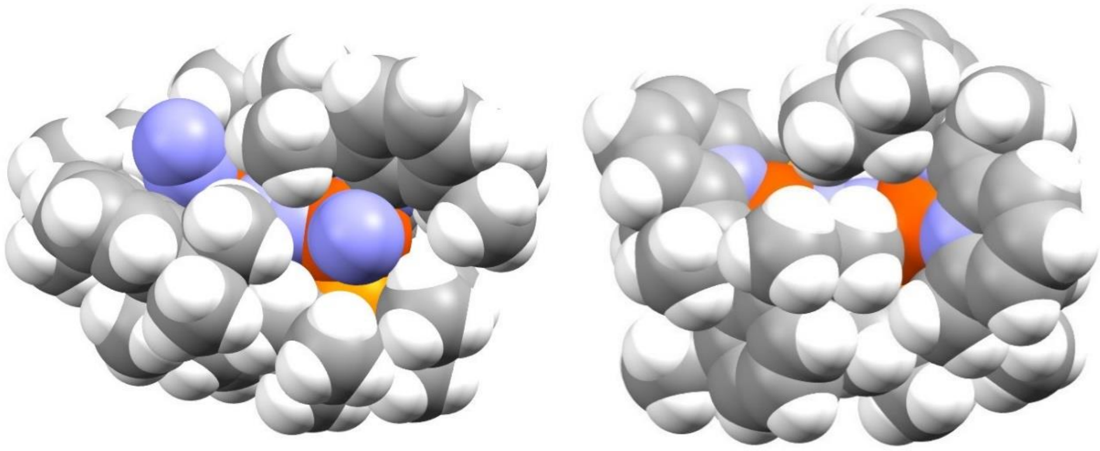

Figure S 4. Space filling plots for **2** (left) and **3** (right). The bridging N_2 ligand is efficiently shielded as a result of geometry reorganisation in **3**.

Table S 1. Details of crystal structure determinations of **3**, **4**, and **5**.

	**3**	**4**	**5**
formula	$C_{56}H_{86}Fe_2N_6P_2$	$C_{56}H_{86}FeN_4P_2$	$C_{28}H_{43}BrFeN_2P$
crystal system	orthorhombic	triclinic	monoclinic
space group	*P bcn*	*P* -1	*P* $2_1/n$
a /Å	15.7190(3)	9.51435(15)	14.7847(6)
b /Å	17.6200(4)	12.8935(2)	11.8008(4)
c /Å	20.2175(6)	22.5557(4)	16.8435(7)
α /°		90.3062(15)	
β /°		93.4872(13)	108.554(4)
γ /°		98.3446(14)	
V /Å^3	5599.6(2)	2732.35(8)	2786.0(2)
Z	4	2	4
M_r	1016.94	933.07	574.37
F_{000}	2184	1012	1204
d_c /Mg·m^{-3}	1.206	1.134	1.369
μ /mm^{-1}	4.992	3.041	2.051
max., min. transmission factors	0.923, 0.686	1.000, 0.612	1.000, 0.953
X-radiation, λ /Å	Cu K_α, 1.54184	Cu K_α, 1.54184	Mo K_α, 0.71073
data collect. temperat. /K	120(1)	120(1)	120(1)
θ range /°	3.8 to 70.6	3.5 to 70.6	2.4 to 32.6
index ranges *h,k,l*	-19 ... 19, -21 ... 21, -23 ... 24	-11 ... 11, -15 ... 15, -27 ... 27	-22 ... 22, -17 ... 17, -25 ... 25
reflections measured	107423	65371	69077
unique [R_{int}]	5365 [0.072]	10348 [0.066]	10156 [0.126]
observed [$I\geq2\sigma(I)$]	4353	9526	6588
data / restraints /parameters	5365 / 18 / 309	10348 / 0 / 590	10156 / 0 / 309
GooF on F^2	1.016	1.029	1.011
R indices [$F>4\sigma(F)$] $R(F)$, $wR(F^2)$	0.0361, 0.0889	0.0378, 0.0982	0.0516, 0.0866
R indices (all data) $R(F)$, $wR(F^2)$	0.0496, 0.0960	0.0420, 0.1016	0.0990, 0.1018
largest residual peaks /e·Å^{-3}	0.223, -0.254	0.458, -0.380	0.877, -0.661
**CCDC deposition number**	**2167510**	**2167511**	**2167512**

NMR Spectra

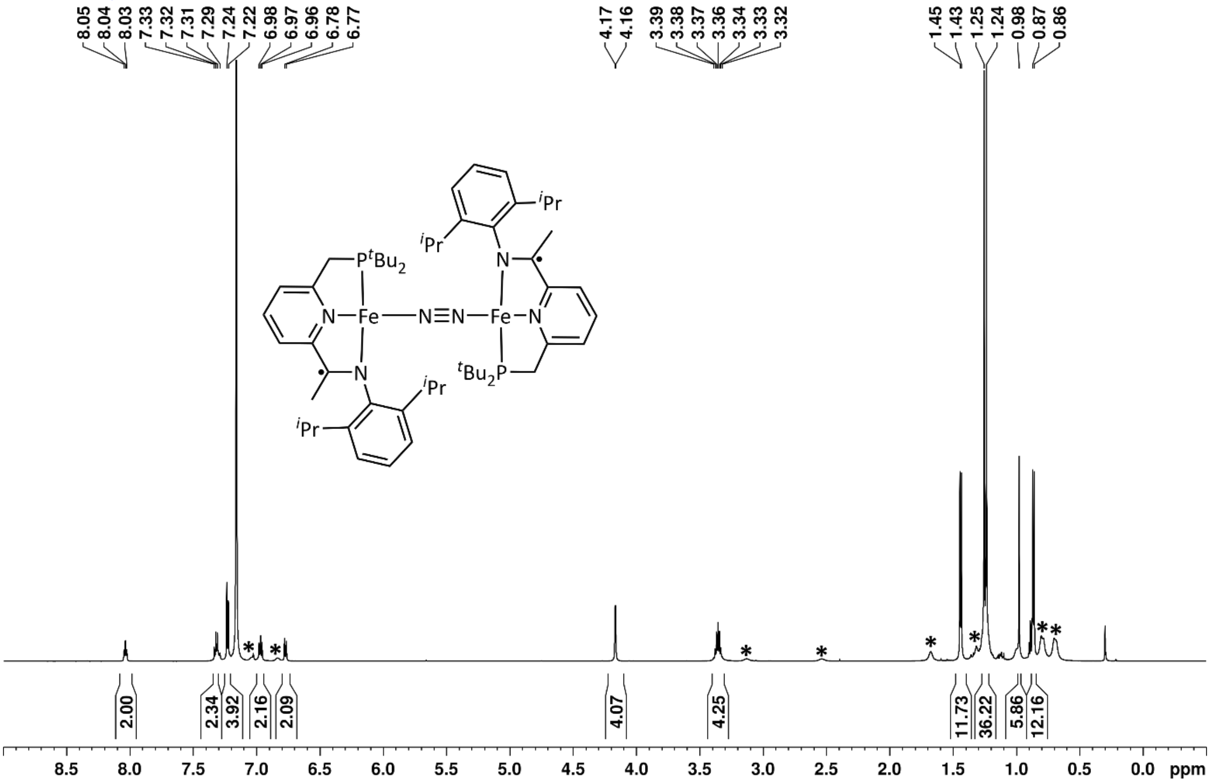

Figure S 5. ^{1}H NMR spectrum of **3** (600 MHz, C_6D_6, 295 K). Additional signals marked with an asterisk (*) correspond to residual starting material (**2**).

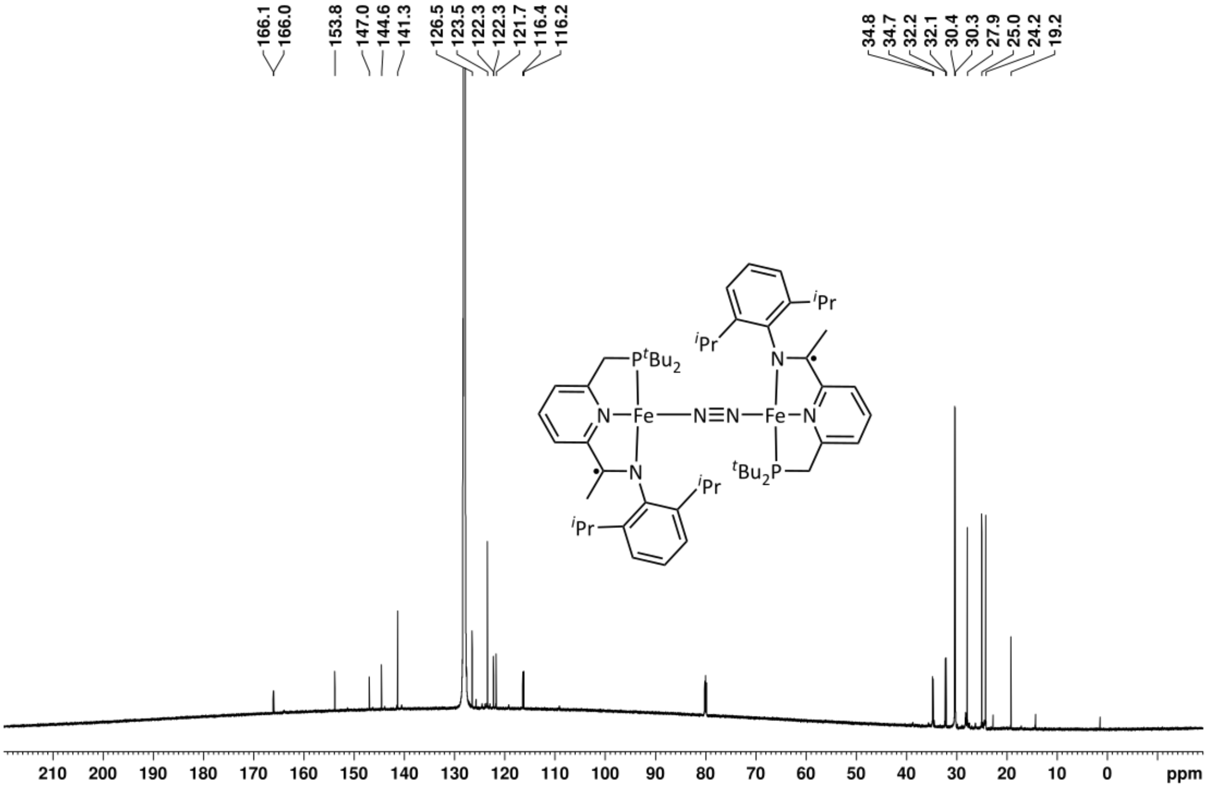

Figure S 6. $^{13}C\{^{1}H\}$ NMR spectrum of **3** (151 MHz, C_6D_6, 295 K).

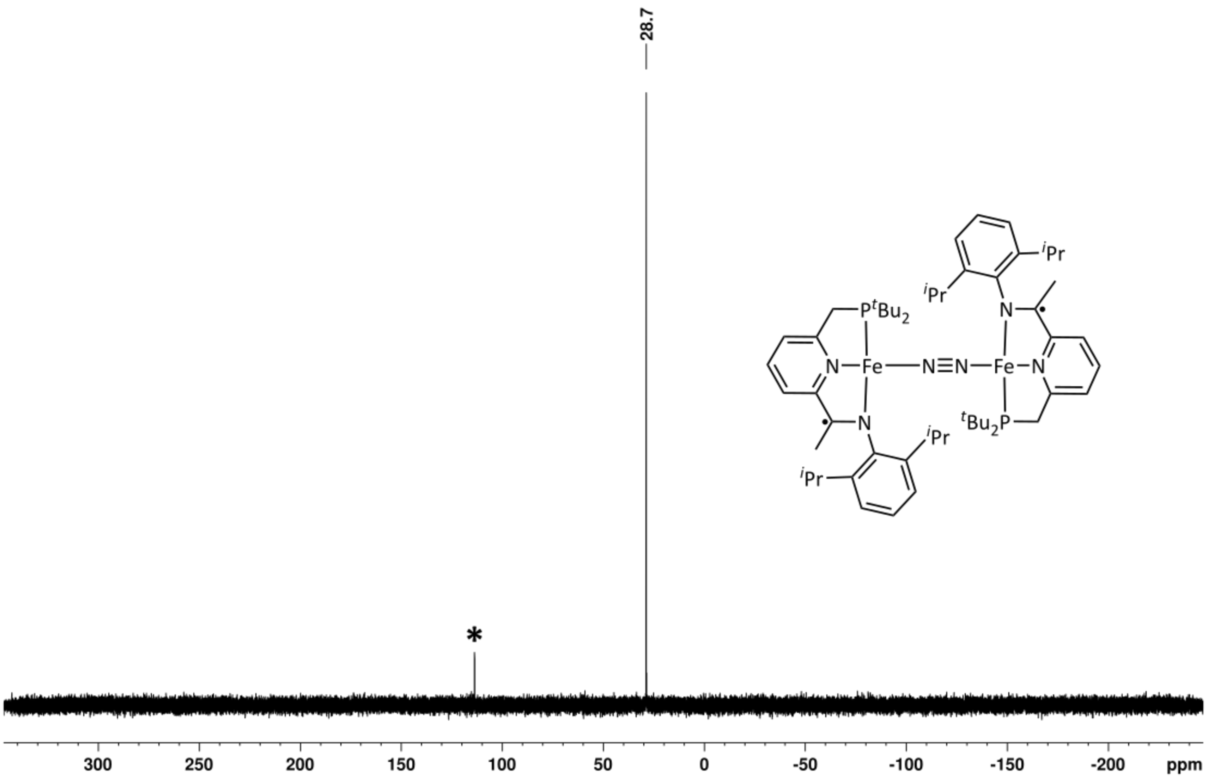

Figure S 7. $^{31}P\{^{1}H\}$ NMR spectrum of **3** (243 MHz, C_6D_6, 295 K). The signal marked with an asterisk (*) corresponds to residual starting material (**2**).

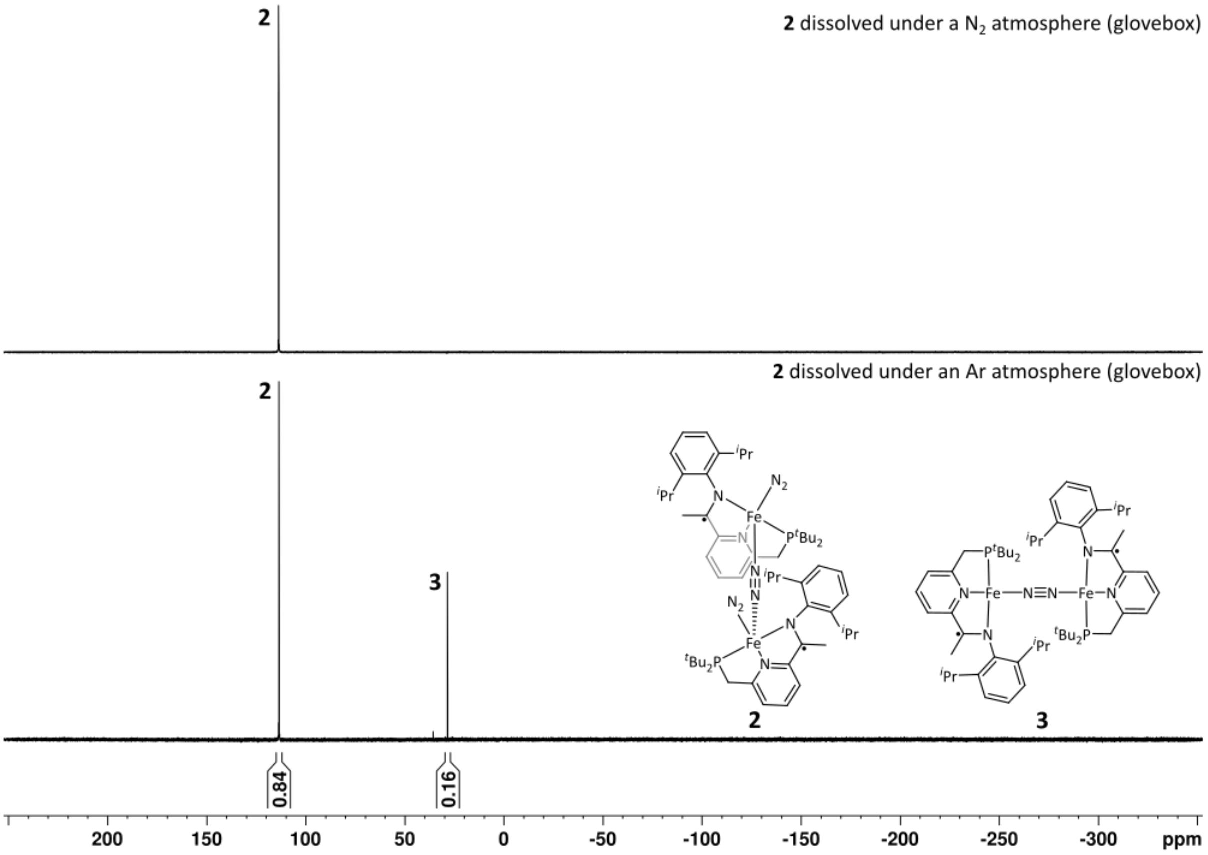

Figure S 8. $^{31}P\{^{1}H\}$ NMR (243 MHz, C_6D_6, 295 K) of **2** dissolved under N_2 atmosphere (top) and **2** dissolved under Ar atmosphere (bottom) resulting in the formation of ca. 16 % of **3**.

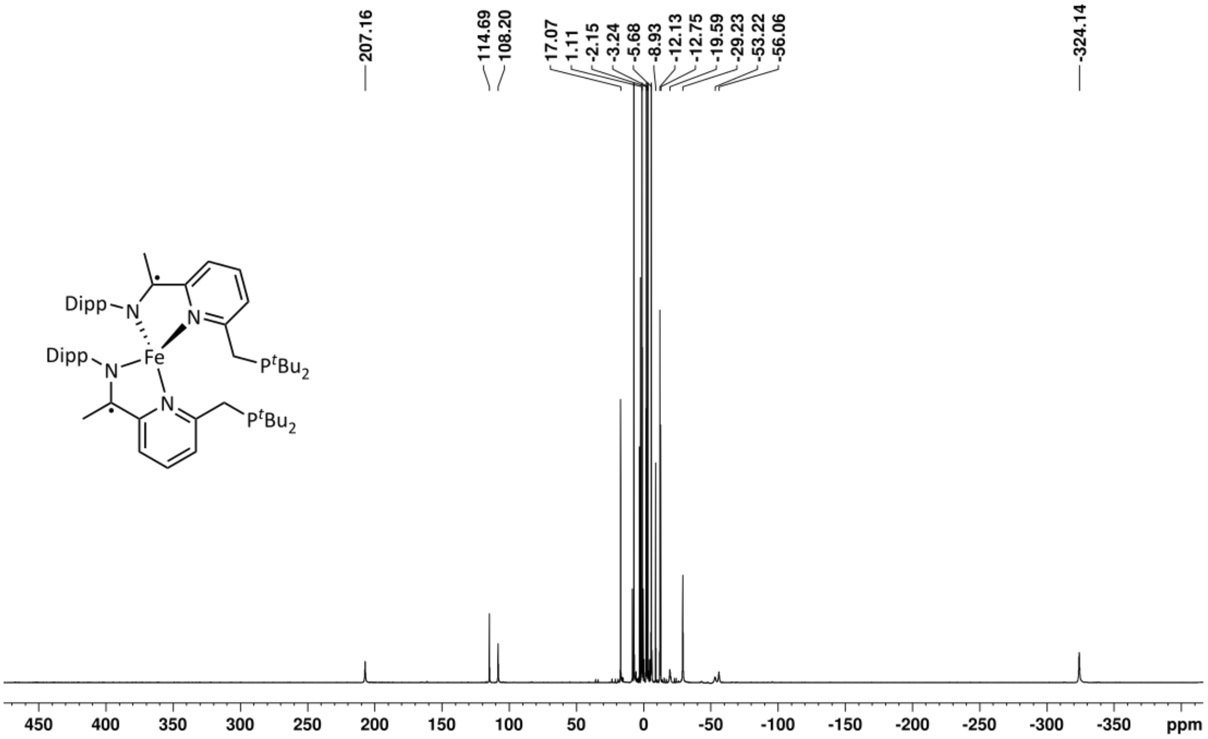

Figure S 9. ^{1}H NMR spectrum of **4** (600 MHz, C_6D_6, 295 K).

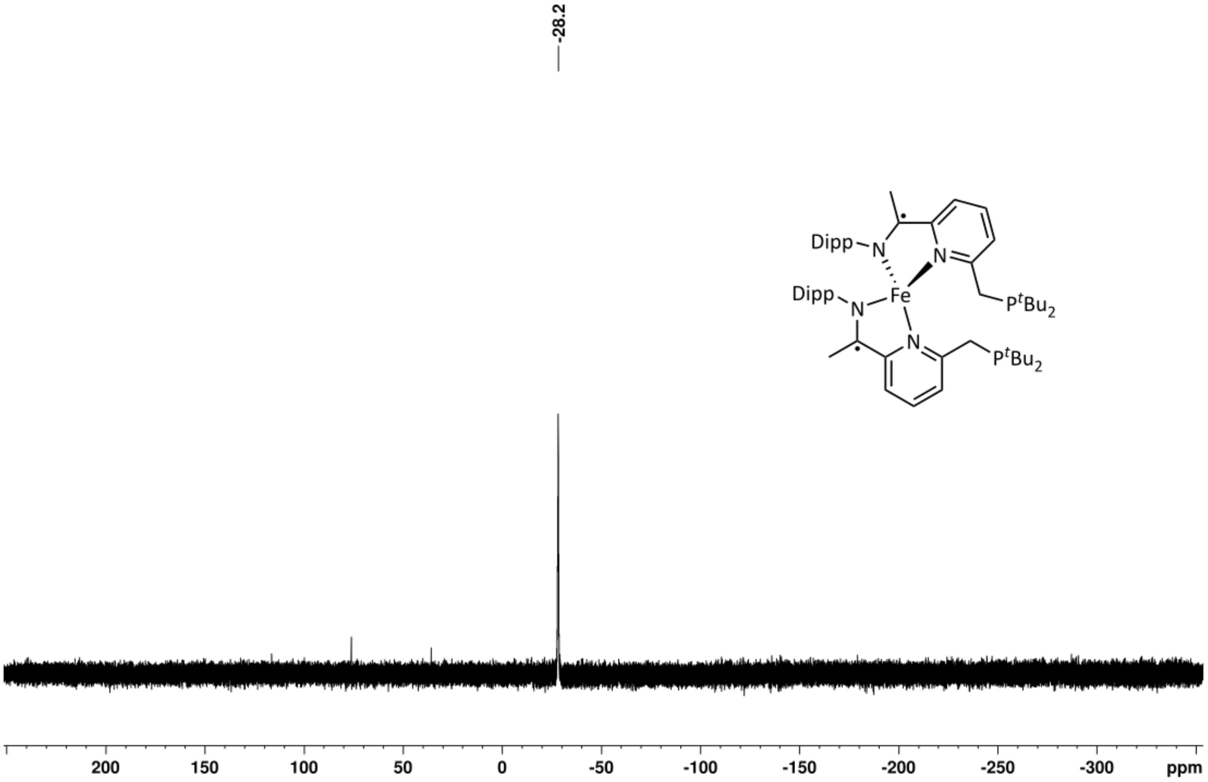

Figure S 10. ^{31}P{^{1}H} NMR spectrum of **4** (243 MHz, C_6D_6, 295 K).

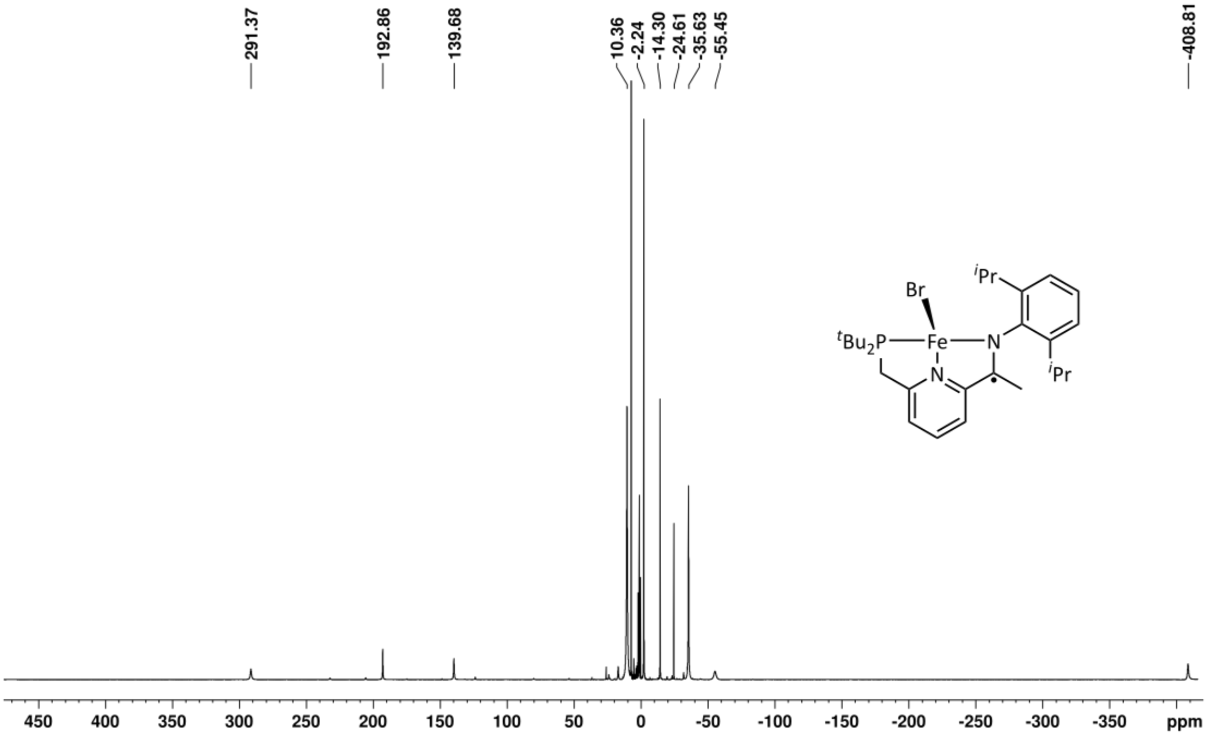

Figure S 11. ^{1}H NMR spectrum of **5** (600 MHz, C_6D_6, 295 K).

NMR Experiments

In the cold-well of a N_2 filled glovebox cooled to -60°C, a J-Young NMR tube charged with a precooled solution of (PNN)FeBr **5** (15 mg, 26 µmol) in THF-d_8 (400 µL) was treated with a pre-cooled solution of Li(dbabh) (7.2 mg, 26 µmol) in THF-d_8 (300 µL). The solution was transferred into the probe-head of an NMR machine, pre-cooled to -40°C. The consumption of (PNN)FeBr was immediately observed, concomitant with the formation of anthracene, [(PNN)Fe(N_2)](µ-N_2) **2**, small amounts of free PNN ligand and other unidentified paramagnetic species. The formation of [(PNN)Fe(N_2)](µ-N_2) **2** was further confirmed by ^{31}P{^{1}H} NMR spectroscopy (Figure S14). Storing the reaction mixture at -40°C afforded subsequent accumulation of anthracene and **2**.

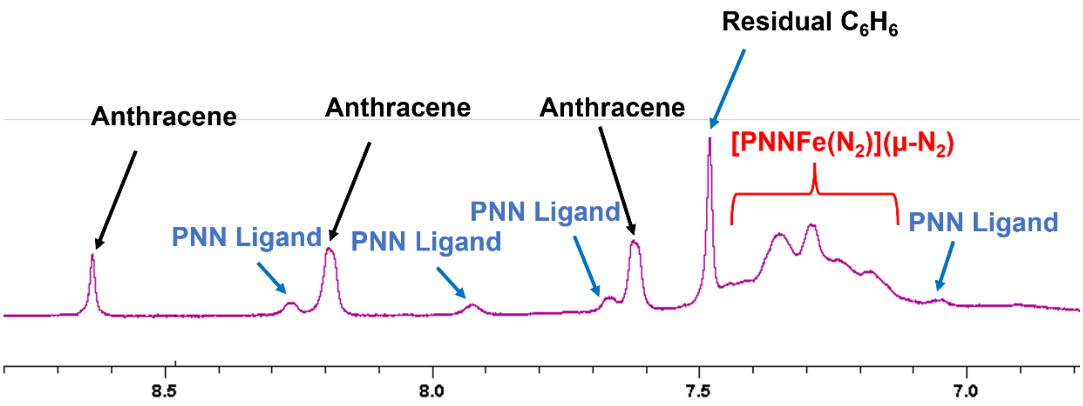

Figure S 12. Aromatic region of the ^1H NMR spectrum (600 MHz, THF-d_8, -40°C) of the reaction between **5** and Li(dbabh) immediately after the addition.

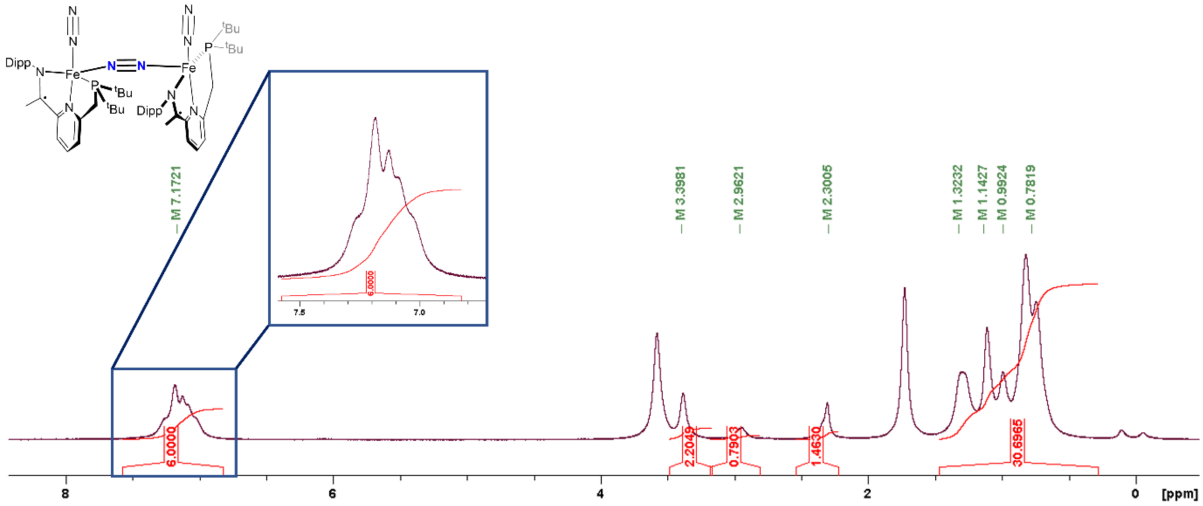

Figure S 13. ^1H NMR spectrum (600 MHz, THF-d_8, -40°C) of an authentic sample of **2**, prepared by reducing (PNN)$FeBr_2$ with $NaBEt_3H$ (2 eq.).

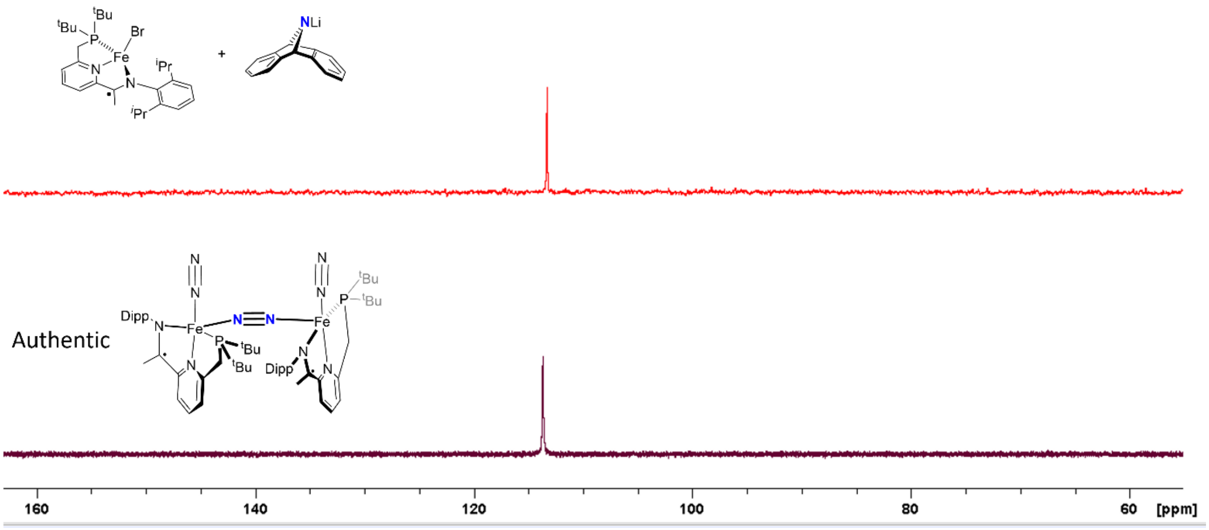

Figure S 14. Comparison of (a) the ^{31}P NMR spectrum (THF-d$_8$, -40°C) of the reaction products of the reaction of **5** and Li(dbabh) (red) and (b) the ^{31}P NMR spectrum (THF-d$_8$, -40°C) of an independently prepared sample of **2** (violet).

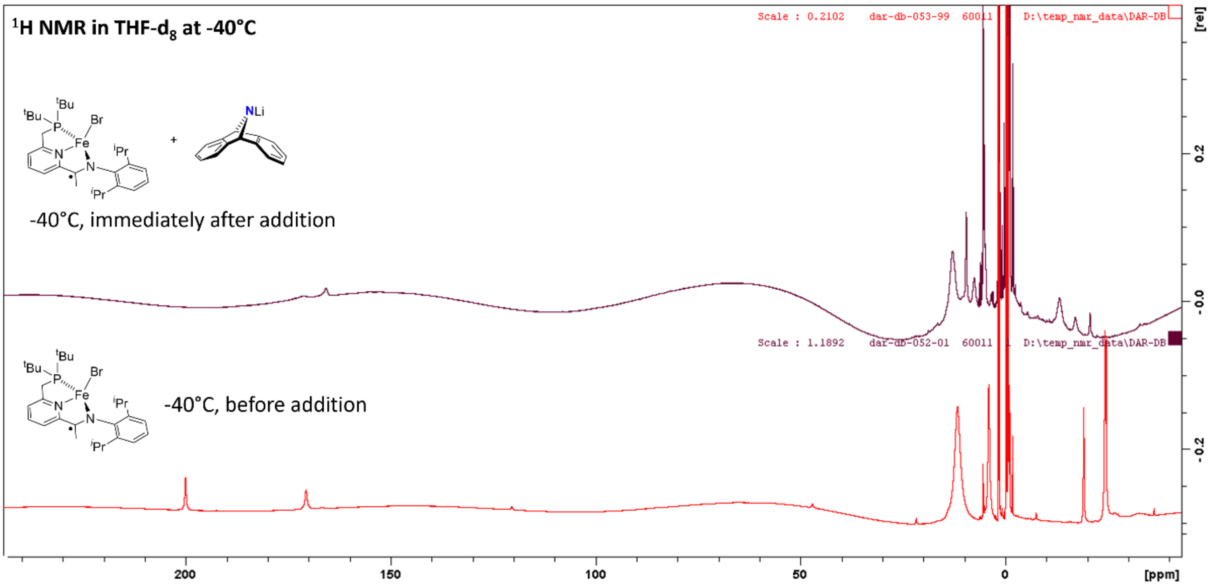

Figure S 15. Comparison of the ^{1}H NMR spectra recorded for (PNN)FeBr **5** (THF-d$_8$, -40°C) (bottom) and the reaction products formed immediately after the addition of Li(dbabh) to **5** (THF-d$_8$, -40°C) (top).

Calculation of Singlet-Triplet Gaps and Exchange Constants by NMR Spectroscopy

Both ^{1}H and ^{31}P could be used to investigate the magnetic properties of **2** by NMR spectroscopy in a temperature window of 150°C (between +70 to -80°C). The ^{31}P chemical shift was clearly observable with minimal line broadening over this temperature range. For the 1H NMR signals, out of all the resonances, only the m-py resonance (δ_H[22°C] = 8.04) and the benzylic CH_2 resonance (δ_H[22°C] = 4.17) could be used to extract magnetic data. In the case of the other resonances, significant line broadening and overlap prevented the accurate monitoring of chemical shifts as a function of temperature.

For the calculation of the exchange constant, the following equation[12] derived from the Bleaney-Bowers equation, was fitted to a plot of δ_P or δ_H vs. 1/T:

$$\delta = \delta_0 + B \cdot \frac{1}{T} \cdot \frac{[\exp\frac{2J}{kT} + 5\exp\frac{6J}{kT}]}{[1 + 3\exp\frac{2J}{kT} + 5\exp\frac{6J}{kT}]}$$

where δ_0 is the chemical shift of ^{31}P in the ground state, B is a fitting constant and 2J and 6J are the exchange constants corresponding to the energy difference between the singlet-triplet and singlet quintet states respectively, consistent with the notation $H_{HDVV} = -2J\widehat{S_1} \cdot \widehat{S_2}$ for the Heisenberg-Dirac-van Vleck Hamiltonian.[13]

The fit of the ^{31}P data yielded the following parameters with a confidence interval of 3σ (99.7%)

$$\delta_0 = 18.7 \pm 0.3\,ppm$$

$$B = 138198.0 \pm 11016$$

$$J = -4607.8 \pm 150\,Joule$$

The δ_0 value deviates only slightly from the value measured at -80°C (21.1 ppm). The fit yields an exchange constant ***J* = -385(1) cm^{-1}** which corresponds to a **singlet-triplet gap of 2.21(2) kcal mol^{-1}.**

The fit of the ^{1}H NMR data of the m-py resonance (δ_H[22°C] = 8.04) yielded the following parameters with a confidence interval of 3σ (99.7%):

$$\delta_0 = 8.052 \pm 0.01\,ppm$$

$$B = -3432.75 \pm 112$$

$$J = -3978.93 \pm 75\,Joule$$

[12] S. Pfirrmann, C. Limberg, C. Herwig, C. Knispel, B. Braun, E. Bill and R. Stösser, *J. Am. Chem. Soc.* **2010**, *132*, 13684-13691.

[13] For similar approaches, see: (a) J. Rittle, C. C. McCrory and J. C. Peters, *J. Am. Chem. Soc.* **2014**, *136*, 13853-13862; (b) A. W. Tepper, L. Bubacco and G. W. Canters, *Chem. Eur. J.* **2006**, *12*, 7668-7675; (c) M.-E. Zaballa, L. Ziegler, D. J. Kosman and A. J. Vila, *J. Am. Chem. Soc.* **2010**, *132*, 11191-11196; (d) D. J. Schild and J. C. Peters, *ACS Catal* **2019**, *9*, 4286-4295; (e) T. J. Steiman and C. Uyeda, *J. Am. Chem. Soc.* **2015**, *137*, 6104-6110; (e) P. W. Smith and T. D. Tilley, *J. Am. Chem. Soc.* **2018**, *140*, 3880-3883.

The δ_0 value deviates only slightly from the value measured at -80°C (7.93 ppm). The fit yields an exchange constant ***J* = -332(1) cm^{-1}** which corresponds to a **singlet-triplet gap of 1.9(2) kcal mol^{-1}.**

The fit of the ^{1}H NMR data of the benzylic resonance (δ_H[22°C] = 4.17) yielded the following parameters with a confidence interval of 3σ (99.7%):

$$\delta_0 = 3.752 \pm 0.005\, ppm$$

$$B = 1477.41 \pm 58$$

$$J = -3972.09 \pm 91\, Joule$$

The δ_0 value deviates only slightly from the value measured at -80°C (3.81 ppm). The fit yields an exchange constant ***J* = -332(1) cm^{-1}** which corresponds to a **singlet-triplet gap of 1.9(2) kcal mol^{-1}.**

While we note that the differences in the J values obtained from ^{1}H and ^{31}P NMR spectroscopy are very small (-385(1) cm^{-1} *vs.* -332(1) cm^{-1}), we chose to present the ^{31}P data in the main article, as the ^{1}H resonances were considerably broadened at high (above 40°C) and low (below -40°C) temperature.

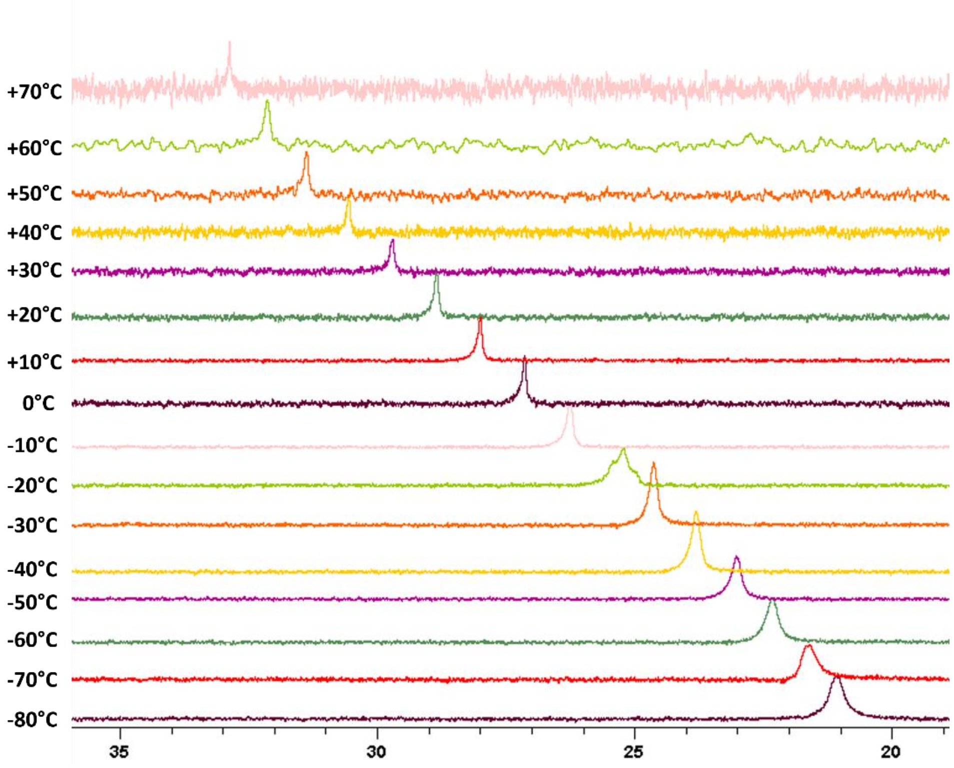

Figure S 16. ^{31}P{^{1}H} NMR spectra (400 MHz, toluene-d$_8$) of **3** at variable temperatures (between -80 °C and +70 °C) showing the temperature dependence of the phosphorous shift.

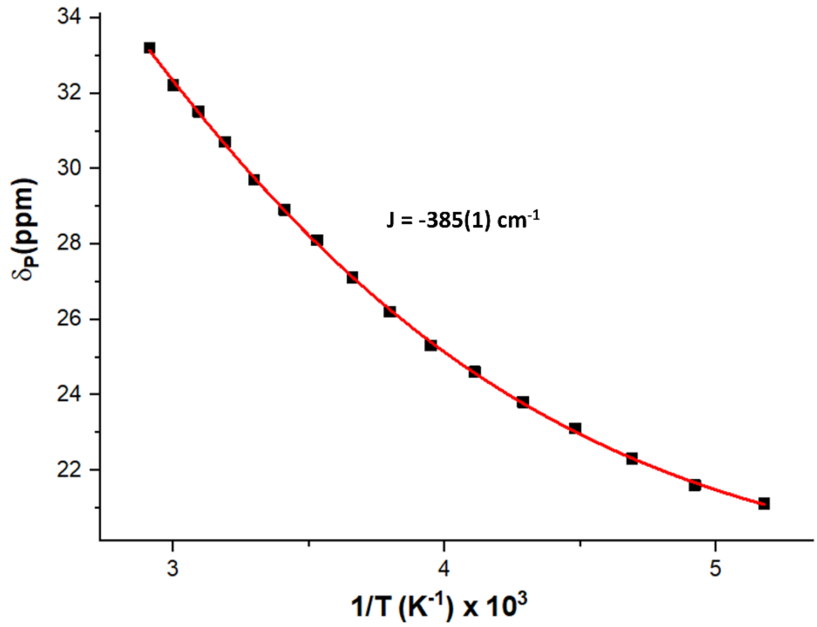

Figure S 17. Plot of δ_P (ppm) vs 1000/T (K^{-1}) of compound **3** indicating non-Curie behaviour. A fit using the Boltzmann function for a singlet-triplet (based on the notation $\boldsymbol{\widehat{H}_{HD\nu V} = -2J\widehat{S_1} \cdot \widehat{S_2}}$ obtained from the Heisenberg-Dirac-van Vleck Hamiltonian).

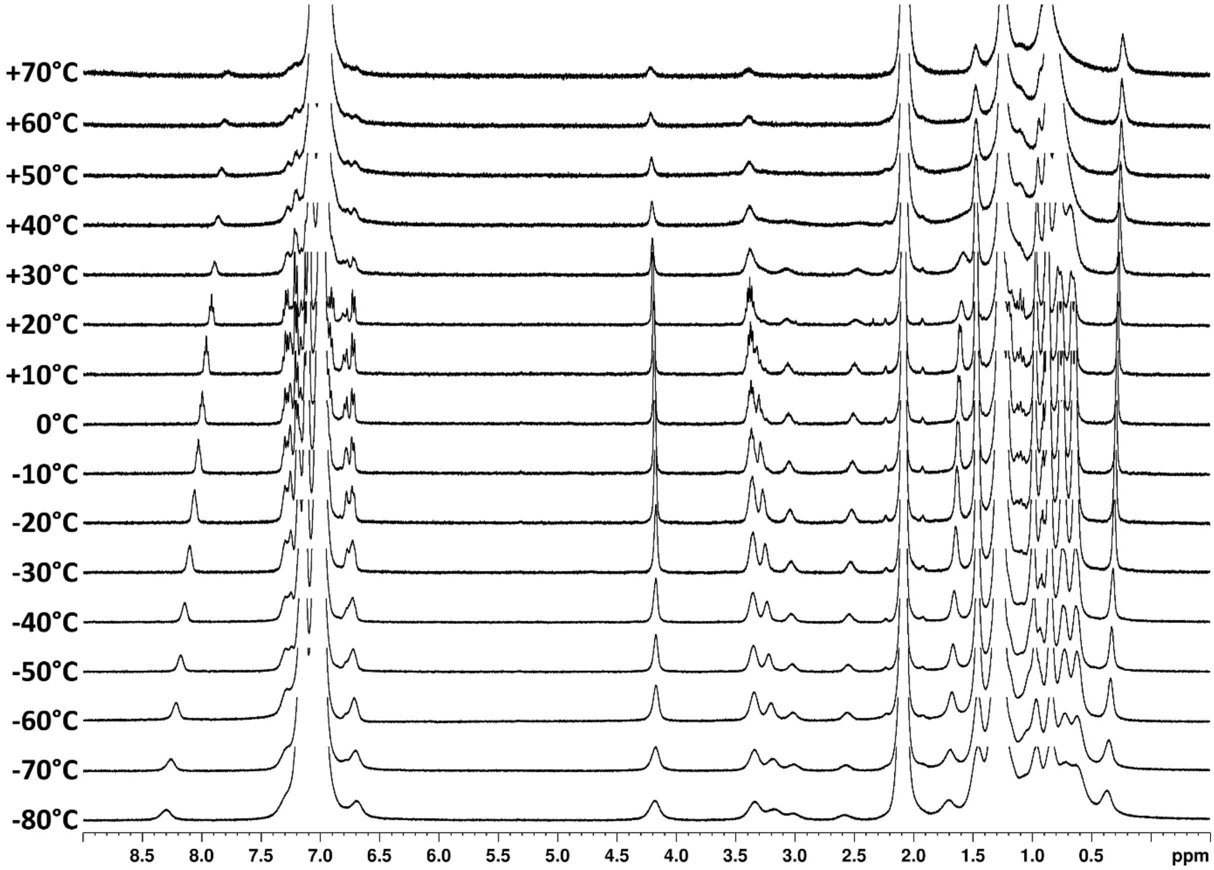

Figure S 18. ^{1}H NMR spectra (400 MHz, toluene-d$_8$, referenced to PhC*H*D$_2$ at 2.08 ppm) of **3** at variable temperatures (between -80 °C and +70 °C) showing the temperature dependence of the chemical shifts.

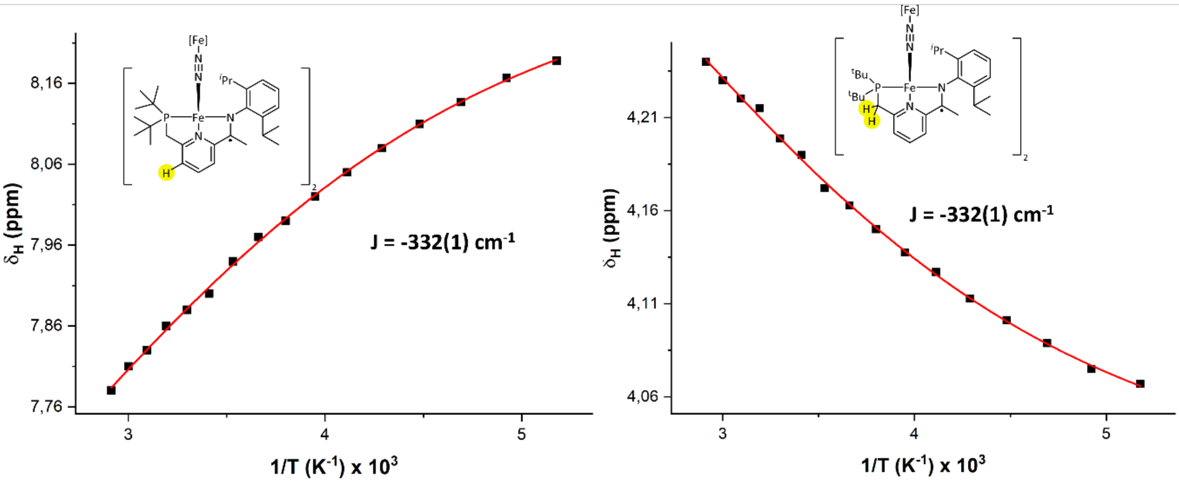

Figure S 19. Plots of δ_H (ppm) vs 1000/T (K^{-1}) of compound **3** indicating non-Curie behaviour. A fit using the Boltzmann function for a singlet-triplet (based on the notation $\boldsymbol{\widehat{H}_{HDvV} = -2J\widehat{S_1}\cdot\widehat{S_2}}$ obtained from the Heisenberg-Dirac-van Vleck Hamiltonian). Left: m-pyridine-*H*, right: benzylic C*H*$_2$.

IR Spectra

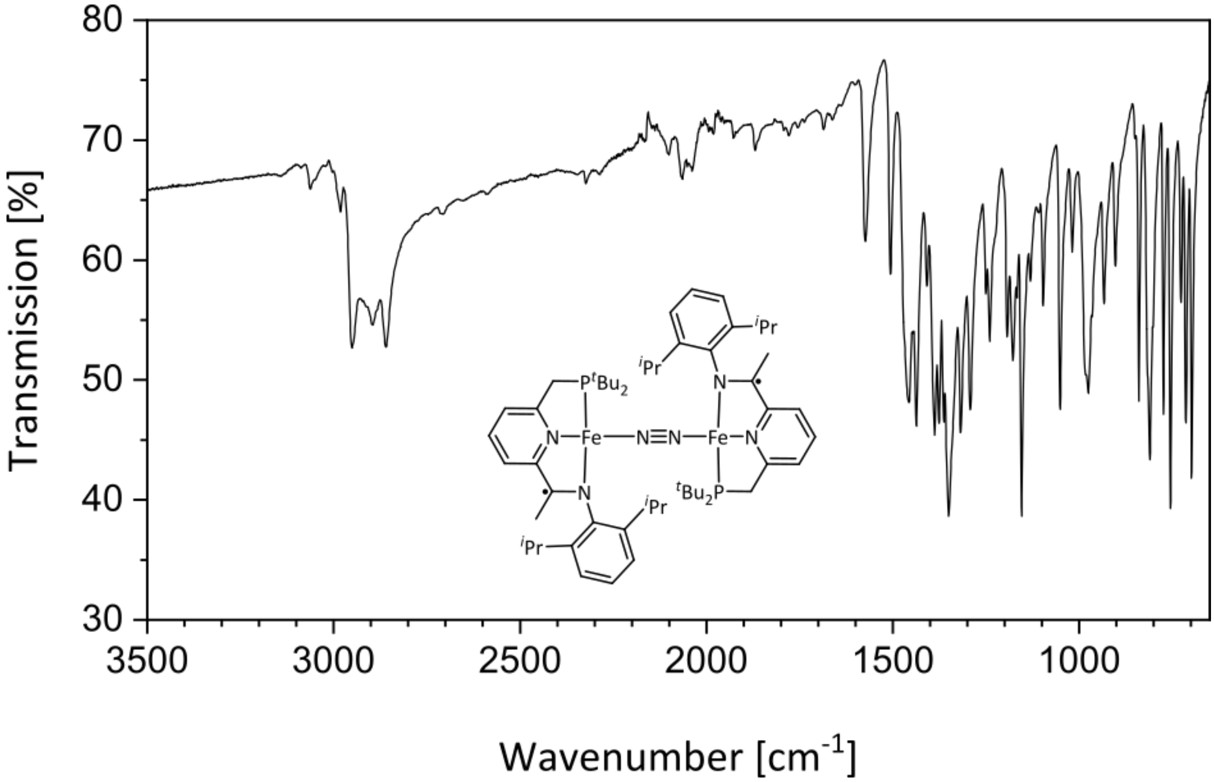

Figure S 20. ATR-IR spectrum of **3**.

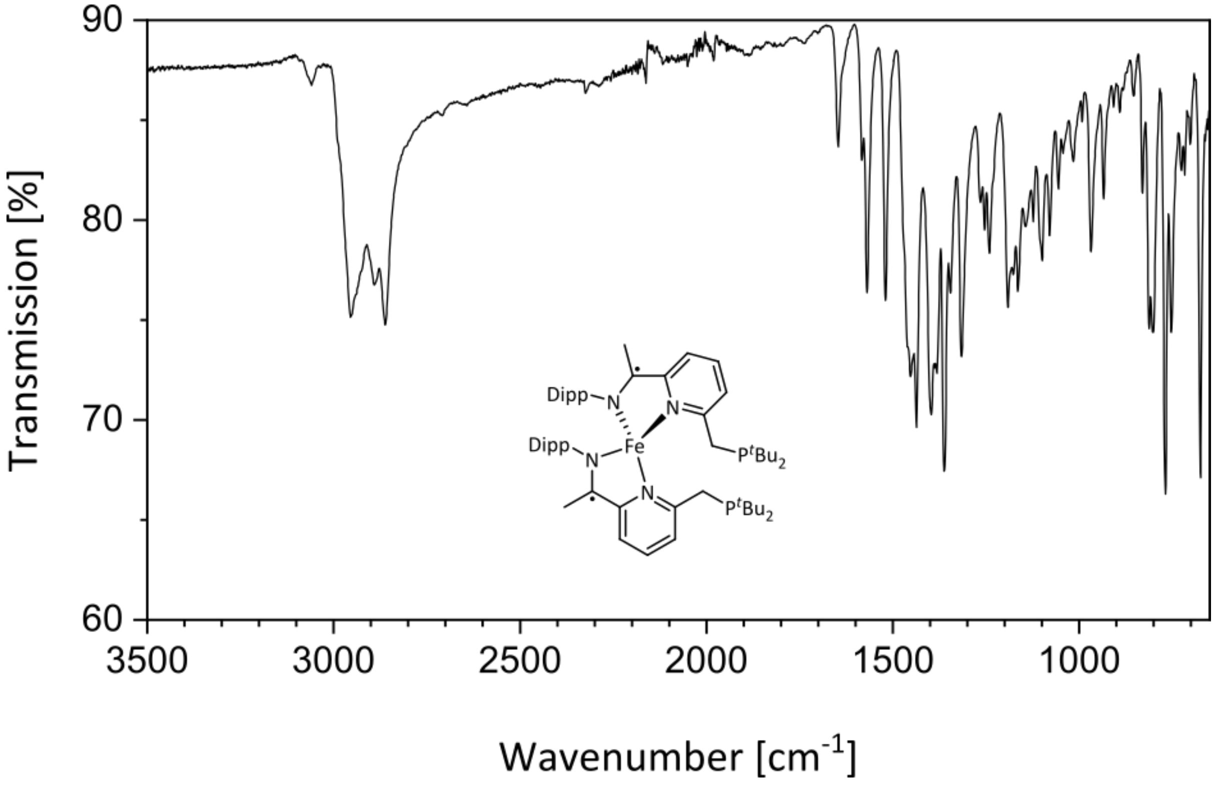

Figure S 21. ATR-IR spectrum of **4**.

Raman Spectra

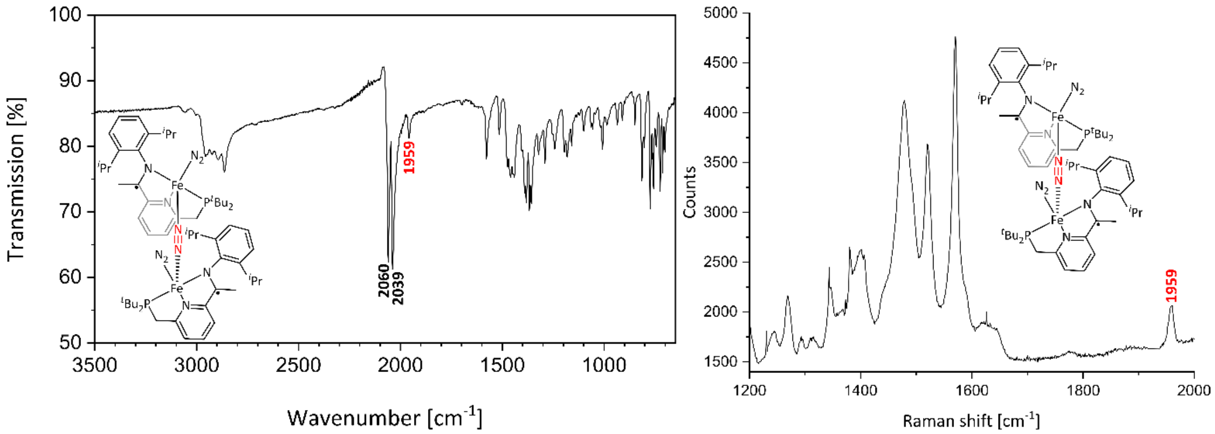

Figure S 22. ATR-IR spectrum (left) and Raman spectrum (right) of **2**, showing the IR and Raman active vibration of the bridging N_2 unit (highlighted in red).

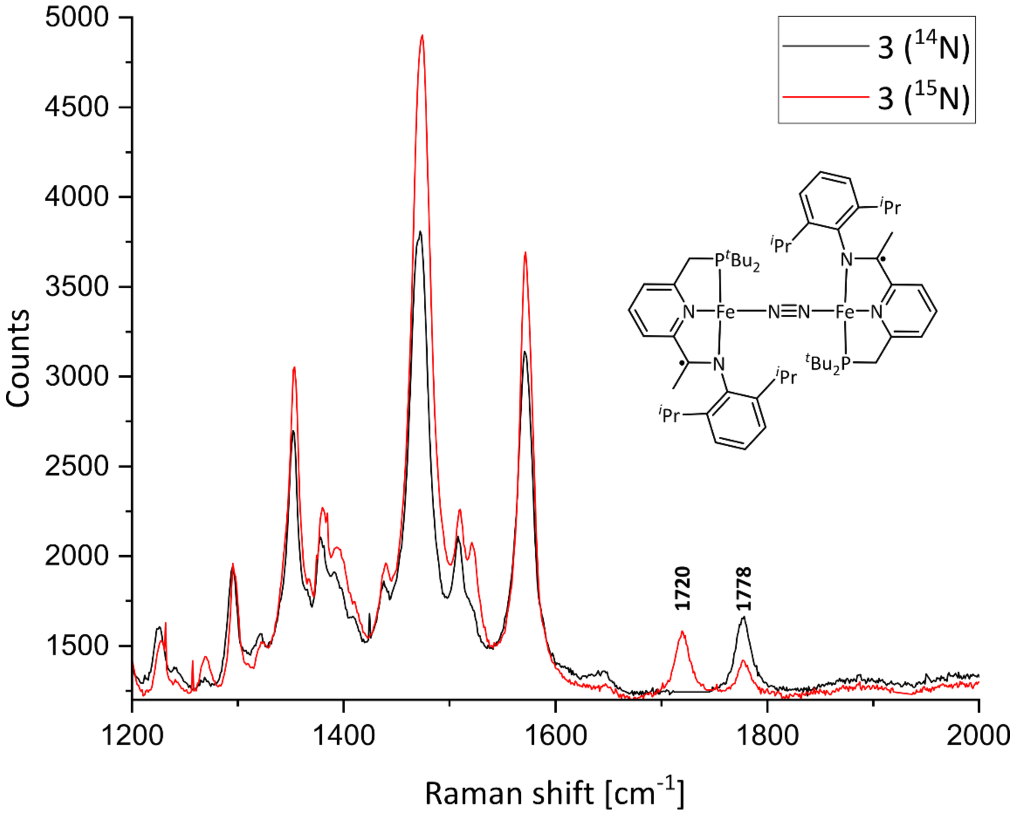

Figure S 23. Raman spectrum of $^{14}N_2$ (black) and $^{15}N_2$ enriched (red) samples of **3**.

<u>**Notes:**</u> Signals from cosmic radiation (**$^{14}N_2$-3**: 1886–1887 and 1951–1953 cm^{-1}, **$^{15}N_2$-3**: 1627 and 2095 cm^{-1}) as well as absorption dips resulting most likely from the materials used for preparation (**2**: 1920–1936 cm^{-1}, **$^{14}N_2$-3**: 1728–1742 cm^{-1}, **$^{15}N_2$-3**: 1920–1935 cm^{-1}) were removed to ensure clarity of the presented data.

Raw Data Spectra

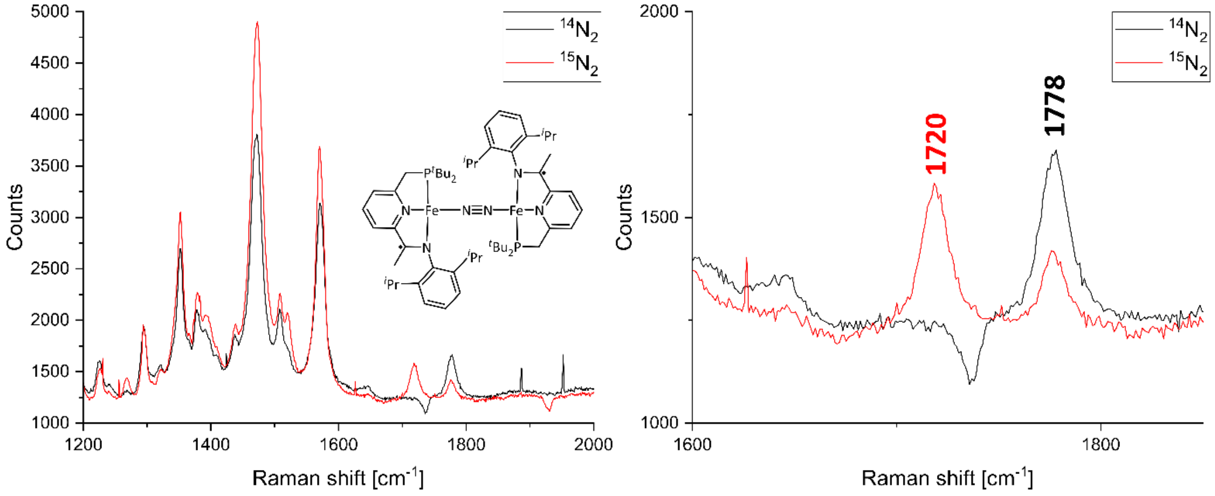

Figure S 24. Left: Overlayed Raman spectra of $^{14}N_2$ (black) and $^{15}N_2$ enriched (red) samples of **3** without corrections of cosmic radiation and absorption dips. Right: Section of the raman spectra focused on the N_2-vibration area.

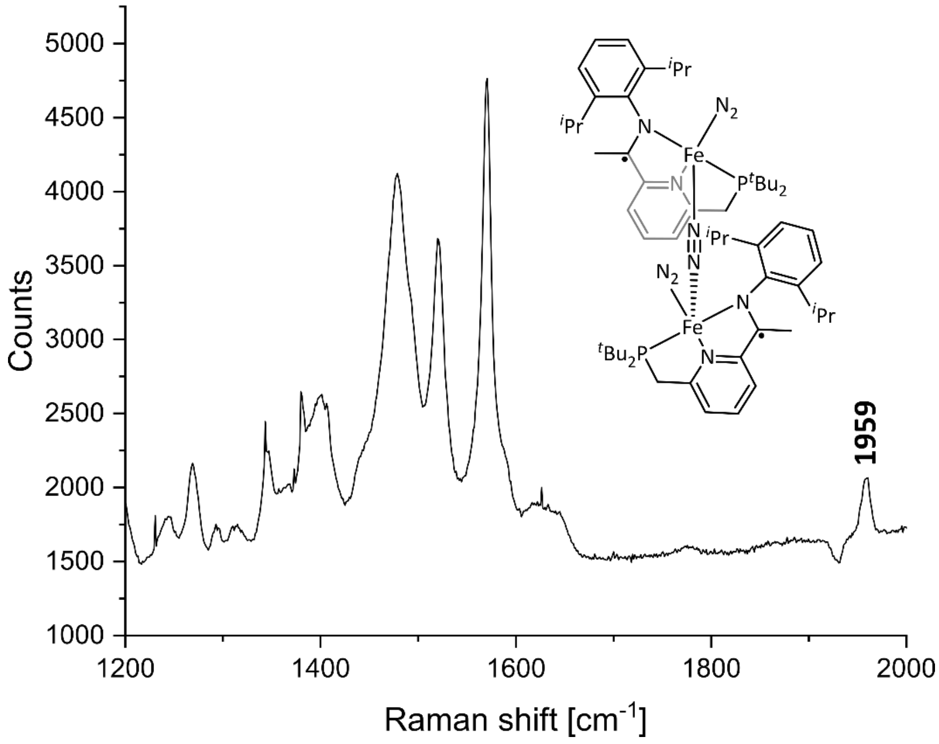

Figure S 25. Raman spectrum of **2** without corrections of cosmic radiation and absorption dips.

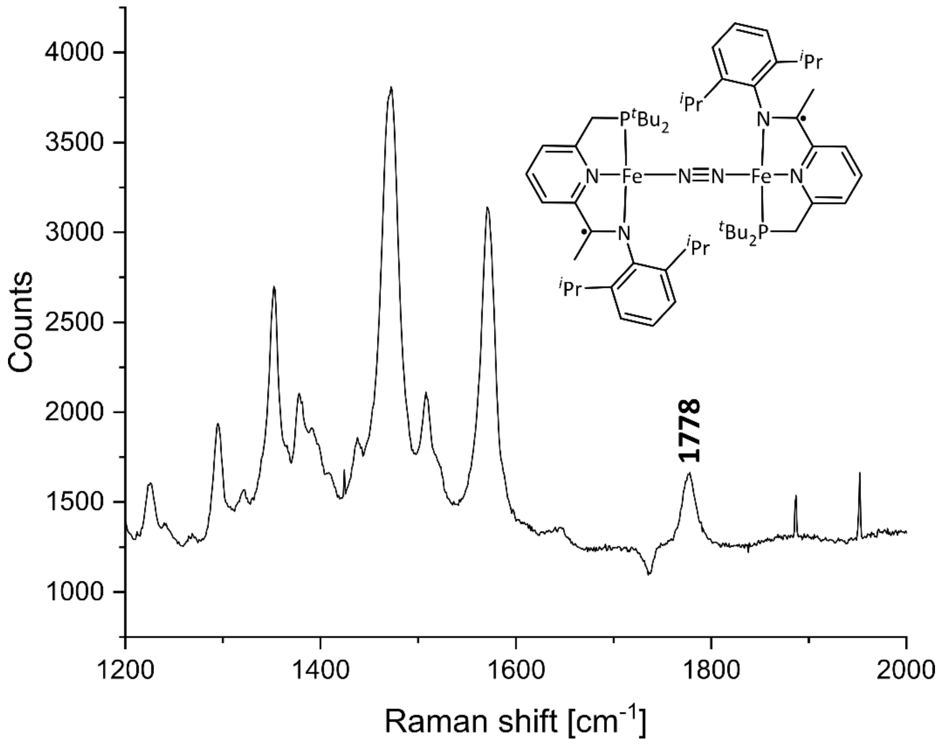

Figure S 26. Raman spectrum of $^{14}N_2$-**3** without corrections of cosmic radiation and absorption dips.

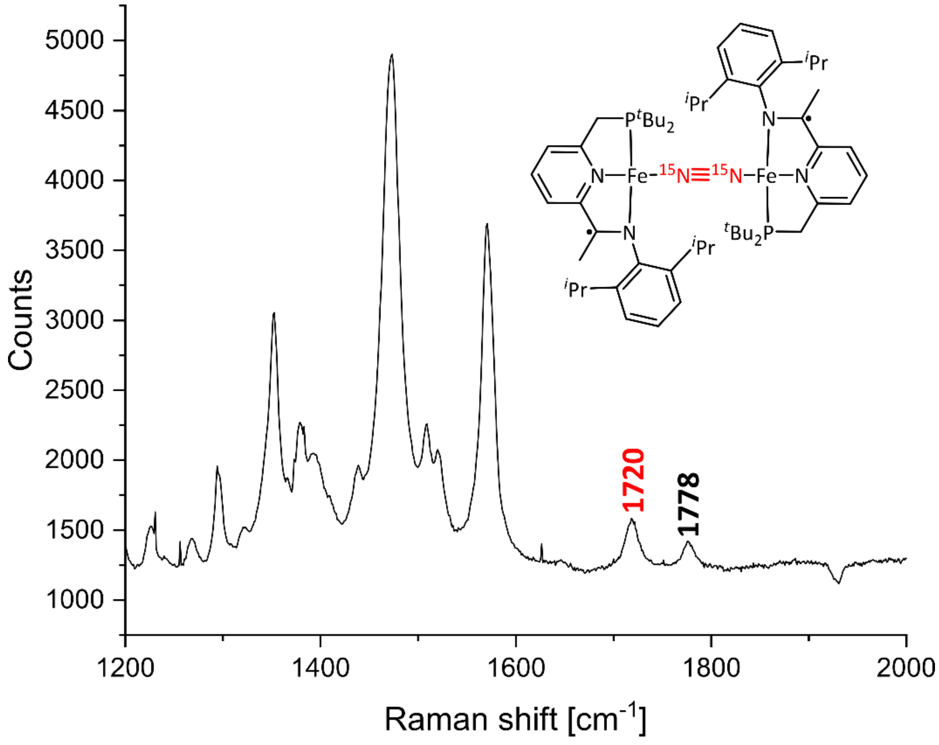

Figure S 27. Raman spectrum of a $^{15}N_2$ enriched sample of **3** ($^{15}N_2$-**3**) without corrections of cosmic radiation and absorption dips. Residual $^{14}N_2$-**3** labelled in **black**.

<u>Sample preparation:</u> In an argon filled glovebox a small piece of aluminium foil was placed on a microscope slide. The powdered sample was placed on top of the foil and sealed on top with a small rectangular piece cut from a microscope slide. To ensure exclusion of atmospheric oxygen or moisture the sides of the smaller glass were sealed using commercially available superglue (the capillary effect helps distributing the glue under the glass). The sample was left to sit for ca. 10 min and then brought out of the glovebox and left under ambient atmosphere over night to check for leaks (the compounds **2** and **3** are dark green or dark brown solids, upon exposure to oxygen or water they turn lightbrown). As an example, **Figure S24** shows one of the measured samples.

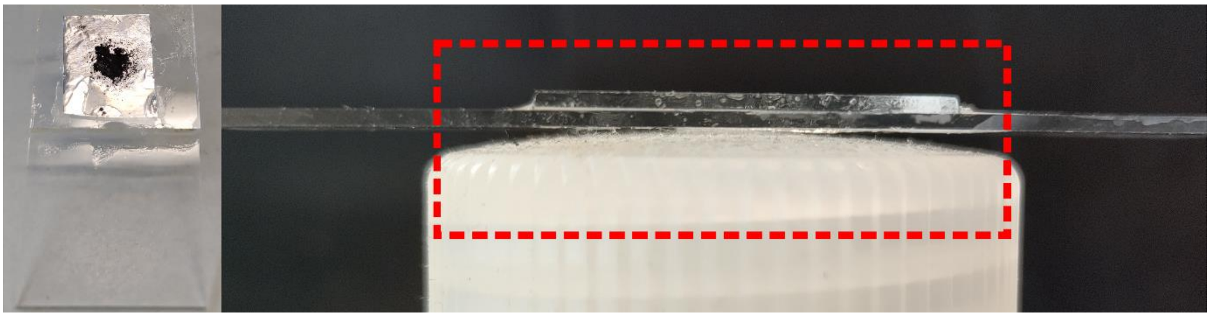

Figure S 28. Example of the sample preparation used to measure Raman spectra, highlighting the components (left) and the double-layered microscope slides (right).

UV-Vis Spectra

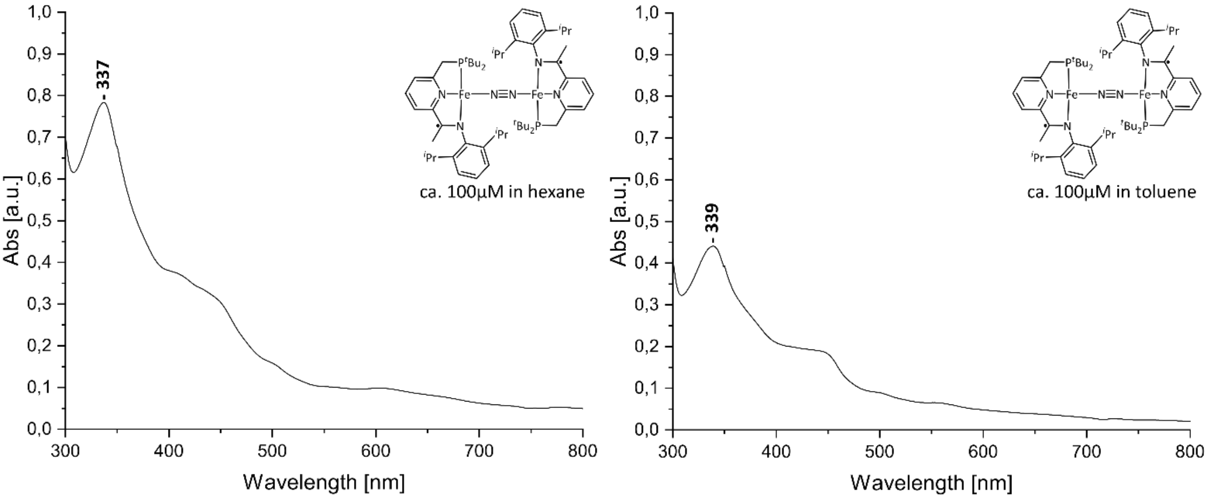

Figure S 29. Absorption spectra of **3** (ca. 100 µM) in hexane (left) and toluene (right).

Please note: because compound ***3*** *always contains ca. 17 % of the starting material (****2****), the concentration cannot be reliably determined.*

Computational Details

All density functional theory (DFT) calculations were performed using the ORCA quantum chemical program package (Version 4.2.1).[14] Geometry optimizations of the complexes **2–5** were performed using the corresponding crystal structures, without any truncation of their structures, as starting geometries. Geometry optimizations of all complexes were undertaken by employing the hybrid-GGA (GGA = generalized gradient approximation) density functional B3LYP[15,16] in conjunction with Ahlrichs triple-ζ def2-TZVP basis set[17] and the appropriate auxiliary basis set (def2/J)[18]. For **2–4** a basis set combination was used: def2-TZVP(-f) on Fe and the coordinating atoms (**2** and **3**: on N and P atoms; **4**: N atoms) and def2-SVP on all other atoms. To speed up the overall calculations, the RIJCOSX[19] approximation was applied for the expensive integral calculations. Noncovalent interactions were accounted for by using atom-pairwise dispersion corrections with Becke-Johnson damping (D3BJ).[20] Solvent effects were accounted for using the Conductor-like Polarizable Continuum Model (C-PCM)[21] with the dielectric constant of benzene. Subsequent numerical frequency calculations were undertaken for the optimized geometries to confirm they correspond to stationary points featuring no imaginary frequencies. To account for the basis set superposition error (BSSE) the geometrical Counterpoise correction (gCP)[22] as implemented in ORCA was used. To ensure the match of basis sets, single point calculations for **2**, **3** and N_2 employing the def2-TZVP basis set on all atoms as well as the gCP(DFT/TZ) keyword were used. Molecular orbitals were visualised with Avogadro (Version 1.2.0) and plotted with an isosurface value of 0.04.

Sample keyword line for geometry optimizations:

```
 ! UKS B3LYP D3BJ RIJCOSX def2-TZVP def2/J Pal16 TightSCF Grid4 FinalGrid5 GridX4 Opt
 xyzFile UCO Keepdens

%scf
 MaxIter 5000
end
%geom
 EnforceStrictConvergence True
end
%plots
 dim1 150 dim2 150 dim3 150
 Format cube
 SpinDens("filename.cube");
end

*xyz charge multiplicity
```

[14]a) Neese, F. *Wiley Interdiscip. Rev.: Comput. Mol. Sci.* The ORCA program system **2012**, *2*, 73-78; b) Neese, F. *Wiley Interdiscip. Rev.: Comput. Mol. Sci.* Software update: the ORCA program system, version 4.0 **2017**, *8*, e1327. Doi: 10.1002/wcms.1327

[15] Becke, A. D. *Phys. Rev. A.* **1988**, *38*, 3098-3100.

[16] Lee, C.; Yang, W.; Parr, R. G. *Phys. Rev. B.* **1988**, *37*, 785-789.

[17] Weigend, F.; Ahlrichs, R. *Phys. Chem. Chem. Phys.* **2005**, *7*, 3297-3305.

[18] Weigend, F. *Phys. Chem. Chem. Phys.* **2006**, *8*, 1057-1065.

[19] Neese, F.; Wennmohs, F.; Hansen, A.; Becker, U. *Chem. Phys.* **2009**, *356,* 98-109.

[20] a) Grimme, S.; Ehrlich, S.; Goerigk, L. *J. Comput. Chem.* **2011**, *32*, 1456-1465; b) Grimme, S.; Antony, J.; Ehrlich, S.; Krieg, H. *J.Chem.Phys.* **2010**, *132*, 154104.

[21] Barone, V.; Cossi, M. *J. Phys. Chem. A* **1998**, *102*, 1995-2001.

[22] Grimme, S.; Kruse, H. *J. Chem. Phys.* **2012**, *136*, 154101.

```
xyz coordinates from x-ray structure
*
```

Sample keyword line for frequency calculations:

```
 ! UKS B3LYP D3BJ RIJCOSX def2-TZVP def2/J Pal16 TightSCF Grid4 FinalGrid5 GridX4
 NumFreq MOread

% moinp "name_of_gbw_file.gbw"
%maxcore 2800
%scf
 MaxIter 5000
end
```

Broken-Symmetry Calculations

The broken symmetry (BS) formalism[23] was employed in unrestricted calculations to check for antiferromagnetic coupling of two spins. BS calculations were performed for all complexes using the B3LYP functional and the same basis set (def2-TZVP or def2-SVP//def2-TZVP) as mentioned earlier. In each case, multiple fragments were defined: PNN, Fe, N_2 and Br^-. Because several BS solutions of the spin-unrestricted Kohn – Sham equations may be obtained, the general notation BS(*m,n*) was used, where *m (n)* denotes the number of spin-up (spin-down) electrons at the iron centre (*m*) or the PNN ligand (*n*). For the dimeric complexes **2** and **3** the notation BS(n_1, m_1, m_2, n_2) was used, where the indices stand for the iron-PNN subunits, which are connected through a bridging N_2 ligand. The spin multiplicity for the broken symmetry calculations were chosen according to the high spin state – e.g. triplet for BS(1,1), quintet for BS(2,2), etc.

Input file example for the broken symmetry calculations, here: BS(1,1):

```
 ! UKS B3LYP D3BJ RIJCOSX def2-TZVP def2/J Pal16 TightSCF Grid4 FinalGrid5 GridX4 Opt
 xyzFile UCO Keepdens

%scf
 MaxIter 5000
 Brokensym 1,1
end
%geom
 ReducePrint false
 EnforceStrictConvergence True
end
%plots
 dim1 150 dim2 150 dim3 150
 Format cube
 SpinDens("filename.cube");
end
*xyz charge multiplicity
xyz coordinates from x-ray structure
*
```

[23] a) Ginsberg, A. P. *J. Am. Chem. Soc.* **1980**, *102*, 111-117; b) Noodleman, L.; Peng, C. Y.; Case, D. A.; Mouesca, J.-M. *Coord. Chem. Rev.* **1995**, *144*, 199-244.

^{31}P NMR Calculations

The NMR shifts were calculated from the optimized geometries using the averaged isotropic chemical shielding **σ** of the P atoms. As a reference the experimental ^{31}P NMR shift of PMe_3 [$\boldsymbol{\delta_{exp}(PMe_3)}$]was measured in benzene-d_6 and the isotropic chemical shielding calculated. The theoretical chemical shift of the molecule was determined as: $\delta_{calc} = \delta_{exp}(PMe_3) + (\sigma_{PMe3} - \sigma_{molecule})$.

Sample keyword line for NMR calculations:

```
 ! UKS TPSS0 RIJCOSX pcSseg-2 AutoAux CPCM(benzene) PAL16 TightSCF Grid6 NoFinalGrid
GridX8 NoFinalGridX NMR MOread

% moinp "name_of_gbw_file.gbw"
%maxcore 4800
%scf
  MaxIter 5000
end

*xyz charge multiplicity
xyz coordinates from x-ray structure
*
%eprnmr
  ori giao
  giao_2el = giao_2el_rijcosx
  nuclei = all P {shift}
end
```

Table S 2. Comparison of theoretical and experimental chemical shifts of **2** and **3**.

**Compound**	**Input**	**σ [ppm]**	**δ_{calc} ^{31}P [ppm]**	**δ_{exp} ^{31}P [ppm]**
**PMe_3**	RKS	397.192	–	-62.6
**2**	RKS	195.4845	139.1	113.7
	UKS	211.7935	122.8	113.7
	BS(1,1)	212.168	122.4	113.7
**3**	RKS	171.996	162.6	28.7
	UKS	207.878	126.7	28.7
	BS(1,1)	255.6155	79.0	28.7

Mößbauer Calculations

To compute Mößbauer parameters, single-point DFT calculations were performed for the geometry optimized structures using the B3LYP density functional in conjunction with the core properties basis set CP(PPP)[24] on Fe, def2-TZVP basis set,[25] for all other atoms. The RIJCOSX approximation was *not* applied.

[24] Neese, F. *Inorg. Chem. Acta* **2002**, *337*, 181.
[25] Schäfler, A.; Huber, C.; Ahlrichs, R. *J. Chem. Phys.* **1994**, *100*, 5829.

The isomer shifts (δ) were computed from the electron densities ρ_0 at the Fe nuclei using the linear equation:

$$\delta = \alpha \cdot (\rho_0 - C) + \beta \qquad (1)$$

where C is a constant, and α and β are the fitting parameters. Their values were obtained from previously reported DFT calibration work (B3LYP, α = −0.366, β = 2.852, C = 11810).[26] The quadrupole splitting parameter ΔE_Q was obtained from the electric field gradients V_{ij}. The asymmetry parameter was calculated as follows:

$$\eta = \frac{(V_{xx} - V_{yy})}{V_{zz}} \qquad (2)$$

Input file example for the Mößbauer calculations:

```
! UKS B3LYP D3BJ def2-TZVP CPCM(benzene) Pal16 TightSCF Grid4 FinalGrid5 MOread

%moinp "name_of_gbw_file.gbw"
%maxcore 2800
%scf
  MaxIter 5000
end
%basis
  newgto Fe "CP(PPP)" end
end
%method
  SpecialGridAtoms 26
  SpecialGridIntAcc 7
end
*xyz charge multiplicity
xyz coordinates from optimised structure
*
%eprnmr
  nuclei = all Fe {fgrad, rho}
end
```

Computational Summary

Table S 3. Optimization results of $[(PNN)Fe(N_2)]_2(\mu\text{-}N_2)$ (**2**) for various spin states. B3LYP, SVP//TZVP(-f). L = ligand.

Compound **2**	**Converged to**	**S_{ab} (UCO)**	**ΔG [kcal/mol]**
RKS	–	–	12.4
UKS (singlet)	BS(1,1,1,1) L^{up}-Fe^{down}-Fe^{down}-L^{up}	0.53/0.48	0.4
BS(1,1)	BS(1,1,1,1) L^{up}-Fe^{down}-Fe^{up}-L^{down}	0.52/0.49	0.0

[26] Römelt, M.; Ye, S.; Neese, F. *Inorg. Chem.* **2009**, *48*, 784.

BS(2,2)	BS(1,1,1,1) L^{down}-Fe^{up}-Fe^{up}-L^{down}	0.53/0.48	–
UKS (triplet)	L^{down}-Fe^{up}-Fe^{up}-L^{up}	0.51/0/0	3.7

Table S 4. Optimization results of [(PNN)Fe]$_2$(μ-N$_2$) (**3**) for various spin states. B3LYP, SVP//TZVP(-f). L = ligand.

Compound **3**	**Converged to**	**S_{ab} (UCO)**	**ΔG [kcal/mol]**
RKS	–	–	35.1
UKS (singlet)	BS(1,1,1,1) L^{up}-Fe^{down}-Fe^{down}-L^{up}	0.05/0.04	5.5
BS(1,1)	BS(1,3,3,1) L^{down}-Fe^{up}-Fe^{down}-L^{up}	0.48/0.45/0.06/0.03	0.0
BS(2,2)	BS(1,3,3,1) L^{down}-Fe^{up}-Fe^{down}-L^{up}	0.48/0.45/0.06/0.03	–
UKS (triplet)	BS(1,3,1,1) L^{down}-Fe^{up}-Fe^{up}-L^{down}	0.46/0.08/0/0	-1.1

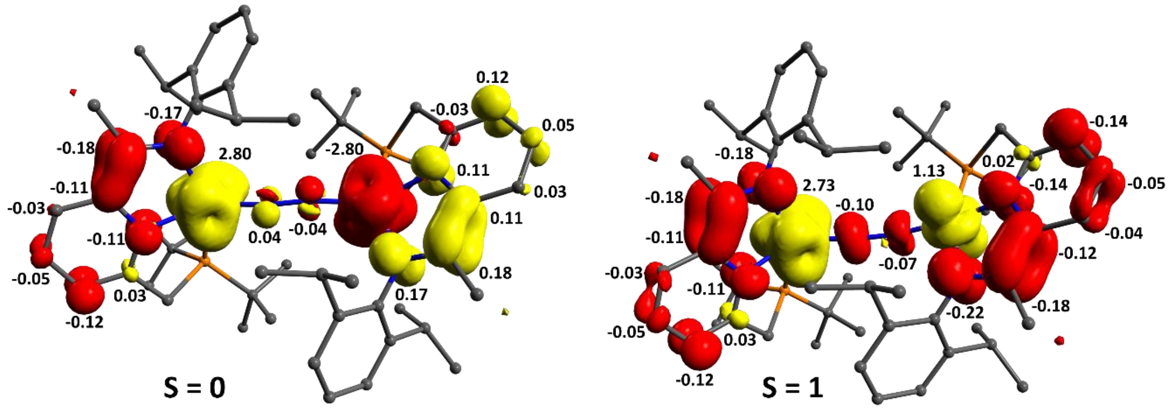

Figure S 30. Spin density plots of different electronic states of complex **3**. Left: BS(1,1) solution [conv. to BS(1,3,3,1)], right: UKS(triplet) solution [conv. to BS(1,3,1,1)]. Löwdin population analysis (LPA) shown, isovalue 0.005.

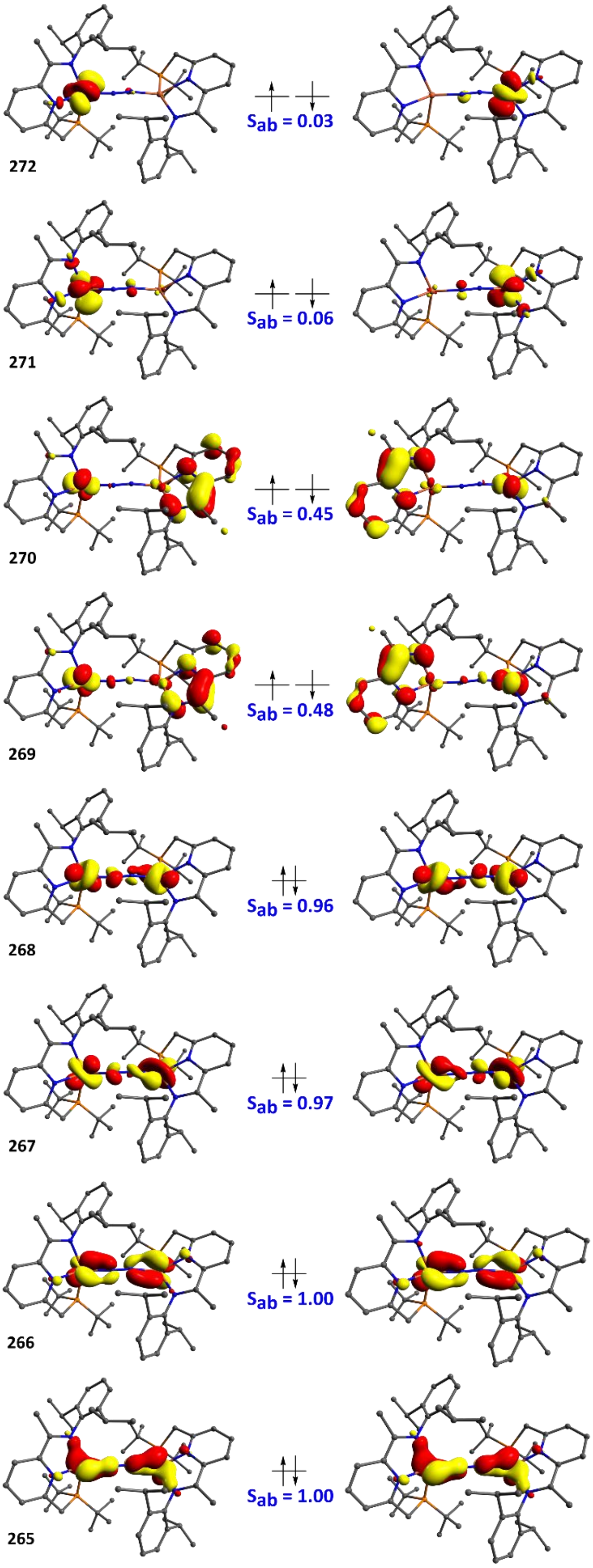

Figure S 31. Qualitative molecular orbital diagram of the magnetic orbitals (UCOs) derived from a BS(1,3,3,1) solution of **3**, plotted with an isovalue of 0.04.

Table S 5. Optimization results of $(PNN)_2Fe$ (**4**) for various spin states. B3LYP, SVP//TZVP(-f).

Compound **4**	**Converged to**	**S_{ab} (UCO)**	**ΔG [kcal/mol]**
UKS (triplet)	–	0.90/0.68/0/0	23.0
BS(4,2)	BS(4,2)	0.46/0.37/0/0	0.0

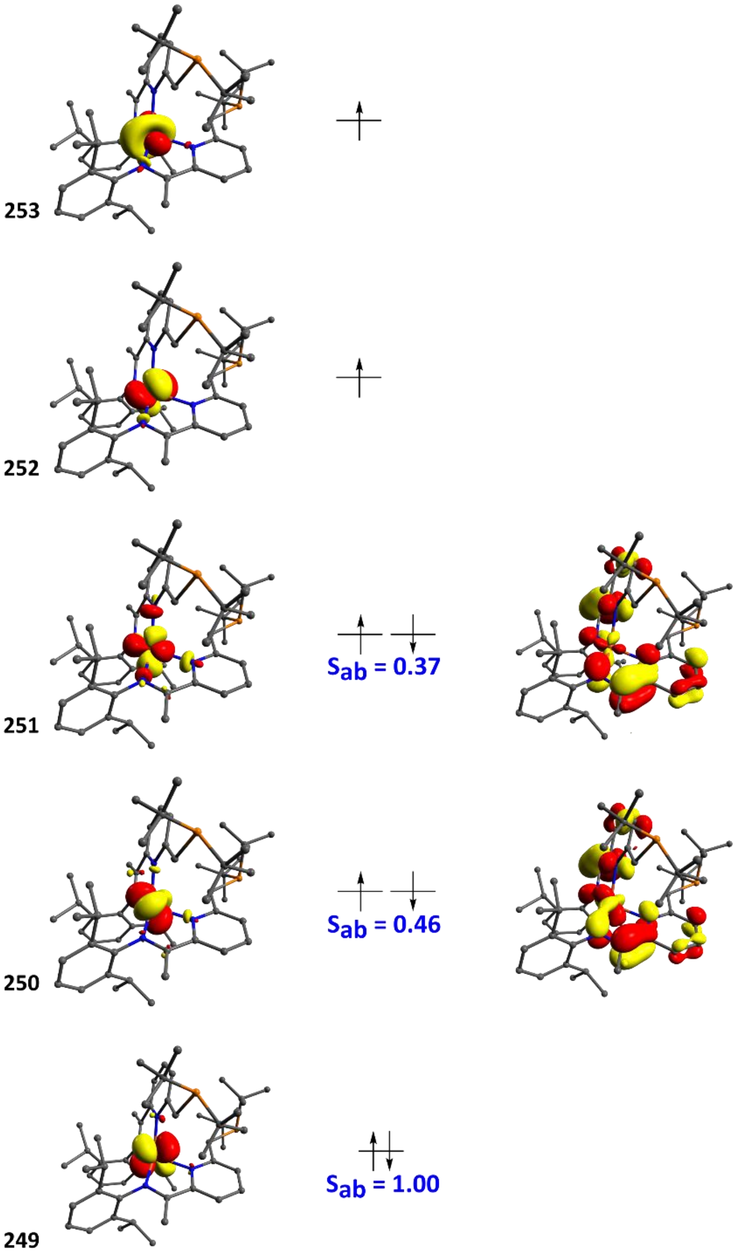

Figure S 32. Qualitative molecular orbital diagram of the magnetic orbitals (UCOs) derived from a BS(4,2) solution of **4**, plotted with an isovalue of 0.04.

Table S 6. Optimization results of (PNN)FeBr (**5**) for various spin states. B3LYP, TZVP.

Compound **5**	**Converged to**	**Geometry**	**S_{ab} (UCO)**	**ΔG [kcal/mol]**
UKS (doublet)	BS(2,1)	square planar	0.42/0	1.7
UKS (quartet)	BS(4,1)	dist. tetrahedral	0.57/0/0/0	0.0

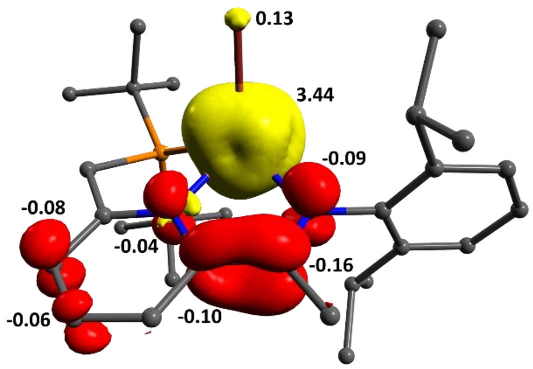

Figure S 33. Spin density plot for **5** from a BS(4,1) solution. Löwdin population analysis (LPA) shown, isovalue 0.005.

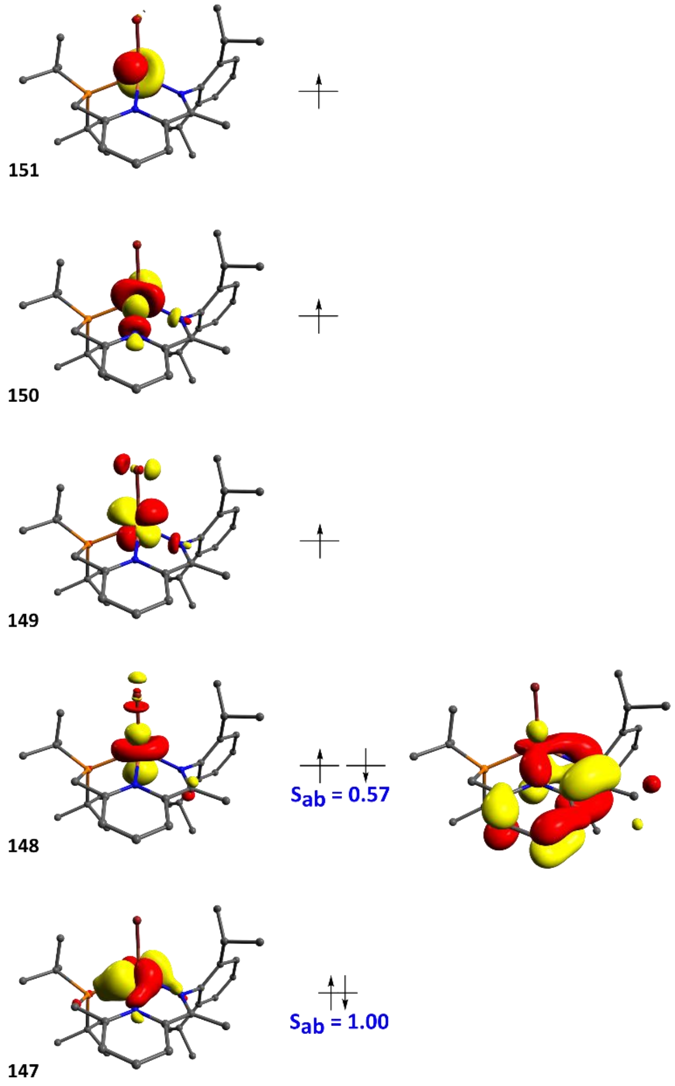

Figure S 34. Qualitative molecular orbital diagram of the magnetic orbitals (UCOs) derived from a BS(4,1) solution of **5**, plotted with an isovalue of 0.04.

Calculation of the N_2 dissociation free energy

Table S 7. Calculated Gibbs free energies and free energies of the dissociation of N_2 for the reaction: [(PNN)Fe(N_2)]$_2$(μ-N_2) (**2**) –> [(PNN)Fe]$_2$(μ-N_2) (**3**) + 2 N_2.

**Compound [input]**	**Corrections**	**G [Eh]**	**ΔG [kcal/mol]**
**2 [BS(1,1)]**	none	-5939.49700	**+7.7**
**N_2**	none	-109.5323008	
**3 [BS(1,1)]**	none	-5720.420159	
**2 [BS(1,1)]**	solvent(benzene)	-5939.50691	**+7.1**
**N_2**	solvent (benzene)	-109.5326528	
**3 [BS(1,1)]**	solvent (benzene)	-5720.430252	
**2 [BS(1,1)]**	solvent, BSSE (gCP)	-5941.745900	**+0.7**
**N_2**	solvent, BSSE (gCP)	-109.534787	
**3 [BS(1,1)]**	solvent, BSSE (gCP)	-5722.675136	

XYZ Coordinates of Optimized Structures

Optimized coordinates for **2** (UKS BS(1,1) CPCM(benzene)). Final Gibbs free energy: -5939.50690534 Eh.

Fe	-5.70780828348034	-9.35743317579150	-17.81642021070024
Fe	-7.03716133580551	-14.05943671946882	-18.35922769008852
N	-5.56940984667293	-9.00830239338233	-15.88379405684399
N	-7.60925493646703	-9.16393228859426	-17.38420586362381
P	-6.40724840461127	-8.60946348216528	-19.83479597059573
C	-7.88792087392802	-9.07372098940422	-16.02697662916743
C	-9.24048150125595	-9.04844246928848	-15.59332483791603
H	-9.46206080248269	-9.03255954692295	-14.52548903463297
C	-10.25781874308258	-9.03246081494392	-16.52527587195235
H	-11.30101185688443	-9.00393705001573	-16.20178900401218
C	-9.94218241247235	-9.04016206875791	-17.90878583804809
H	-10.72295248347474	-8.99941437436272	-18.66894157214149
C	-8.61025684954411	-9.11982519960560	-18.29151046421566
C	-8.16991191242476	-9.15588400412276	-19.73029297190199
H	-8.86159942761404	-8.59624078671969	-20.37615771257512
H	-8.18357479219796	-10.19786494058129	-20.08380595836319
C	-6.48251704792368	-6.71912158741265	-19.93632289568037
C	-7.31566361409314	-6.23119180645584	-18.73366524168362
H	-7.28505056180309	-5.12965295309780	-18.70551846805546
H	-6.91952519875471	-6.60784652461539	-17.78106973049780
H	-8.36977270138205	-6.53502717400390	-18.80364108233590
C	-7.12009859888731	-6.17652212839983	-21.22289117763777
H	-8.13086526354614	-6.58106027332047	-21.38564344655948
H	-6.51276328361594	-6.39233369777418	-22.11225074334461
H	-7.21264761390255	-5.07982274643316	-21.14744820831919
C	-5.05619898872451	-6.15988864042442	-19.77893069623136
H	-5.10574156057326	-5.06617282951056	-19.64823953409027
H	-4.42321724229533	-6.36126200210893	-20.65123293576763
H	-4.55886432059882	-6.58011677175514	-18.89501697445808
C	-5.83280594078964	-9.33097899872995	-21.48288159968163
C	-6.90232902767802	-9.28493540854205	-22.58825075838526
H	-7.23724151397162	-8.26625382069146	-22.81885887819412
H	-7.78215743097233	-9.89013484559681	-22.32759208357444
H	-6.47917468329071	-9.71253953822242	-23.51348223558604

C	-4.54228992857212	-8.64510165559388	-21.96057065747411
H	-4.11989886730144	-9.22212882166590	-22.80012175845517
H	-3.78084006556575	-8.60951340075122	-21.16651086557219
H	-4.71653897491747	-7.62199226604652	-22.32052725486501
C	-5.49983910508971	-10.80107767925503	-21.18346704297170
H	-6.35200148763800	-11.34990152695335	-20.76488548683076
H	-4.66688674873421	-10.88246401418467	-20.47534977134237
H	-5.20806387633056	-11.30656890918155	-22.11784522095272
C	-6.73298982720218	-8.97947816429884	-15.21077795409400
C	-6.82093303259974	-8.87455878617172	-13.71506518786255
H	-6.98820548842575	-9.86486244071222	-13.25461090222762
H	-7.65290506458181	-8.22579706145313	-13.40193678526353
H	-5.89279953663082	-8.47075214954277	-13.29037057425252
C	-4.33355031648799	-8.81837973388183	-15.20676215663383
C	-3.72888485600956	-7.53673654609771	-15.23848328837911
C	-4.41247116314418	-6.38210040856433	-15.95706206785869
H	-4.98215907367280	-6.83544293966972	-16.78190648490417
C	-3.43227447830286	-5.37620894435698	-16.56936032155013
H	-2.89269845644262	-4.80258675200356	-15.79816154343688
H	-3.97744205833475	-4.64972252075477	-17.19216422884103
H	-2.68493714538206	-5.87649816788479	-17.20499672525028
C	-5.43114337926365	-5.66694757134422	-15.05550846755020
H	-6.19224047391100	-6.36352572871540	-14.67803483005523
H	-5.95082773070023	-4.87077579442421	-15.61419316074289
H	-4.93087308118534	-5.20461716313208	-14.18801051438546
C	-2.49503138770067	-7.36442822118418	-14.59819823390235
H	-2.01725780250870	-6.38307564991753	-14.60553632917024
C	-1.85820539612194	-8.42856800801552	-13.95677498161187
H	-0.89429295328550	-8.27491569283839	-13.46486418214692
C	-2.45053642188390	-9.69028795550940	-13.95997736030514
H	-1.94113769022790	-10.52660440112879	-13.47465689801988
C	-3.68888142341574	-9.90680544158444	-14.58034823069803
C	-4.29219770267112	-11.30011543789607	-14.62287308042341
H	-5.30587501401031	-11.19756861115746	-15.02457163849545
C	-3.51255242975947	-12.19425325172788	-15.59749164096521
H	-3.45492151777861	-11.72822800426932	-16.59006379287938
H	-4.00251950158515	-13.17494793217612	-15.71197298569491
H	-2.48296639337813	-12.36790679170369	-15.24362333457863

C	-4.40093968450496	-11.94254071734709	-13.23423095757090
H	-3.40921243339537	-12.11475295354506	-12.78558496976050
H	-4.90546432631208	-12.91968131929273	-13.30078806582399
H	-4.97711062464919	-11.30624575620935	-12.54372287450022
N	-3.92334968726248	-9.21884352481949	-18.13476279307510
N	-2.83350370925967	-9.11369136172282	-18.32550976133904
N	-5.89025367883933	-11.27353745184548	-18.02423029139027
N	-6.22456252442279	-12.33888021924246	-17.99168113423356
N	-6.29835986225700	-14.69660951116714	-20.07330567399832
N	-8.31433371405020	-13.23060455426466	-19.59103149939165
P	-8.82461660810461	-13.99687123479468	-16.96643177202850
C	-7.99544262756711	-13.36296347623219	-20.93495854420916
C	-8.79660521517373	-12.71036564908385	-21.90950942122039
H	-8.52018060263461	-12.77462871858926	-22.96265550870240
C	-9.91925882981373	-12.01114664904932	-21.51592899287151
H	-10.54572544514784	-11.51198464873078	-22.25882256101794
C	-10.26319921163143	-11.95286595453118	-20.14005662756778
H	-11.15695206513191	-11.42994584090276	-19.79908091835451
C	-9.42849169943251	-12.56443599192808	-19.21487632859521
C	-9.69591550959243	-12.55735590814642	-17.73362866218585
H	-10.77281161080107	-12.50336699250369	-17.52050499617423
H	-9.24090504837944	-11.66035628320862	-17.28740409844359
C	-10.01083355891174	-15.46007129656000	-17.18660993696738
C	-9.28260960916806	-16.75529062632456	-16.78272134357888
H	-9.88966344216639	-17.62377669817462	-17.08818338420037
H	-9.11751245675481	-16.83095248228702	-15.70164849008986
H	-8.30841596599592	-16.83562590100646	-17.28265096423819
C	-10.34133803389992	-15.55973848346194	-18.68997131272537
H	-9.43432561406415	-15.64121322118578	-19.30427409844630
H	-10.91670738953435	-14.69603966964750	-19.05106008102063
H	-10.95286679088722	-16.46163543468458	-18.85701005863645
C	-11.32647364348359	-15.32940907366474	-16.40719086689558
H	-11.99735795897185	-16.16031856353113	-16.68410393049360
H	-11.85272968174943	-14.39131974274306	-16.64172332595623
H	-11.17540550347261	-15.38040793566974	-15.32038335755873
C	-8.71233485436662	-13.51458583503713	-15.14417900934828
C	-9.95821338616936	-12.78276272863271	-14.61596898376594
H	-10.86949437022948	-13.38958782969437	-14.68290224561030

H	-10.13136254575621	-11.83548431920331	-15.14657292336459
H	-9.80143665984828	-12.53467867196719	-13.55211571619975
C	-8.41316707410051	-14.74518917300806	-14.27273808225071
H	-8.14044138710150	-14.40835271640151	-13.25870480617621
H	-7.56797322747119	-15.33096316801753	-14.66525459800264
H	-9.28188574193045	-15.41040976284964	-14.17460336153646
C	-7.50843114548083	-12.56431623740494	-15.04722756968489
H	-7.62167633411797	-11.67403554018879	-15.67788353205261
H	-6.57878769165145	-13.06806989338534	-15.33688402690552
H	-7.40129548060353	-12.21990476462652	-14.00619486078201
C	-6.87228729537761	-14.19154522707417	-21.17944376985891
C	-6.37661480923047	-14.45693232476052	-22.57280285097015
H	-7.20606642547128	-14.68757892508059	-23.25963817102030
H	-5.67191592678846	-15.29802162629931	-22.59107141348092
H	-5.85201301132178	-13.57613786830403	-22.98451271333102
C	-5.20429167887667	-15.59963578505396	-20.16523810613868
C	-5.44822319755904	-16.98513393874231	-19.99685417257099
C	-6.86484561024047	-17.49527245097845	-19.77802051883750
H	-7.40759735681811	-16.68315055714826	-19.27087500931866
C	-6.93435026595627	-18.73305902408367	-18.87787527120808
H	-6.49946388263533	-19.62221590752452	-19.36270961470709
H	-7.98328450674604	-18.97198007419045	-18.64241200449159
H	-6.39981944063466	-18.56861598668787	-17.92923866316854
C	-7.58477050045528	-17.74544465090912	-21.11282088599048
H	-7.61624498083111	-16.83402159457518	-21.72662932235727
H	-8.62343059056810	-18.07328103106900	-20.93944573707275
H	-7.07061526969234	-18.52988989995212	-21.69327583714126
C	-4.36133569247667	-17.86729396680563	-20.04798409832526
H	-4.53149331668781	-18.93884492567009	-19.92650485365592
C	-3.06042394023626	-17.40143935900593	-20.24720133944342
H	-2.22485862592939	-18.10554606493895	-20.28346042158544
C	-2.83204355848383	-16.03349757070581	-20.38700080699250
H	-1.81099473331280	-15.66861804783619	-20.52527671613841
C	-3.88994641793076	-15.11465798907499	-20.34716290166292
C	-3.61332326760833	-13.62443985337891	-20.44487160364153
H	-4.58383456389731	-13.12405786234376	-20.52426478408360
C	-2.94923616652085	-13.11207386471004	-19.15874764975532
H	-3.55831137623133	-13.36462564079664	-18.28056411378385

H	-2.82960012024695	-12.01654522390215	-19.18553169127635
H	-1.95075275889042	-13.55782310204953	-19.01926169228574
C	-2.79222962740464	-13.25100888263470	-21.68574655964623
H	-1.77987428071944	-13.68500990288196	-21.65009046643944
H	-2.67871627290229	-12.15718408350937	-21.75656227761628
H	-3.27757214656602	-13.60565289702206	-22.60884359788188
N	-5.96813532807075	-15.09927844913739	-17.31597778859007
N	-5.32407488447268	-15.74820735767148	-16.68415693907809

Optimized coordinates for **3** (RKS). Final Gibbs free energy: -5720.36421987 Eh.

P	11.31618575906428	11.10414329288798	4.76320047263284
N	11.41542872094566	13.73384645841541	5.51308853233996
N	9.67882669059287	13.88189409873938	7.26008871163590
C	12.03919373143401	12.37498623855805	3.62366663158395
H	12.93349753163474	12.06547658256821	3.06498976984157
H	11.24292433113059	12.59472025047254	2.89502866412958
C	8.59783618382614	13.70615532231987	8.15864214233310
C	11.50109000159893	14.88517473768048	6.29512779147957
C	13.24658150187137	14.54696646409070	4.17484371973357
H	13.91940553896164	14.38766428825987	3.32944104794028
C	12.27611255906222	13.60891107140564	4.44754029262693
C	12.47852128477936	15.87127888516606	6.03330912845846
H	12.51526146371149	16.76138518005039	6.66442129326526
C	10.59109294202459	9.76092746481232	3.65202368518821
C	8.60020697650572	12.53246379444070	8.95571138168528
C	13.35977474127484	15.71126050388502	4.98442639730774
H	14.12113110151535	16.46318233394992	4.76861885532445
C	7.42198527221379	15.82977399719611	7.29414100208515
H	8.38847869977412	15.94417670963754	6.79029602768445
C	9.73507723954712	10.48291977476723	2.59779537426395
H	9.04150719306353	11.21137698942561	3.03618383457347
H	9.13766178499565	9.73646635236633	2.05081389259988
H	10.34545961605161	11.01118283292764	1.85427671992049
C	12.82171731688084	10.42353035683341	5.70971820279812
C	12.41758762537552	9.14200108189003	6.45776983978863
H	11.50274663430664	9.29150233715249	7.05123640673308
H	13.22262105763300	8.85459271868510	7.15449662060534

H	12.25251016044666	8.29336117017353	5.78001602219529
C	13.23059441920715	11.47695081606448	6.75897475632308
H	13.60883012295480	12.39725035140553	6.29626754632209
H	14.03493084273813	11.05870840788704	7.38751055315628
H	12.39267176479274	11.75730510640097	7.40665170330892
C	7.50285830463180	14.60140148684505	8.19142762636256
C	6.44228958819337	13.17921222508944	9.86771607802503
H	5.60247024143444	12.97737282800648	10.53782890050280
C	7.52460717094507	12.29658669880563	9.81457521960638
H	7.51910163123392	11.40449957035490	10.44297763727586
C	14.04660857011116	10.15275602554645	4.82139793983831
H	13.85842629390022	9.41164587782302	4.03614776417705
H	14.86874414493878	9.76835378261992	5.44931208677896
H	14.41032736021906	11.07544437971123	4.34631363601979
C	9.78723516893425	11.58453694735238	8.87357673594917
H	10.10899158106350	11.56735757105979	7.80231584047654
C	10.52032068018841	14.91684714817170	7.31734591284703
C	6.37393976301703	15.63671827069484	6.19235854474894
H	6.57775667924556	14.73663583188726	5.59807279963566
H	6.36011318547970	16.50228518911641	5.51087981516058
H	5.36134527988809	15.53137858029840	6.61208068777574
C	11.59774156552142	8.86350455171391	2.91782097128300
H	12.30225287225442	9.44929486210739	2.30756507439317
H	11.05095300536697	8.19174869507924	2.23381508562571
H	12.17705472539381	8.22725512847007	3.60063565541560
C	6.43299127876194	14.30812005344959	9.05218605385642
H	5.57361680714094	14.98291000562413	9.07811830544782
C	10.50328614157840	15.97083914985020	8.38687222964627
H	9.82834889456383	15.69106066758592	9.20569211005203
H	10.17305487913127	16.95029448269677	8.00217749463836
H	11.51204469864459	16.11218521958879	8.80648305569038
C	9.65534754258192	8.90363321773221	4.52656180579486
H	10.20380311699643	8.27018736580448	5.23565150404925
H	9.05164467349740	8.23920694151983	3.88564181865224
H	8.96617791284175	9.53639904789759	5.10209692118888
C	7.14651674215604	17.11664659622351	8.08633244399075
H	6.13670011031697	17.10856019253124	8.52796630392611
H	7.20266670303996	17.99360307290854	7.42045003701429

H	7.86680951246056	17.26047468009617	8.90584652371150
C	9.47658912543582	10.13832939451526	9.26108525636056
H	9.23456858990721	10.04070638861422	10.33215609422293
H	10.35563965984646	9.50226786403057	9.07183516517556
H	8.63415398635914	9.73532929319543	8.68086328101880
C	10.97287560585348	12.12126185942767	9.68976951085333
H	11.28779389908149	13.11342412820687	9.33647606105106
H	11.83869673858623	11.44354879490128	9.62106174795809
H	10.69317086895374	12.20829115558467	10.75255398760173
Fe	10.04002368868541	12.52516497038443	5.92587516699380
N	8.37512369313168	12.21584466671097	5.30091657827381
N	7.34922138680369	12.21566140713170	4.81292619866912
Fe	5.68414767213651	12.52442204585930	4.18836485734986
P	4.40930199320162	11.10296975051516	5.35107582784227
N	4.30836749863132	13.73248045913431	4.60132979480010
N	6.04526872306478	13.88184625556057	2.85462398804703
C	3.68530504526051	12.37319702540627	6.49066592966469
H	2.79114713206336	12.06303545211758	7.04920864136164
H	4.48133730565715	12.59339573509721	7.21942277070981
C	7.12652376441020	13.70669376146482	1.95633190931581
C	4.22238592666111	14.88400921941554	3.81960370762721
C	2.47687738678499	14.54466885443007	5.93969020473861
H	1.80413479530160	14.38501148901634	6.78509264157159
C	3.44771589522554	13.60704571548022	5.66687443860157
C	3.24456711911484	15.86968226447673	4.08157577651327
H	3.20753737317022	16.75991797980809	3.45066363574811
C	5.13536525491972	9.76025709545565	6.46223978992904
C	7.12489247836353	12.53293548193704	1.15934603982450
C	2.36328560751809	15.70909436108347	5.13034650023393
H	1.60168503227253	16.46071918873722	5.34632231467753
C	8.30128451990008	15.83083945858741	2.82107521800907
H	7.33472846728375	15.94471412998785	3.32492246490936
C	5.99106638343446	10.48281594943864	7.51633939242224
H	6.68429515083433	11.21153357921766	7.07785444451400
H	6.58886136199652	9.73673134529870	8.06340989571578
H	5.38047595974436	11.01088656859784	8.25982369328965
C	2.90414132202879	10.42134305532759	4.40460787385812
C	3.30899201089477	9.14003435543006	3.65657759897469

H	4.22376200542026	9.29003552653963	3.06313257401831
H	2.50413338043807	8.85216951789458	2.95983757185976
H	3.47452240619228	8.29149459237293	4.33434275561916
C	2.49457161255051	11.47448440261233	3.35533711272487
H	2.11600588624406	12.39465720359451	3.81803385441003
H	1.69029373922466	11.05581749304294	2.72700546310808
H	3.33221252862563	11.75517475851710	2.70743249661332
C	8.22112203524781	14.60240993718740	1.92379462650110
C	9.28278067532838	13.18055344516689	0.24790834851014
H	10.12288963314788	12.97901553890428	-0.42193214749933
C	8.20085312906117	12.29743146867987	0.30084063877090
H	8.20692809192934	11.40531247857143	-0.32750719896826
C	1.67940578570420	10.14987435351880	5.29293471188535
H	1.86805477055000	9.40903619254834	6.07832887807518
H	0.85756232517673	9.76479039244147	4.66505351257735
H	1.31501874614478	11.07239694336863	5.76783180635223
C	5.93824842112755	11.58450952533391	1.24125279616683
H	5.61614979123952	11.56722660252010	2.31244773975781
C	5.20331923010867	14.91640053856532	2.79754607303014
C	9.34944193473797	15.63836605484631	3.92285739050139
H	9.14608063172376	14.73819835958718	4.51717671692024
H	9.36284077583407	16.50397832032670	4.60428797128260
H	10.36208089092785	15.53350474798387	3.50312561667931
C	4.12933610004814	8.86228736201957	7.19663086103573
H	3.42456440701328	9.44770859080285	7.80694133680333
H	4.67661652068637	8.19090985064300	7.88061512909265
H	3.55031286913141	8.22564339915245	6.51394138311764
C	9.29133550438401	14.30954845655164	1.06332153718267
H	10.15049091900716	14.98462816562397	1.03768713333067
C	5.21998921472833	15.97063878233703	1.72824945355964
H	5.89510025197152	15.69128938667743	0.90942463639939
H	5.54979764994655	16.95014651781766	2.11316491375677
H	4.21119929970390	16.11164922314855	1.30860649751197
C	6.07152639887523	8.90345314168174	5.58766175322274
H	5.52336492759038	8.26964789932951	4.87867232596814
H	6.67567916598793	8.23942049876483	6.22856464970752
H	6.76026658794776	9.53657765342405	5.01201090167733
C	8.57601766014053	17.11786611386254	2.02888148432516

H 9.58581517273581 17.11033267514446 1.58719339952044

H 8.51942114241386 17.99478982298300 2.69476884904457

H 7.85560367421806 17.26130285964297 1.20940471336831

C 6.24960843090851 10.13839463185633 0.85397668017088

H 6.49199272078390 10.04078941740772 -0.21701316875134

H 5.37076698321282 9.50198155799576 1.04301379360380

H 7.09203411694515 9.73580736637681 1.43449074697565

C 4.75257917887672 12.12063011006758 0.42470496835073

H 4.43712865758157 13.11269075273899 0.77780326301270

H 3.88704002513298 11.44254032460974 0.49325441490852

H 5.03252793523065 12.20766223084501 -0.63801500364544

Optimized coordinates for **3** (UKS S=0). Final Gibbs free energy: -5720.41146216 Eh.

P 11.17057794740404 11.24211190008072 4.42176268754960

N 11.86384021538733 13.60073935576343 5.68658273379324

N 9.88194188405593 13.91496082486795 7.28635874336913

C 12.44016634331301 12.38452044204999 3.68830927274857

H 13.32218392297926 11.89864084877382 3.24850435351806

H 11.92402169407028 12.92098513655657 2.87792843782385

C 8.82403493430892 13.86118795338668 8.23554730147915

C 12.07788807025324 14.50526031267870 6.71746154944526

C 14.05702803269463 14.00770849530508 4.80818730174958

H 14.80771458470392 13.79997534597472 4.04467031580554

C 12.82142762867285 13.37541786308787 4.75988967021766

C 13.33420422663219 15.16449079550671 6.80811484503560

H 13.52161237851815 15.85780772877146 7.62870163624977

C 10.38206587639179 10.35681412692572 2.95256027940943

C 8.94702759328201 13.02035189482428 9.37011166661081

C 14.31004173972079 14.92068367938789 5.86450837800067

H 15.27660650241489 15.42567700751587 5.93064669100357

C 7.50212372634867 15.52185926271534 6.82639376203241

H 8.12195192147187 15.06070067267438 6.04720937565077

C 9.89238326302180 11.46074241049998 2.00109336940829

H 9.29091702844766 12.22154884587336 2.51659463063357

H 9.25665295068470 11.01576713171244 1.22169985780093

H 10.71877627163985 11.97000350597946 1.48718427119850

C 12.23075441051739 10.02570643775824 5.43897652650388

C	11.37607029409340	8.80679151128957	5.82539767840657
H	10.41622695541203	9.10834761517231	6.27102657817947
H	11.91694349603706	8.20479347031696	6.57468642475363
H	11.16981388821013	8.15308595605782	4.96681322127726
C	12.64585827767754	10.75052811778217	6.73572520278796
H	13.34946048260604	11.57061805266683	6.54467669433723
H	13.14241817092841	10.02791639962431	7.40526863741038
H	11.78150345706850	11.17138258195751	7.26287222814976
C	7.66635571227587	14.65473539169721	8.06168925047208
C	6.83038337222790	13.87676528767445	10.21308050496721
H	6.06282189497324	13.89656061747815	10.99134582305722
C	7.94569334223743	13.05064641731295	10.34775727943007
H	8.03526159113595	12.41148523164092	11.22875434114511
C	13.51582443849428	9.56305243745324	4.73343494458100
H	13.32595515489144	9.03912271880902	3.78934450840993
H	14.05954819930277	8.86667856663664	5.39476201713302
H	14.19180892885088	10.40644947757901	4.53093828310232
C	10.09886147354675	12.03256461097759	9.48211995303670
H	10.92626403911356	12.41580450455777	8.86864097185908
C	10.97892567094438	14.63763138755569	7.60479786944148
C	6.05667101748205	15.56370121737874	6.31975849102928
H	5.64814616324948	14.54866905041018	6.19374344414090
H	5.99735864916186	16.06151814512068	5.34178521210987
H	5.38815993020765	16.10893378042514	7.00504638488702
C	11.31687059905998	9.41761957876935	2.17805404875997
H	12.24376797268645	9.92286700418122	1.86568041902327
H	10.80653450243366	9.07050194752770	1.26328945700535
H	11.58823706816105	8.52445895661073	2.75789256209592
C	6.68927987994249	14.65588869323591	9.06696763942805
H	5.80302508367983	15.28267096480504	8.95032670082742
C	11.06569583059408	15.46697720694275	8.85634468889494
H	10.08361546924937	15.86755493650488	9.14082361410835
H	11.75667477669857	16.31284130705642	8.73334702011809
H	11.42511176156488	14.87023319624761	9.71463705462872
C	9.14159304188739	9.57818345687831	3.43586920390326
H	9.39232074806777	8.75250507511324	4.11229386354510
H	8.62922448341763	9.14757696599387	2.55980260282923
H	8.42550274598222	10.23343752859767	3.94700474533604

C	8.04662862725174	16.93932420867555	7.06415817164729
H	7.52237421291960	17.42453654806994	7.90435324054767
H	7.90629087901807	17.56714707499511	6.17010811476406
H	9.12104616069722	16.92112054556294	7.29790728829297
C	9.66663716282386	10.68383764103759	8.88051290888117
H	8.82665699643232	10.25656780503818	9.45221062756489
H	10.49356088316185	9.95588961455920	8.89365918206057
H	9.33342374704842	10.80819215727516	7.83812858095967
C	10.63378057573796	11.85768678300562	10.90775338235305
H	10.89871363219753	12.82709286228253	11.35884210462146
H	11.53606511188064	11.22491442971877	10.90157015295679
H	9.90007689980014	11.36935713324965	11.56952346618537
Fe	10.11414306500938	12.74549084459456	5.68975549087141
N	8.38193750139172	12.40630847368101	5.28243902863458
N	7.34379715516272	12.40596508262643	4.83234485769974
Fe	5.61102327563556	12.74259949682000	4.42519642550201
P	4.55681466011585	11.23655211591702	5.69194001259682
N	3.86035074178392	13.59587407654674	4.43022674823561
N	5.84076476596462	13.91376366378708	2.82949894762466
C	3.28695455145378	12.37714005640753	6.42775415716465
H	2.40587569307145	11.89017674173841	6.86823943604494
H	3.80367773353157	12.91341799552443	7.23789464580383
C	6.89803376098144	13.86217885511857	1.87944582592804
C	3.64459485513816	14.50122468070948	3.40043076504611
C	1.66732909486530	13.99946924873928	5.31059458345985
H	0.91742073771296	13.79016768871649	6.07444517313579
C	2.90367010069798	13.36855940117254	5.35740615673799
C	2.38744494408834	15.15908828999855	3.31131949616385
H	2.19862979684555	15.85300048831741	2.49155961703796
C	5.34697190350139	10.34996818314447	7.15946094168955
C	6.77528483039436	13.02204256937718	0.74431728666547
C	1.41252857759154	14.91318182694448	4.25532479290479
H	0.44534267850782	15.41713279247130	4.19035470066742
C	8.21944422646659	15.52289301303353	3.28914253310479
H	7.59935707322690	15.06132331348043	4.06786755348429
C	5.83509298956977	11.45302975864127	8.11272077486539
H	6.43449471947391	12.21620588463802	7.59827979838122
H	6.47234052352411	11.00787828231620	8.89076695616998

H	5.00794568906367	11.95943479390993	8.62823952121397
C	3.49682838392479	10.02054035951141	4.67400179912897
C	4.35195677277714	8.80238208979347	4.28618648696792
H	5.31113579864724	9.10484571639920	3.83974848501649
H	3.81076051882630	8.20040812581059	3.53710927778584
H	4.55952875829390	8.14837624360177	5.14421529491631
C	3.08072563855235	10.74609246070401	3.37799827845603
H	2.37650891690311	11.56543480393531	3.56992287012290
H	2.58456175109791	10.02361726985431	2.70801012862403
H	3.94457217115226	11.16809242533931	2.85095110357176
C	8.05483260554550	14.65704011555330	2.05301728720161
C	8.88999099661288	13.88211020693490	-0.09978406384653
H	9.65685746038781	13.90361093614674	-0.87868672659888
C	7.77577549053865	13.05445042258886	-0.23411509180421
H	7.68636945750869	12.41582696991293	-1.11551792994157
C	2.21230760021613	9.55679871230932	5.37984052745042
H	2.40281577536206	9.03272086705371	6.32371755677589
H	1.66878133628482	8.86029650478676	4.71848553910802
H	1.53590328531843	10.39975180180807	5.58279637873731
C	5.62506015869387	12.03234369874885	0.63275853855137
H	4.79745762713570	12.41384712945851	1.24705246106818
C	4.74284825956569	14.63566596543784	2.51252605097018
C	9.66496051768137	15.56321014921923	3.79577162800797
H	10.07256138475142	14.54770124469736	3.92112387806727
H	9.72479557463261	16.06040339927770	4.77402368130143
H	10.33396957131966	16.10816448414345	3.11074947656088
C	4.41387445794919	9.40798940191686	7.93266595535057
H	3.48653062229054	9.91142834999821	8.24662237950835
H	4.92525115531385	9.05979415260993	8.84644179043579
H	4.14327131423690	8.51559151979307	7.35130169839177
C	9.03101204644857	14.66041702877174	1.04687354660250
H	9.91659125381375	15.28820397428877	1.16337130233269
C	4.65461888999152	15.46609337754378	1.26181992554173
H	5.63576002579523	15.86988882594377	0.97859793013901
H	3.96103882797756	16.30976259510839	1.38508667410598
H	4.29783069025929	14.86917135537788	0.40256523213298
C	6.58851209949838	9.57412054221308	6.67438635189246
H	6.33888668617614	8.74953875706991	5.99620060424392

H	7.10159179877026	9.14237639386249	7.54948682222042
H	7.30359383311227	10.23148236751687	6.16453774104212
C	7.67587441646939	16.94093101201712	3.05275228932598
H	8.20047085287148	17.42667336211530	2.21307758198150
H	7.81652331478498	17.56777721543513	3.94743750500509
H	6.60145202469669	16.92364575526712	2.81889757448744
C	6.06025752900056	10.68414328968309	1.23341437861994
H	6.90083109491868	10.25894676242972	0.66104077702578
H	5.23480213238135	9.95453029881340	1.22027878055166
H	6.39377077198403	10.80860201033451	2.27569157734087
C	5.08930703130367	11.85711249458192	-0.79251535052814
H	4.82231597936747	12.82623505254675	-1.24300679274970
H	4.18813192654573	11.22276611625401	-0.78584877870579
H	5.82332116807904	11.37032811891985	-1.45507908181319

Optimized coordinates for **3** (UKS S=1). Final Gibbs free energy: -5720.42199008 Eh.

P	11.18187654714067	10.88757938625212	4.57001976649148
N	11.51485689602743	13.64011778099662	5.33713815580292
N	9.94634015381101	13.66671227214826	7.41742948124655
C	11.91073475853724	12.21723049783365	3.46518853049584
H	12.70294402406189	11.85324730448791	2.79684740866768
H	11.07508466843745	12.56848877878568	2.84011703741767
C	8.97584055008755	13.65682854499464	8.45081329946264
C	11.83967171449869	14.54849316633043	6.32430169960444
C	13.56860861012261	14.04148382094317	4.18343666761086
H	14.24139858029387	13.80358098218029	3.35793522145689
C	12.37258204010695	13.35838046263142	4.33574125875205
C	13.04961968707710	15.28672212146008	6.20189263655834
H	13.31613024215422	16.02165449931908	6.96305675167198
C	10.40934813235178	9.66092884550691	3.35290805205545
C	9.12734235407989	12.73450202940374	9.51822184727591
C	13.89311070910986	15.04729612724696	5.13583321826235
H	14.82588996566650	15.60788754162339	5.03833948773595
C	7.60822157691972	15.46381799678971	7.23985588549237
H	8.29941603626321	15.16453879935937	6.44207434414927
C	9.55346846991377	10.48581078144132	2.37323784529333
H	8.89637724710064	11.20181120404023	2.88554994292493

H	8.91212157514198	9.80260577399717	1.79419114374502
H	10.16299889892810	11.04803944153479	1.65411492609152
C	12.69498766375036	10.13216772685558	5.43147553450226
C	12.31266958132981	8.75919557556756	6.01062950377050
H	11.39955825380913	8.81917770784833	6.62197145707729
H	13.12670551896214	8.40189361000022	6.66313600336688
H	12.15808988054443	7.99998058903414	5.23184388687529
C	13.04663618602346	11.06044030067166	6.61314820556624
H	13.41554507693336	12.03815364363749	6.27997072000521
H	13.84093011802195	10.58763392172273	7.21516159360664
H	12.17779057472623	11.23473357234928	7.26179399100856
C	7.85402358568164	14.52275927159560	8.41196049196131
C	7.11260882109714	13.62470810818577	10.55457507619841
H	6.39641692119998	13.62450463446905	11.38050776402971
C	8.18630986849777	12.73321034642406	10.55422998206979
H	8.29938132862163	12.03066078067567	11.38284280041082
C	13.93089067200206	10.00350504549506	4.52781893138118
H	13.75046960378897	9.38123310324478	3.64191406117405
H	14.75352936449493	9.53790519108020	5.09777582150996
H	14.28570091966067	10.98897000958052	4.19314471054777
C	10.27275676340763	11.73324228819827	9.50334998269619
H	11.07047490283301	12.17014175935646	8.88563020783588
C	10.93362713922015	14.58968061414485	7.42129180722285
C	6.17907076133845	15.35028610651316	6.68505843832876
H	5.95795788162506	14.32767274616733	6.34027111457142
H	6.03958085706309	16.02424028816823	5.82705843952885
H	5.41922132821522	15.61757572080207	7.43683843859985
C	11.39800882715668	8.81502134669219	2.53761409386225
H	12.09638727268645	9.44379283194189	1.96416541240496
H	10.84014478133471	8.19910804133516	1.81118714839741
H	11.98730011762785	8.12848766422370	3.16071070235507
C	6.95069847385800	14.50075011933196	9.48419623006438
H	6.09521097902904	15.18094463724264	9.47400818531980
C	11.16751684421048	15.53983954696686	8.56597427754113
H	10.31437720220142	15.56030202861074	9.25503823390757
H	11.34920757747327	16.56844747663628	8.21567469865721
H	12.05581490824597	15.23934887478439	9.15141665410805
C	9.45639356859756	8.75460115150109	4.15799518529990

H	9.98508126092747	8.11080361155443	4.87309496455835
H	8.89848274601136	8.09940687135766	3.46726026969264
H	8.72601203077705	9.35630373221996	4.72031029021678
C	7.92094228253833	16.92315950997950	7.60672708480131
H	7.26613519380479	17.27525229190514	8.42125991155156
H	7.76331691192160	17.58512912327379	6.73963578613016
H	8.96173749375080	17.04116897078438	7.93829315237131
C	9.82333581473335	10.43321023930307	8.81410816789770
H	8.99157497575650	9.96581082532484	9.36706503662379
H	10.65048941166624	9.70632032188147	8.75845360164864
H	9.48036309667295	10.63105928021279	7.78567715502077
C	10.86500022020575	11.44854399204989	10.88812756481457
H	11.15505602383766	12.38028478220352	11.39908157781302
H	11.76195128131154	10.81472998430595	10.79563317122538
H	10.15674695740344	10.91346176094135	11.54177406174658
Fe	9.90756604555765	12.49977538828648	5.75907425699801
N	8.19509331056166	12.48929753014372	5.00339084130231
N	7.12617374287236	12.51404028181390	4.63727057756382
Fe	5.42573276804728	12.93351137814014	4.28855171384686
P	4.31101309400853	11.48832852556982	5.57040307952806
N	3.68713135305011	13.79438832177490	4.16460524160422
N	5.76994271339708	14.07015790978321	2.71185846459903
C	2.97050548883593	12.63838809576719	6.15224118296482
H	2.06531599449776	12.15035670201149	6.53865253880023
H	3.41091411876550	13.21583545665601	6.97919078801567
C	6.96687995010349	13.97141726137685	1.95575920081320
C	3.53532767746123	14.63454993418918	3.06859410418722
C	1.41595399337717	14.17106436830498	4.82688309944021
H	0.60605500938804	13.97458500924754	5.53053530458641
C	2.65856283146259	13.57855159702800	5.01326758710079
C	2.27918319835096	15.25581279805777	2.84051688060476
H	2.14946731361754	15.90358968209116	1.97233321992115
C	5.01878701693752	10.69888289552569	7.13437397286029
C	7.04223125585609	13.05596323619033	0.87934185567593
C	1.23281415890039	15.02935418605825	3.71127137546721
H	0.26382613360840	15.50466642799877	3.54167928086612
C	8.01117501373271	15.71716126746330	3.49494812876566
H	7.40979044115667	15.22375899638266	4.27163186635279

C	5.49871818412475	11.85705948719473	8.02540041205915
H	6.19057424235970	12.52137875746283	7.49405861793654
H	6.04574380345303	11.45281206090652	8.89032887849025
H	4.67430849051606	12.46691731092799	8.42082785848945
C	3.36244248016338	10.20169827903418	4.53468762843741
C	4.25890661420163	8.97689559592537	4.28765469630315
H	5.25782494396133	9.26890110568439	3.92881824275704
H	3.80002623083773	8.34058539931583	3.51265286461533
H	4.38011135158687	8.35904346523348	5.18788116399659
C	3.06411189674213	10.85361887768154	3.16850797831299
H	2.36171163729418	11.69268383073290	3.25200644050890
H	2.61368435109639	10.09844264701701	2.50252364250650
H	3.97800351948374	11.22847207687841	2.68960268832345
C	8.10423632326342	14.72512370758363	2.34397491754522
C	9.37833058618395	13.65461650628384	0.56194122013775
H	10.31982431780301	13.53398179104757	0.01949961161041
C	8.25545818352328	12.91815561538199	0.19191590632030
H	8.32562291865374	12.20936859634218	-0.63719077426952
C	2.02640910928354	9.75812308519861	5.15144883399094
H	2.14165478252120	9.30784508400473	6.14505706576995
H	1.55884908334869	9.00226630099165	4.49735560859383
H	1.31879881119360	10.59621978240686	5.23191184675831
C	5.85784206990482	12.17683842802999	0.50403538012613
H	4.97848850416525	12.55609557381949	1.04182734904514
C	4.69638363564092	14.74268143429734	2.25808957781832
C	9.36747889458787	16.07871418630560	4.10496337689325
H	9.92436462726997	15.18562474808428	4.41610188426151
H	9.22636365792283	16.71216027157836	4.99249610304493
H	9.99395020297299	16.65031394111418	3.40083209319906
C	4.02515064478288	9.83410067137111	7.92181806268717
H	3.09549911175121	10.37719924196859	8.15226387944077
H	4.48041367263138	9.53840392200238	8.88233542977823
H	3.76195807580786	8.90988510767878	7.38843899039481
C	9.29928121779377	14.54323901164709	1.63536597541374
H	10.18706177480096	15.10604597746737	1.92539618230884
C	4.68989338803451	15.51285148977202	0.96718288598622
H	5.70960241958811	15.65940520269827	0.58879448055607
H	4.22417754115509	16.50355628100383	1.09017874050014

H 4.11744069037603 14.98324219336769 0.18442244963087

C 6.27015135796082 9.87186984047660 6.77474514091222

H 6.04027283295101 8.97388927244172 6.19044040426449

H 6.75942038341870 9.54593743214007 7.70715395534004

H 7.00023402740238 10.46992189864671 6.21405302403540

C 7.25826472930995 16.99539229618112 3.08773600216999

H 7.78172204748564 17.50962796589937 2.26448226827156

H 7.20137029828304 17.69308672732604 3.93969686639355

H 6.23171163936932 16.78336077573264 2.76346394421311

C 6.09078834264356 10.73324875140461 0.97809254931813

H 6.94962491809113 10.28091502444993 0.45643155660183

H 5.20616585658111 10.10551185713664 0.78372580262195

H 6.30269652069770 10.70534883345498 2.05826683713805

C 5.53384905651971 12.22619573468251 -0.99527379486747

H 5.37995514425924 13.26202364819662 -1.33578943795991

H 4.61759449463970 11.65288553598095 -1.21232298707001

H 6.34337047163325 11.79183827287840 -1.60431907367124

Optimized coordinates for **3** (UKS BS(1,1)). Final Gibbs free energy: -5720.42015904 Eh.

Fe 10.07789981971578 12.88336576517664 5.98730491928753

Fe 5.64745633507237 12.88284207531331 4.12777293413007

N 8.40331095572209 13.20028282661252 5.22416036738495

N 7.32316566006738 13.19960097686694 4.88858181720874

P 11.08949975510973 11.22209964661670 4.60743505009407

N 11.95108066066824 13.67433360563763 5.90140494641165

N 10.16113690311875 13.79103892152015 7.78609404323534

C 12.32344569185889 12.43151160614435 3.88918843049772

H 13.11653219890160 11.95349863203024 3.29794462972147

H 11.73972941093911 13.06842101054350 3.20857533627138

C 9.06614010078639 13.86859575885001 8.68096145091733

C 12.32669404563191 14.33968074864841 7.05456100278472

C 14.21297706864746 13.64030375759303 5.11620252912090

H 14.93536165244269 13.32503241042212 4.36165992628941

C 12.87765179884640 13.29174794914002 4.99706707072490

C 13.68056893141516 14.74950923515387 7.20413090974423

H 13.98415678265590 15.29585978761844 8.09828975718828

C 10.21759623931097 10.51020684886226 3.08767794071209

C	8.98866562668076	12.94854876534040	9.75517390082433
C	14.60909750018987	14.41738307421722	6.23979756526295
H	15.65266049392293	14.72045238546751	6.35325827736792
C	8.10770190678297	15.80847876096216	7.31021781192392
H	8.75363564211950	15.35679877897367	6.54480293631293
C	9.80842996237224	11.70134560579562	2.20061790077641
H	9.32242232049358	12.50743785524292	2.76869317320433
H	9.08437294518466	11.36243809319693	1.44584956199568
H	10.66083614127644	12.13080227896815	1.65711834039210
C	12.15605205759537	9.92506209044660	5.48350402598290
C	11.28256227334898	8.69853054718664	5.79866366903500
H	10.36252091492795	8.98651797792223	6.33107045834175
H	11.84348217587888	8.01070304402088	6.45328008301796
H	11.00017053823167	8.13785922275223	4.89670944550116
C	12.59761611330502	10.55119862567527	6.82405505332765
H	13.28999524138213	11.39066604381984	6.68080617858734
H	13.11520498160627	9.78331846555613	7.42343799757392
H	11.73876489891236	10.91865088623813	7.40127737906065
C	8.03518741293223	14.81782321991058	8.46522996918413
C	6.88212596131781	13.95872344089894	10.43508924738961
H	6.03368494606855	14.00065332790300	11.12326263164468
C	7.89168159890133	13.01429916071533	10.62256000357240
H	7.82114830044415	12.31260537286162	11.45701196193381
C	13.41312730575170	9.50414832511657	4.70756860723960
H	13.18511279037270	9.04872474316690	3.73579274051785
H	13.97320955550065	8.75946743664161	5.29896272062766
H	14.08682971992686	10.35744491049085	4.54158531512210
C	10.04711319480897	11.86589532277103	9.91514566972709
H	10.95535509229896	12.21757620697090	9.40526648987299
C	11.30341855284045	14.46208832983472	8.03629464543586
C	6.74283609883558	16.08154108309825	6.66416704812621
H	6.23322709791099	15.15066763274473	6.37389495840601
H	6.86946079393259	16.68540783799111	5.75221998776374
H	6.07267846014545	16.64570310463494	7.33316813027487
C	11.05451086144958	9.53759628562560	2.24588263401935
H	12.00918988520538	9.98469224213948	1.92899778247501
H	10.49525449650293	9.27461230650703	1.33160777597357
H	11.27172167717310	8.60022835405328	2.77707333860285

C	6.95603510792714	14.84330600500859	9.35986694237599
H	6.15746751990796	15.57350482095250	9.21317075227520
C	11.53637680617280	15.21246843601397	9.31994700964631
H	10.58707195822893	15.50657366126601	9.78656743668330
H	12.13590033048071	16.12089127092745	9.15894978181672
H	12.07696437130697	14.58917525738464	10.05679328110693
C	8.92135107920293	9.82725766337795	3.56470814410280
H	9.10089294086868	9.00451030194593	4.26858036743721
H	8.38560120308278	9.41472406494480	2.69458222012691
H	8.25376220875379	10.55075022042682	4.05197603678585
C	8.77604417114955	17.12556756389410	7.73995772677742
H	8.19800246817682	17.61796385786473	8.53997499438846
H	8.84164594941266	17.82291150588073	6.88802860111022
H	9.79569521021753	16.95773624834179	8.11347350820342
C	9.59733719033532	10.58396953858324	9.19367509128403
H	8.67941640869775	10.17949861710824	9.65199982977104
H	10.37553808069073	9.80446567481388	9.24286678154269
H	9.38454551783384	10.78762443941116	8.13288611431347
C	10.41867116884218	11.58001245060125	11.37464240739234
H	10.71390010387628	12.50221023352731	11.89965080666944
H	11.26212483186319	10.87199702571449	11.42408545659328
H	9.58357530409309	11.12805551167485	11.93446627504561
P	4.63628467011146	11.22159238500299	5.50761054772351
N	3.77448479493610	13.67393249479401	4.21428102974321
N	5.56328676316341	13.78954091063514	2.32841173545582
C	3.40272288843283	12.43100823904340	6.22655153171158
H	2.60981037335626	11.95300190331466	6.81803227778921
H	3.98674553273932	13.06778849113943	6.90703753359637
C	6.65781422334662	13.86673973317608	1.43294827184810
C	3.39838230192236	14.33907304962774	3.06113953447764
C	1.51288605750200	13.64016823555362	5.00040853048138
H	0.79078290721198	13.32502875822736	5.75527654014374
C	2.84819041191555	13.29138659165091	5.11895995810471
C	2.04450962283289	14.74913944632997	2.91217006697948
H	1.74057773955727	15.29535541209255	2.01804486555988
C	5.50877774339167	10.50969691733428	7.02698687862178
C	6.73489751247166	12.94615041893847	0.35917501100653
C	1.11638278613165	14.41729829299019	3.87697362771140

H	0.07282346460408	14.72055580751647	3.76398517920752
C	7.61667848784357	15.80725446543487	2.80235358037174
H	6.97107557885781	15.35570588007105	3.56811243514196
C	5.91787421181539	11.70092788653957	7.91392087819743
H	6.40396520969802	12.50694842758666	7.34578319958228
H	6.64181192149766	11.36207641523858	8.66879931615660
H	5.06544727241785	12.13049734682424	8.45728103326664
C	3.56926568549010	9.92456028689982	4.63202546235913
C	4.44269155169074	8.69814832860532	4.31620096725368
H	5.36221043984832	8.98626495611484	3.78297088605825
H	3.88132494463280	8.01017869431199	3.66211710426941
H	4.72596259878810	8.13759056622074	5.21794478512157
C	3.12682356571093	10.55075529299899	3.29179474152998
H	2.43434341668782	11.39005408845656	3.43553710251187
H	2.60904736026744	9.78282729618507	2.69263862534439
H	3.98526322513783	10.91844426951916	2.71411807544032
C	7.68864755497110	14.81633179708765	1.64755527709857
C	8.84062741988549	13.95670280648741	-0.32269614752938
H	9.68861925347498	13.99853784510385	-1.01143025251176
C	7.83126358919032	13.01183603738448	-0.50900176464112
H	7.90154480314013	12.30962112490108	-1.34303443156071
C	2.31267254747169	9.50340411202195	5.40861092213451
H	2.54128595939093	9.04766018279902	6.38008463509655
H	1.75224281179465	8.75890661691526	4.81731035851259
H	1.63904046519663	10.35662029717215	5.57527517878070
C	5.67669464215708	11.86309469110846	0.20043309324160
H	4.76869962185273	12.21467538207176	0.71082408402622
C	4.42105067896519	14.46082180904787	2.07871941526803
C	8.98183503665360	16.08033029025986	3.44778517002235
H	9.49165333787922	15.14944146882381	3.73760757819245
H	8.85559434687158	16.68396108769677	4.35994416863952
H	9.65161214794292	16.64467902292797	2.77855071181557
C	4.67237371519324	9.53688765341139	7.86906863836175
H	3.71773574625991	9.98378577275308	8.18634726574217
H	5.23203117617297	9.27397156517066	8.78311980652644
H	4.45513742581950	8.59950138971840	7.33793436754135
C	8.76720952669414	14.84171676965621	0.75220695965717
H	9.56573517394803	15.57212779514848	0.89811785586174

C 4.18738235878681 15.21077958765449 0.79494104136427

H 5.13643544162314 15.50375978148392 0.32711810218168

H 3.58887107041407 16.11984105503400 0.95613907309549

H 3.64536301558057 14.58761607262983 0.05904057698028

C 6.80505124833764 9.82711977157466 6.54951266987581

H 6.62552937148915 9.00459026326305 5.84537351415933

H 7.34100836848214 9.41435414953035 7.41941341792253

H 7.47243340742250 10.55091229471999 6.06239405528269

C 6.94821633392018 17.12431486927252 2.37272391647119

H 7.52581128761060 17.61644822515101 1.57222054777171

H 6.88325925282419 17.82187429117108 3.22452742655340

H 5.92829755701102 16.95653694436363 1.99991690676407

C 6.12757064900457 10.58168293839954 0.92211783610345

H 7.04514575505858 10.17729102590424 0.46303570478297

H 5.34955850693414 9.80190989581337 0.87414822791141

H 6.34142099924205 10.78593712734692 1.98258696723361

C 5.30417026538527 11.57642447987613 -1.25866717146569

H 5.00822395061050 12.49827771297188 -1.78387301080259

H 4.46093263123280 10.86808756844568 -1.30717267949206

H 6.13901680552384 11.12451606107019 -1.81890736931139

Optimized coordinates for **3** (UKS BS(1,1) CPCM(benzene)). Final Gibbs free energy: -5720.43025237 Eh.

Fe 10.08248758588239 12.88662506253215 5.98143882768344

Fe 5.64472606971887 12.88424447756605 4.13253058218779

N 8.40398320676523 13.19748196667111 5.22396688715536

N 7.32385814035380 13.19565129714008 4.88844162292976

P 11.09204066668092 11.21545875645092 4.60863137102835

N 11.95736714753218 13.67611977108723 5.89623278256213

N 10.16423417595830 13.80304204169016 7.77749130229985

C 12.33585983963003 12.41583384723887 3.89333659337740

H 13.13036842315292 11.93167228574093 3.30950793209990

H 11.75989372510328 13.05156303888707 3.20506550090777

C 9.07003577567446 13.88095355922971 8.67297614341480

C 12.33350737345438 14.34206017600547 7.05065621511494

C 14.22499262582297 13.61803813573744 5.12531023050769

H 14.94935170305061 13.28986839967800 4.37811784942870

C 12.88726967102890 13.27916208441188 4.99975045766204

C 13.69009828488517 14.74367379078357 7.20492786037118

H 13.99343507792680 15.29169310600730 8.09832885508155

C 10.21764201492693 10.50889610842894 3.08919286513006

C 8.99400181478106 12.96184120355347 9.74828404523513

C 14.62144180563708 14.39896346320413 6.24723320505885

H 15.66689229403476 14.69451468595641 6.36474989013082

C 8.10608053811756 15.81438289625832 7.29727423460103

H 8.74743767997641 15.35793957355968 6.53087059958369

C 9.81531265067087 11.70231772180111 2.20189020817388

H 9.32943280095820 12.50907807471848 2.76936987957112

H 9.09239280827548 11.36490527240485 1.44520205757918

H 10.67175095821858 12.12926137998548 1.66260773496225

C 12.14508607143308 9.91372867489684 5.49184053892252

C 11.26117763510635 8.69542100416185 5.81008983561346

H 10.34145567893160 8.99250042940950 6.33818668470301

H 11.81578791794136 8.00710441800786 6.46942811890355

H 10.97798290966304 8.13359026895338 4.90918097035147

C 12.58950812336130 10.54234071895091 6.83023899516257

H 13.28781812231921 11.37638823052652 6.68286672768562

H 13.10085269043131 9.77315954494834 7.43302590665025

H 11.73240823050097 10.91822045215877 7.40481671904296

C 8.03675271289536 14.82767805470448 8.45592148968893

C 6.88531595823049 13.96957676764282 10.42762505414345

H 6.03569579060435 14.00958041882686 11.11470665660220

C 7.89664437823763 13.02652720811374 10.61585734533736

H 7.82625262803059 12.32365391306815 11.44949172763541

C 13.39977111198828 9.47906409239778 4.71930052524351

H 13.16755656510028 9.01740399130372 3.75156279227261

H 13.95367946700802 8.73532628730316 5.31758657597820

H 14.07903594615073 10.32640621620244 4.54517230211962

C 10.05162675120848 11.87844736035590 9.90846010048579

H 10.95735296649487 12.22484645774603 9.39077980373873

C 11.30779138900157 14.47093414947267 8.02927996345386

C 6.73890859167063 16.08740133933084 6.65610164732058

H 6.23092968193042 15.15524056302807 6.36658761202194

H 6.86328419690066 16.69276533988361 5.74471178942149

H 6.07088118255148 16.64953647925086 7.32898196349288

C 11.05294976933257 9.53251406224521 2.24991654117010

H 12.01070352299778 9.97545370524869 1.93661949511740

H 10.49405212610638 9.27228434982409 1.33457910500917

H 11.26314364727280 8.59467411967643 2.78297731965174

C 6.95753910997155 14.85311001631257 9.35079404365829

H 6.15653484967066 15.58054038495069 9.20283926366949

C 11.53856590701373 15.21881591501521 9.31478987215042

H 10.59149366896812 15.56861513013139 9.74693722763546

H 12.19209615080522 16.09111904049761 9.16857871169984

H 12.01705616605763 14.57206450624366 10.07402812528723

C 8.91789152421661 9.83102537454115 3.56391508317307

H 9.09309083896137 9.00690474570144 4.26707915061533

H 8.38334457593894 9.42072651338199 2.69202500438418

H 8.25155254503142 10.55636659410249 4.05012992169415

C 8.77872018509146 17.13223818355240 7.71832181011465

H 8.20527699881185 17.62849990839483 8.51945807690564

H 8.84047453189677 17.82636384824492 6.86327872208577

H 9.80011245756315 16.96379182460291 8.08731261704631

C 9.59435760016309 10.59284658094126 9.19820355652950

H 8.67809071389435 10.19342340328374 9.66404720865443

H 10.37184124442416 9.81275480930184 9.24801965642442

H 9.37605736593878 10.78989753104936 8.13732152770569

C 10.43126373992600 11.60198142865070 11.36793038275191

H 10.73615950179203 12.52687367382441 11.88322273941106

H 11.27125195652958 10.88970428311290 11.41635858645621

H 9.59662983737328 11.15958133367568 11.93600167438768

P 4.63556167019398 11.21421177241005 5.50676405326939

N 3.77067586907952 13.67596872934251 4.22165019412073

N 5.56003179737576 13.80099813971422 2.33649074650867

C 3.39516967877036 12.41620646058529 6.22535935277459

H 2.60127704074143 11.93307062726749 6.81091414345054

H 3.97380799801097 13.05085908765405 6.91242746327799

C 6.65298576418866 13.87894049737748 1.43946816715766

C 3.39269607426276 14.34193527924464 3.06787742524382

C 1.50533374976173 13.62358169361857 4.99948665362093

H 0.78249782880078 13.29762891804882 5.74912201998481

C 2.84256219354626 13.28108635615037 5.12077275926826

C 2.03645184815994 14.74596552504068 2.91695991130195

H 1.73156895174752 15.29375785685404 2.02395282931124

C	5.51075856680327	10.50447288122110	7.02423378261110
C	6.72785484550777	12.95963893838107	0.36429090182465
C	1.10740170926138	14.40506412341820	3.87854934379612
H	0.06237995473546	14.70336398447541	3.76424011928841
C	7.61790954692904	15.81283046408521	2.81343886643569
H	6.97758409286053	15.35622108677413	3.58060125531736
C	5.91441268815936	11.69609125774457	7.91326420172440
H	6.39950131990824	12.50407372648439	7.34677148903782
H	6.63829489522553	11.35723682422820	8.66830694540347
H	5.05881211023730	12.12186111228705	8.45478656293606
C	3.57934019652899	9.91486073914043	4.62382140177123
C	4.46150924628438	8.69603249917497	4.30261720981420
H	5.38016188312676	8.99279940232012	3.77250438323082
H	3.90470952331372	8.00866210386562	3.64412026655459
H	4.74668267998855	8.13325983138438	5.20230782539668
C	3.13328132202622	10.54521343291650	3.28679210118956
H	2.43559394789834	11.37943301950348	3.43604622103137
H	2.62057350265893	9.77693909349788	2.68400604253467
H	3.98972537813194	10.92117248078956	2.71125158341022
C	7.68634657949514	14.82591884375671	1.65492285755332
C	8.83604409469532	13.96693815169578	-0.31748207283611
H	9.68503044421368	14.00660923705551	-1.00534939991929
C	7.82430236703677	13.02411975766010	-0.50444106729396
H	7.89378824065607	12.32094920155104	-1.33792982270644
C	2.32548243299041	9.48082638837207	5.39801956408820
H	2.55876726655069	9.01687392361594	6.36437644911957
H	1.76900920447264	8.73929077410765	4.79937442630430
H	1.64812770507262	10.32907817175501	5.57515612265174
C	5.67001868186887	11.87632789813009	0.20542600329141
H	4.76468345053565	12.22308075978677	0.72355530319222
C	4.41632590604107	14.46942615227525	2.08687924597704
C	8.98558023015853	16.08650915012493	3.45325723035004
H	9.49431149706607	15.15447140195842	3.74179242386379
H	8.86173634588726	16.69135166171456	4.36507391927459
H	9.65256240632224	16.64931867711144	2.77991130290940
C	4.67575204488940	9.52713880839930	7.86269836585566
H	3.71871000343241	9.97020559812444	8.17797442582615
H	5.23553889156462	9.26488330914186	8.77690910031764

H 4.46422227497104 8.59037949770127 7.32827520758822

C 8.76480488367727 14.85090372382317 0.75911293375925

H 9.56585114655972 15.57849904906282 0.90597672836306

C 4.18297895454336 15.21748095027900 0.80189251491526

H 5.12966678541370 15.56200078083782 0.36475643080146

H 3.53462286594688 16.09325899965851 0.95067141201991

H 3.69719147554141 14.57261105161642 0.04570358716346

C 6.80953528861392 9.82664647552018 6.54680450732298

H 6.63290684654060 9.00455255308371 5.84156648747760

H 7.34450263343920 9.41359227313945 7.41716675284395

H 7.47603694536895 10.55271880982547 6.06187053497187

C 6.94426548567129 17.13033943761403 2.39292072885191

H 7.51690821073644 17.62694265351810 1.59142458412327

H 6.88272893892794 17.82440096672005 3.24803466446880

H 5.92271573050683 16.96141722116768 2.02461131209917

C 6.12798772734555 10.59122580081310 0.91615295979531

H 7.04403443665303 10.19185274071826 0.44981506379256

H 5.35064964305568 9.81091194077860 0.86747742458371

H 6.34714309346632 10.78901733185090 1.97674326466601

C 5.28924933572818 11.59908727865010 -1.25357755408325

H 4.98371860497847 12.52370901578763 -1.76897531250261

H 4.44938919897085 10.88659154554830 -1.30097470329899

H 6.12350173450439 11.15662626705927 -1.82215113336399

Optimized coordinates for **4** (UKS BS(4,2)). Final Gibbs free energy: -4346.98060017 Eh.

Fe 2.80976913600380 2.53985398384580 4.90166431196734

P 2.70907767138478 1.74959393576555 10.01361719398478

P 0.06922381511293 -1.88069281216598 5.28977724068276

N 3.95908255566953 1.69964921926958 3.49843399063758

N 3.89782484020999 1.20707864367621 6.09355292347997

N 2.19893386480427 4.42322945749470 5.17364590801497

N 0.81756980798776 2.17796629390602 5.46089375239994

C 4.63170356938684 0.33704837289238 5.30464346386306

C 5.30035873080263 -0.76408724988759 5.89952050764137

H 5.87299580630350 -1.44986489744844 5.27545415765767

C 5.20710720824770 -0.96151919919341 7.26023169018672

H 5.70675799148672 -1.81111769046963 7.73123961749466

C	4.43405866032571	-0.07484981508917	8.04114459255753
H	4.30699028358165	-0.23110309722275	9.11105909851994
C	3.80625373366387	1.00306221904082	7.42817199078760
C	2.97130475755933	2.02128844710996	8.17435314419386
H	1.97286452454284	2.01750412261790	7.71425258253581
H	3.37473247610071	3.02418112894229	7.96379304993716
C	4.08348750765519	2.83143115937752	10.79885961520340
C	5.41877246158922	2.36800808038084	10.18687770899825
H	5.59024139332115	1.29295679015470	10.34483471822480
H	6.24753697380420	2.91257665495777	10.67157485889034
H	5.48073742011461	2.56241765299382	9.10662787374331
C	4.12381568568644	2.51284979714255	12.30494805868779
H	3.22203442383040	2.85508036939165	12.83034113117739
H	4.98913590327000	3.01642412470262	12.77032019146199
H	4.22566907272109	1.43026262077627	12.47936923712087
C	3.94009842616297	4.34210107644817	10.58972584353380
H	3.79466066173471	4.59848613451506	9.53204960371141
H	4.85430723028352	4.85818175878014	10.93331564757250
H	3.09892696315356	4.76023314697245	11.15953127632238
C	1.01923462931091	2.63110684905493	10.22119594568237
C	0.73923423149930	2.83643558710437	11.71790852083556
H	0.90828104272021	1.91317048761673	12.29482069347993
H	-0.31529701910882	3.13043270389030	11.85849979324530
H	1.36094103168886	3.63267136997348	12.15181859808447
C	0.83823590488947	3.96051579084296	9.47327889903420
H	1.45837389421338	4.76524404811245	9.88501655945404
H	-0.21499398746291	4.28254770392406	9.55332559153563
H	1.06089604161590	3.86724647714289	8.40256418050493
C	-0.00462480979725	1.61680641948935	9.67440042030721
H	0.12225949487723	1.43102463555851	8.59948061958518
H	-1.02615613624782	2.01042231871332	9.81059594776628
H	0.06631352591257	0.65127258620563	10.19698617717230
C	4.66927745881883	0.62940180451132	3.90832750759031
C	5.44122413061998	-0.24440903253532	2.95660503924456
H	4.92910621195820	-1.21150318123334	2.80584644890402
H	6.45296780005702	-0.47010142785166	3.32776375629929
H	5.54122957811010	0.23372723480473	1.97455633048186
C	4.16163605939951	2.22211902874734	2.18946426481861

C	5.16852185676777	3.19994898582373	1.99477568491370
C	5.99051064428149	3.72022932754673	3.16642931915326
H	5.35135101562526	3.64066082178228	4.05831271357026
C	6.38441305825800	5.19286450532542	3.01580395771075
H	7.14032535953447	5.33566170576506	2.22594195837179
H	6.82417036041128	5.56413642435612	3.95413632281828
H	5.51557877781428	5.82207296927318	2.78278927503195
C	7.23787790968482	2.85828279747421	3.42021098373457
H	6.97855094796755	1.81192329703812	3.62526226556627
H	7.80016105185119	3.24196065833731	4.28745704237974
H	7.90785195940494	2.87788403790420	2.54430273328267
C	5.40329945688875	3.66222789867453	0.69298464393437
H	6.18241857493670	4.40664612874690	0.52071570065816
C	4.65515526824700	3.19395517452164	-0.38650552902449
H	4.85379084889554	3.56662733213184	-1.39442553771981
C	3.63692079231880	2.26702104460992	-0.17205527039347
H	3.04078494490896	1.92076761204294	-1.01825398376331
C	3.37143509632087	1.76761961707730	1.10989363193708
C	2.24946192157524	0.76623999122776	1.33879393701085
H	2.49872403883357	0.21195147793705	2.25473791914978
C	0.91932502897249	1.49397944697987	1.59710590979270
H	0.98002696471643	2.13273582837990	2.49054726750559
H	0.09783994219072	0.77688620226785	1.75553061297443
H	0.65009551162448	2.13258487426219	0.74051825993462
C	2.11631183227771	-0.25736274476945	0.20538823715539
H	1.75821273945907	0.20531297698045	-0.72825214633458
H	1.39260302601952	-1.03928267354504	0.47699712785051
H	3.08140058809147	-0.74350987881279	-0.00752702164565
C	0.21673804529777	3.35016424968694	5.88903754283925
C	-1.09800108315403	3.31974512742277	6.42267730150230
H	-1.55323938647788	4.23880308936140	6.78993409643778
C	-1.78828561197965	2.12793407268580	6.46064361998903
H	-2.80728386945175	2.09105718767973	6.85257749312014
C	-1.16476916193718	0.94829075018131	5.99975828854830
H	-1.68491515636475	-0.00874200196070	6.01016203447451
C	0.14236929606457	1.00644363316059	5.52788773261025
C	0.89538833521513	-0.21107354785288	5.03330847034065
H	1.90937471694498	-0.19964078638428	5.45955213504917

H	1.03533685245106	-0.08677341344533	3.94994839241661
C	0.81991314360899	-2.84203682151709	3.81347659493591
C	-0.02621628821777	-2.38887545672487	2.60682810467806
H	-1.09300762271296	-2.61122025820888	2.76082918335155
H	0.30773671569242	-2.91469203821024	1.69570051356988
H	0.06516193936176	-1.30964231500055	2.41643308267185
C	0.58240316441452	-4.34670861735823	4.01648521461977
H	1.21308326045331	-4.76370511274835	4.81395396218944
H	0.82692235257628	-4.88870161826777	3.08656413645753
H	-0.47003173765385	-4.56122685063285	4.26185848553840
C	2.30642342329958	-2.58772576336435	3.51504489625803
H	2.53471479844897	-1.51891184332161	3.40794168626230
H	2.58211913057101	-3.07703523166558	2.56407086567672
H	2.96373077908861	-2.98872272645985	4.29522496941031
C	0.86571635096833	-2.49858307486778	6.91743452093327
C	0.02813021639732	-3.69653729286975	7.40275843548159
H	-1.03349844026826	-3.42416934504295	7.50878888611891
H	0.39495289012609	-4.03234944620067	8.38853865452507
H	0.08784433645077	-4.55361944099972	6.71712504014854
C	2.34301180979218	-2.89126573476605	6.83583833684310
H	2.49833714337249	-3.79659999143850	6.23215037774621
H	2.72894268310125	-3.10286768679838	7.84848101272408
H	2.96202694283361	-2.08716414993296	6.41965965090048
C	0.71040149772745	-1.35953465097678	7.93905088212085
H	1.34744576381318	-0.49834164748459	7.69823565049616
H	1.01778674205481	-1.71275433552877	8.93775155817506
H	-0.32951029525850	-1.00877497369356	8.01133904173404
C	0.97331400980885	4.54911946710400	5.72432780805860
C	0.41778563354528	5.87770711529925	6.16374120492343
H	0.40159235585261	5.95306229656587	7.26562446067608
H	-0.61572977233192	6.03168565184671	5.81661726564280
H	1.02674810964518	6.70608409350962	5.78198049648760
C	2.85834112770613	5.58151558419257	4.67110681876746
C	2.57625353916367	5.99929827006712	3.34611790383828
C	1.65539802901352	5.18726481418351	2.44283603410923
H	1.77246173496948	4.13498142084536	2.74316086582591
C	2.03636620678623	5.28125242743746	0.96199299965327
H	1.79483202625568	6.27088387450656	0.54019454009414

H 1.47339849836687 4.53482419993537 0.38166901169200

H 3.10523539449181 5.08764936186824 0.80531976621362

C 0.17312201980939 5.55229126227448 2.62703185227676

H -0.17662682265327 5.35166497625556 3.64675869669489

H -0.45309069154645 4.96212982595690 1.93827815220443

H 0.00579765423576 6.62023585925413 2.40892286336808

C 3.16642888236286 7.18267090252104 2.88199544280546

H 2.95288098227569 7.52740802029678 1.86919146636526

C 4.02942738185138 7.92380570293064 3.68712386762641

H 4.47734412682516 8.84556021225049 3.30778121399916

C 4.33851957064497 7.47215527600866 4.96860668228452

H 5.03385729909108 8.04681748919161 5.58211213267052

C 3.76598196137042 6.30069773505611 5.48241523117014

C 4.12855920470284 5.80159147729508 6.87452253852411

H 3.24281005143332 5.28178032242614 7.27420610383170

C 5.26911616024973 4.77184929500391 6.79604893534437

H 4.98304422925773 3.89526380891092 6.19696659676545

H 5.54785571502826 4.41606626566223 7.80007797107950

H 6.16363591760264 5.21966209487370 6.33510895524894

C 4.48630595105143 6.92870613658653 7.85045170715054

H 5.43075331271338 7.42450026302551 7.57475563677165

H 4.62183193371944 6.52785282807078 8.86557004181744

H 3.69883643259492 7.69788405924221 7.88634059434778

Optimized coordinates for **5** (UKS S=3/2). Final Gibbs free energy: -5380.76307837 Eh.

Fe 6.70843594513093 1.71714942774412 2.77032765392709

P 7.78009270125629 -0.48721583252506 3.20913367611475

N 5.15690788095048 2.68285504015168 3.65887350299966

N 5.15967312748339 0.41797836661630 2.42610414297467

C 3.92568825570428 0.97540369669706 2.63038285479318

C 2.76992567253216 0.25285064934012 2.26914201161460

H 1.79211083913698 0.69739110801373 2.38643633129556

C 2.90133577394434 -1.02915353959367 1.79102105224692

H 2.02349964728040 -1.59897755890198 1.51530120854565

C 4.17960199797602 -1.61463955974551 1.69007870793017

H 4.29985693269042 -2.63783410140250 1.36174488516393

C 5.28266276038263 -0.85428673039512 2.01962101399329

C	6.69201780099972	-1.38612511323991	1.98889653158351
H	7.13542041095114	-1.18407483962670	1.01041434507842
H	6.69866628373537	-2.46708009522973	2.12986824118901
C	9.51337904089710	-0.95024984886733	2.59712525169925
C	9.80626816981800	-0.05359793942252	1.38435778720239
H	9.07548216980305	-0.17296790002795	0.58499091646093
H	10.78713821480871	-0.32135736322339	0.98205755608814
H	9.81708833230197	1.00096043721814	1.64940649169780
C	9.65242476198275	-2.41468967770226	2.15627080102975
H	9.45793718442811	-3.12259461033862	2.95743079435875
H	10.67727058872842	-2.57930371639397	1.81183475679673
H	8.99215675270384	-2.65142359457352	1.32232525092045
C	10.55496992626269	-0.63334276568346	3.67755641170119
H	10.44342200886668	0.38331921041517	4.05794973027518
H	11.55327408540701	-0.71524587777917	3.24037143441963
H	10.50834850125926	-1.32605374757643	4.51655428644435
C	7.41938434017259	-1.30490354993292	4.88051806980616
C	7.88489117731263	-2.75835470885474	5.00559549511775
H	7.47357365110407	-3.38955646540274	4.21675375638383
H	7.53776146020200	-3.16280008135916	5.96088567302876
H	8.96925260738748	-2.84824385170654	4.98987671807607
C	8.07628900131697	-0.45101823164725	5.97596122882692
H	9.16233124670409	-0.49409674670916	5.94467667773434
H	7.75809888524325	-0.81524836187773	6.95622593007433
H	7.77804830021901	0.59369239624322	5.89577810687427
C	5.89770225996568	-1.26922870928592	5.10011667393738
H	5.48661626209902	-0.26867602160735	4.98744333719119
H	5.68746819177024	-1.60766602076537	6.11797056044650
H	5.36733424565419	-1.92877432487107	4.41484595977680
C	3.95207682741512	2.23978739455579	3.29035951347449
C	2.67560744675355	2.98233004684640	3.55483358077393
H	2.84385211393906	3.83700063977436	4.20450664536632
H	1.93286210313318	2.33556075577274	4.02607165075557
H	2.23941714352699	3.34768839064675	2.62114800383743
C	5.28240518813619	3.87072731064377	4.43730839439648
C	5.31521811620494	5.13500278350301	3.82270697693191
C	5.21352202436995	5.31276461858862	2.31921616893303
H	5.19911051215496	4.32453662310366	1.86251140773506

C	6.43542203707309	6.05226177811331	1.76101964779557
H	6.48232407580923	7.07788117707520	2.13450527729372
H	6.38577303641936	6.09084150855815	0.67156206764556
H	7.35803204664619	5.54060813145456	2.03063171039726
C	3.92135557052084	6.04198417539787	1.92461295723242
H	3.03689986815553	5.53410387368959	2.30936033571267
H	3.83620243022438	6.09906007423763	0.83733266852709
H	3.91278579416972	7.06263772535484	2.31395277644768
C	5.44436713460896	6.26187280064789	4.63454669657629
H	5.46628796628315	7.24162059343042	4.17496135384427
C	5.54687970291479	6.15414128021242	6.01068314484405
H	5.64357597862287	7.04278621603820	6.62162061010556
C	5.53468114285742	4.89950081546636	6.60248759582760
H	5.62618721856234	4.82009435792616	7.67716385436022
C	5.40824311408904	3.74443754573889	5.83651165615500
C	5.37206984245590	2.37894561684759	6.49859743037760
H	5.73986857264562	1.66616945616090	5.76018741354880
C	6.26950475664028	2.27720829009654	7.73403798480028
H	7.28779760816369	2.60623559020187	7.52024453037580
H	6.31009326338273	1.24300126988910	8.08065836848567
H	5.88919617958311	2.87896254079137	8.56153711897772
C	3.93759359719880	1.96289244349680	6.85189974334602
H	3.50244228863653	2.66071640081304	7.57121348290680
H	3.92560753452117	0.96487167307441	7.29671680744257
H	3.29881691539092	1.94581688937778	5.97102978196640
Br	7.61723345221642	2.71373536630315	0.79868582698357
